# Room-temperature borylation and one-pot two-step borylation/Suzuki–Miyaura cross-coupling reaction of aryl chlorides†

**DOI:** 10.1039/c8ra01381k

**Published:** 2018-04-11

**Authors:** Hong Ji, Li-Yang Wu, Jiang-Hong Cai, Guo-Rong Li, Na-Na Gan, Zhao-Hua Wang

**Affiliations:** Key Laboratory of Molecular Target & Clinical Pharmacology, School of Pharmaceutical Sciences & the Fifth Affiliated Hospital, Guangzhou Medical University Guangzhou 511436 P. R. China dljih@126.com +86-020-37103255; School of Basic Sciences, Guangzhou Medical University Guangzhou 511436 P. R. China

## Abstract

A highly efficient room-temperature borylation strategy of aryl chlorides is described. Utilizing Buchwald's second-generation preformed catalyst, boronate esters were obtained for a wide range of substrates in high yield. The method was also applied to Suzuki–Miyaura cross-coupling reaction in a one-pot two-step sequential manner, providing a facile and convenient access to the direct synthesis of biaryl compounds from aryl chlorides.

## Abstract 2

A highly efficient room-temperature strategy for borylation and one-pot two-step borylation/Suzuki–Miyaura cross-coupling reaction of aryl chlorides have been developed.

## Graphical abstract

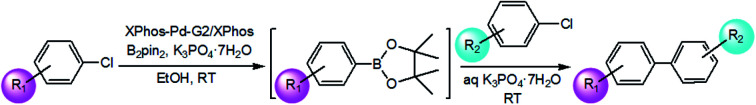

## Introduction

Aryl boronic acids and their derivatives are versatile chemical building blocks and intermediates in organic synthesis. These compounds play an important role in the preparation of various carbon-oxygen, carbon-nitrogen, and carbon–carbon bonds.^1^ They are widely utilized in transition-metal-catalyzed cross-coupling reactions, particularly Suzuki–Miyaura cross-coupling reaction. Suzuki–Miyaura cross-coupling reaction is one of the most powerful methods for the synthesis of biaryl motifs, which are frequently present in medicines, agrochemicals, conjugate polymers and other functional materials.^2^ Arylboronate esters are considered to be desirable for coupling reactions due to their high-stability, low-toxicity and functional group compatibility. Traditional methods for arylboronate esters preparation require aryllithium or Grignard reagents from aryl iodides or bromides.^3^ However, the harsh reaction conditions, poor functional group tolerance,^4^ and incompatibility with readily available aryl chlorides^2*b*,5^ greatly limit their application.

In the past two decades, the transition-metal-catalyzed borylation of aryl halides has emerged^6^ as an efficient alternative to aryl boronic acids and esters containing various functional groups. Since Pd-catalyzed method was pioneered by Miyaura,^7^ a series of processes have been developed for the conversion of aryl iodides, bromides and triflates to the corresponding boronate esters.^8^ Compared with common precursors such as aryl iodides, bromides, and triflates, aryl chlorides are less active but more attractive due to their relatively low cost and broad availability.^9^ Therefore, extensive efforts have been devoted to the development of highly reactive Pd catalyst systems for the conversion of the challenging aryl chlorides to their corresponding boronate esters, which has led to the development of sterically bulky, electron-rich ligands, such as trialkylphosphanes,^10^ dialkylbiarylphosphanes,^11^ monophosphines,^12^ indolylphosphines,^13^ and other heterocyclic phosphines.^14^ Some new ligands^13*b*,15^ have also been found to promote unactivated aryl chlorides into suitable coupling partners in the Suzuki–Miyaura cross-coupling reaction. Additionally, progress towards new catalysts for Suzuki–Miyaura cross-coupling reactions has also been made. Buchwald *et al.* previously reported a new class of Pd precatalysts bearing biarylphosphine ligands (XPhos-Pd-G1) for facile C–N cross-coupling reactions.^16^ Two years later, Buchwald's second generation XPhos precatalys (XPhos-Pd-G2) was synthesized and utilized in Suzuki–Miyaura coupling reactions.^17^ The second generation precatalyst allowed rapid formation of the requisite Pd(0) species at lower temperatures in the presence of a weak base, greatly expanding the general scope of Pd-catalyzed Suzuki–Miyaura cross-coupling reactions.

The next major advance came in 2007, when Buchwald and co-worker successfully demonstrated the general borylation of aryl chlorides, and extended the method, for the first time, to the direct “one-pot” synthesis of biaryl compounds from two aryl chlorides.^11*a*^ Later, several improved approaches to borylation were extended to the one-pot borylation/Suzuki–Miyaura cross-coupling reaction of aryl halides,^11*c*,13*d*,18^ and they circumvented the limitations of the Suzuki–Miyaura cross-coupling reaction. The one-pot method allows the coupling of two aryl halides in a simple and efficient manner, therefore avoiding the use of the boronic acid, which is expensive, easily decomposed, and often commercially unavailable.

Although significant progress has been made, these protocols still have several disadvantages. In most borylation, the Suzuki–Miyaura cross-coupling reaction and one-pot synthesis cases included, high temperature (100 to 120 °C) and long reaction times (24–48 h) are usually conducted in solvents that are not environmentally friendly (dioxane, toluene, DMF). Some of the efficient catalyst systems are not commercially available and high catalyst loads are necessary for many substrates to result in high yields. Additionally, excess boron coupling component is often required in the first step, and a second loading of catalyst and base are needed in the second step. Thus, the development of a practical and highly efficient borylation as well as a direct one-pot synthesis of biaryl compounds from aryl chlorides remains highly desirable. To date, few examples of aryl chlorides are documented to undergo borylation or one-pot borylation/Suzuki–Miyaura cross-coupling at room temperature.^11*a*^ Herein, we reported for the first time a room temperature procedure for the efficient conversion of aryl chlorides into the corresponding boronate esters as well as the subsequent two-step, one-pot synthesis of symmetrical and unsymmetrical biaryl compounds from two aryl chlorides.

## Results and discussion

**Fig. 1:**
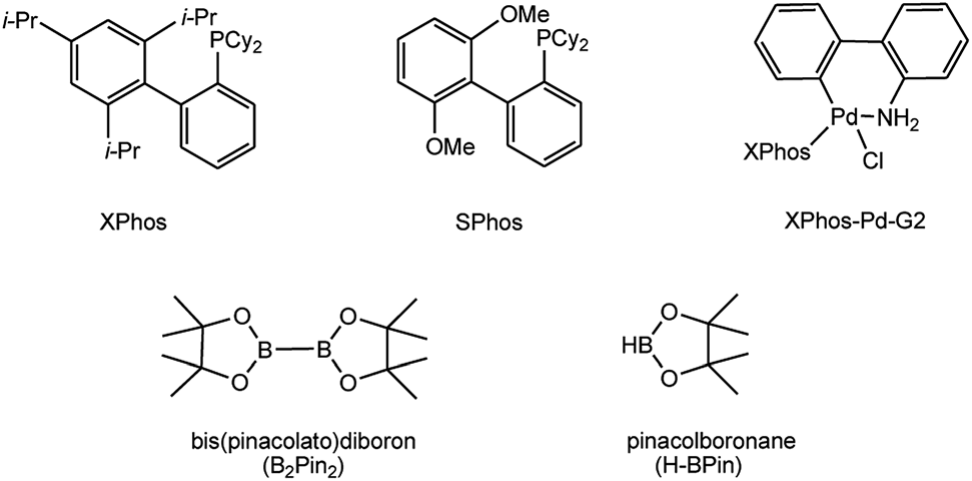
Ligands and boron reagents for Pd-catalyzed borylation of aryl chlorides.

To develop a facile borylation of aryl chlorides at room temperature, we initially chose the simple 4-chlorotoluene (1a) as the model substrate. Since the reported Pd-catalyzed borylation of aryl halides are often carried out at elevated temperatures in a sealed tube, we tested feasibility of the borylation at room temperature using the catalyst systems shown in Table 1. Using bis(pinacolato)diboron (B_2_Pin_2_) or pinacolboronane (H-BPin)^6*e*,8*e*,8*f*,11*a*,19^ as the borylating source (Fig. 1), the Pd_2_dba_3_/XPhos^11*a*,20^ and Pd(OAc)_2_/XPhos systems^6*b*,11*a*,21^ failed at room temperature, but facilitated the conversion of 1a at elevated temperatures (Table 1, entries 1–2 and 4–5). No product was obtained when PdCl_2_(dppf)_2_ ^6*a*,15*f*,22^ was utilized as the catalyst even at 110 °C (Table 1, entry 3). Although Pd(OAc)_2_/SPhos^11*a*^ catalyzed the borylation of 1a in 48 h with a 38% yield of 2a (Table 1, entry 6), not much improvement in yield was observed after our attempt to optimize the reaction conditions. When XPhos-Pd-G2, the second generation XPhos aminobiphenyl preformed catalyst (Fig. 1),^17^ was utilized in the reaction, the borylation of 1a occurred at room temperature with a low yield (Table 1, entries 7 and 8).

**Table 1:** Palladium-catalyzed borylation of 4-chlorotoluene^a^

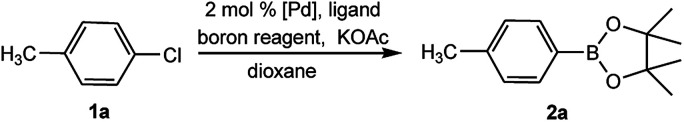					
Entry	Boron reagent	Catalyst	Ligand	Time (h)	Yield^b^ (%)
1	B_2_pin_2_	Pd_2_dba_3_	XPhos	10	0, 84^c^
2	H-Bpin	Pd_2_dba_3_	XPhos	10	0^d^, 45^c^^,^^d^
3	B_2_pin_2_	PdCl_2_(dppf)_2_	—	10	0^c^^,^^e^
4	B_2_pin_2_	Pd(OAc)_2_	XPhos	10	0, 72^c^
5	H-Bpin	Pd(OAc)_2_	XPhos	10	0^d^, 37^c^^,^^d^
6	B_2_pin_2_	Pd(OAc)_2_	SPhos	48	38
7	B_2_pin_2_	XPhos-Pd-G2	XPhos	1.0	17
8	H-Bpin	XPhos-Pd-G2	XPhos	1.0	11^d^

a Reaction conditions: 1a (1.0 equiv.), boron reagent (3.0 equiv.), KOAc (3.0 equiv.), dioxane (0.5 M), cat. [Pd]/ligand = 1 : 2, RT.

b Isolated yield.

c Sealed tube, 110 °C.

d Et_3_N as solvent.

e DMF as solvent.

After extensive research into the reaction conditions, we found that a combination of XPhos-Pd-G2/XPhos and B_2_Pin_2_ was exceptionally active. Among organic solvents surveyed, EtOH was found to be the best solvent for the room temperature version of this transformation. Compared with those commonly used solvents for borylation reactions, EtOH provided a fairly high yield (Table 2, entries 1–6). In the screening of bases, KOAc was found to be superior to other bases (Table 2, entries 6–10). The use of K_2_CO_3_ or K_3_PO_4_ led to the full conversion of 1a, despite the unavoidable formation of approximately 11–18% of the homocoupling by-product^23^ (Table 2, entries 7 and 10). Furthermore, we found when K_3_PO_4_·7H_2_O was utilized *in lieu* of KOAc, near-quantitative yield was achieved (Table 2, entry 11). At relatively low quantities of catalyst (0.5 mol%) and ligand (0.25 mol%), no effect on the product yield was observed. The absence of XPhos was equally effective but led to a decrease in the yield to 64% (Table 2, entry 12).

**Table 2:** Reaction parameters optimization for borylation of 4-chlorotoluene^a^

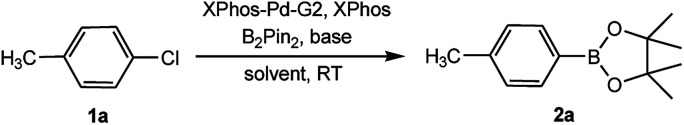			
Entry	Solvent	Base	Yield^b^ (%)
1	Dioxane	KOAc	17
2	Toluene	KOAc	38
3	THF	KOAc	24
4	Et_3_N	—	Trace^c^
5	*t*-BuOH	KOAc	19
6	EtOH	KOAc	82
7	EtOH	K_2_CO_3_	72
8	EtOH	Cs_2_CO_3_	51
9	EtOH	CsOAc	34
10	EtOH	K_3_PO_4_	50
11	EtOH	K_3_PO_4_·7H_2_O	98
12	EtOH	K_3_PO_4_·7H_2_O	98^d^, 64^e^

a Reaction conditions: 1a (1.0 equiv.), B_2_pin_2_ (2.0 equiv.), XPhos-Pd-G2 (2 mol%), XPhos (4 mol%), base (3.0 equiv.), solvent (0.5 M), 30 min.

b Isolated yield.

c Et_3_N as solvent.

d B_2_pin_2_ (1.2 equiv.), XPhos-Pd-G2 (0.5 mol%), XPhos (0.25 mol%).

e XPhos-Pd-G2 (0.5 mol%) was used without XPhos.

With the optimized conditions in hand, we explored the scope of aryl chlorides in the borylation reaction. A range of aryl chlorides were examined and the results are listed in Table 3. The catalyst system efficiently transformed nonactivated aryl chlorides such as *o*-chloroaniline, *m*-chloroanisole, *p*-chlorobenzyl alcohol and *p*-chlorophenol to the corresponding boronate esters within 0.5–2.0 h with excellent yield (Table 3, entries 1–4). For activated aryl chlorides, common functional groups such as –CHO, –COMe, –NO_2_ and –CN were compatible under the mild reaction conditions (Table 3, entries 5–9). Additionally, unprotected amines and hydroxyl groups were well tolerated, leading to good to excellent yields (Table 3, entries 2, 3, 4 and 10). *p*-Chloroacetophenone and *m*-nitrochlorobenzene showed better yields under a higher catalyst loadings (1 mol%) and longer reaction times (Table 3, entry 7 and 8). In addition, heteroaryl chlorides were also readily transformed to the desired products with good to excellent yields (Table 3, entries 11–13).

**Table 3:** Palladium-catalyzed borylation of aryl chlorides at room temperature^a^

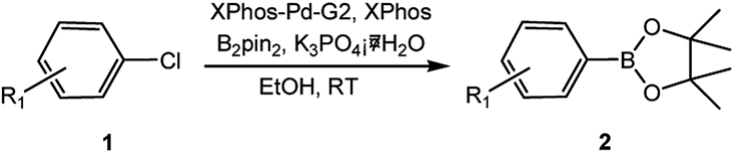				
Entry	Ary chloride	Pd (mol%)	Time (h)	Yield^b^ (%)
1	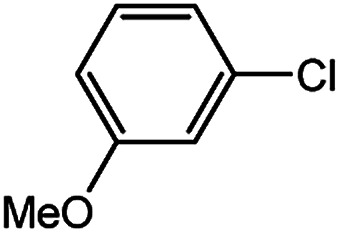	0.5	0.5	92
2	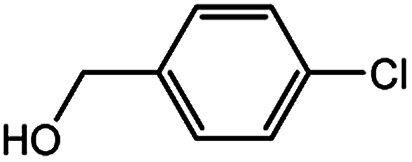	0.5	2.0	96
3	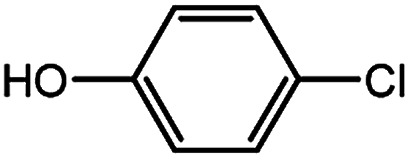	0.5	1.0	74
4	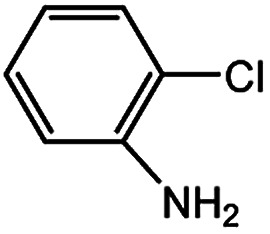	0.5	1.0	84
5	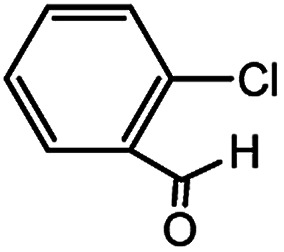	0.5	1.0	72
6	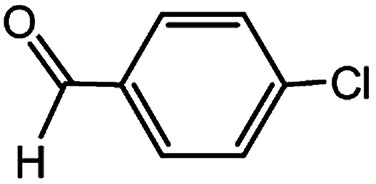	0.5	4.0	83^c^
7	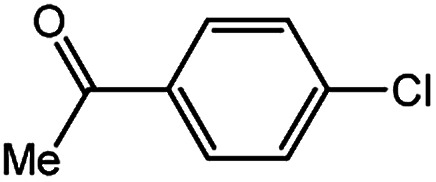	1.0	8.0	85^c^
8	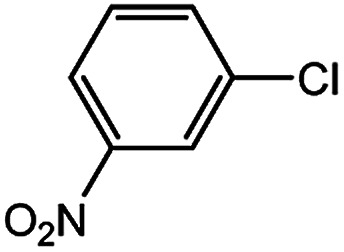	1.0	6.0	82^c^
9	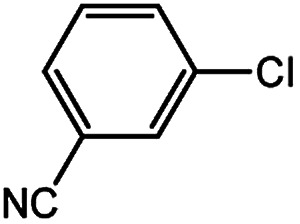	0.5	1.0	93
10	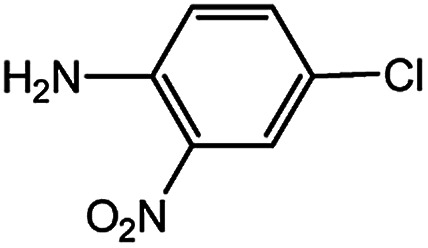	1.0	1.5	63
11	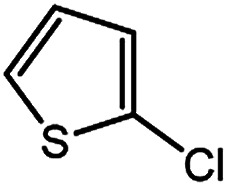	1.0	3.0	67
12	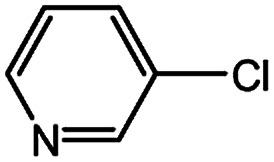	0.5	0.5	90
13	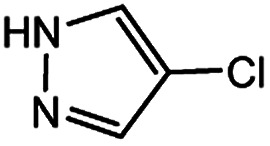	0.5	0.5	88

a Reaction conditions: 1 (1.0 equiv.), B_2_pin_2_ (1.2 equiv.), K_3_PO_4_·7H_2_O (3.0 equiv.), XPhos-Pd-G2 (0.5 mol%), XPhos (0.25 mol%), EtOH (0.5 M).

b Isolated yield.

c XPhos-Pd-G2 (1.0 mol%), XPhos (0.5 mol%).

In our initial studies on the borylation of aryl chlorides, we found that symmetrical biaryl compounds could be generated *via* Suzuki–Miyaura cross-coupling reaction in the presence of K_3_PO_4_ or K_2_CO_3_. These results provided an opportunity to apply borylation for the sequential synthesis of symmetrical and unsymmetrical biaryl compounds. Under the optimized conditions of 0.5 mol% Pd catalyst loading and a Pd-to-ligand ratio of 1 : 1 (Table 4), the direct conversion of 4-chlorotoluene into 4,4′-dimethylbiphenyl was accomplished in 97% yield at room temperature (entry 3a). This approach was also applicable to the homocoupling of the electron-deficient chloride (4-chloroacetophenone, entry 3b) or the electron-rich aryl chlorides (2-chloroanisole and 2-chlorophenol, entries 3c, 3d), leading to 68–83% yield. Sterically hindered 2-chlorotoluene gave 74% yield of the homocoupling product when H_2_O (0.3 ml) was added into the reaction mixture after 2 h (entry 3e). Heteroaryl chlorides, such as 4-chloropyridine and 2-chlorothiophene, were successful in affording the desired products (entries 3f and 3g) with good to excellent yield.

**Table 4:** Palladium-catalyzed synthesis of symmetrical biaryl compounds^a^

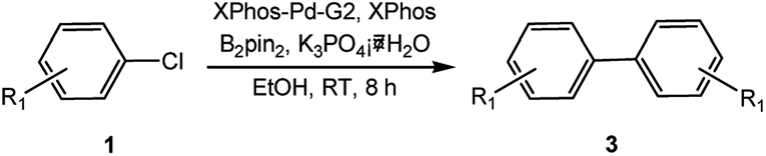
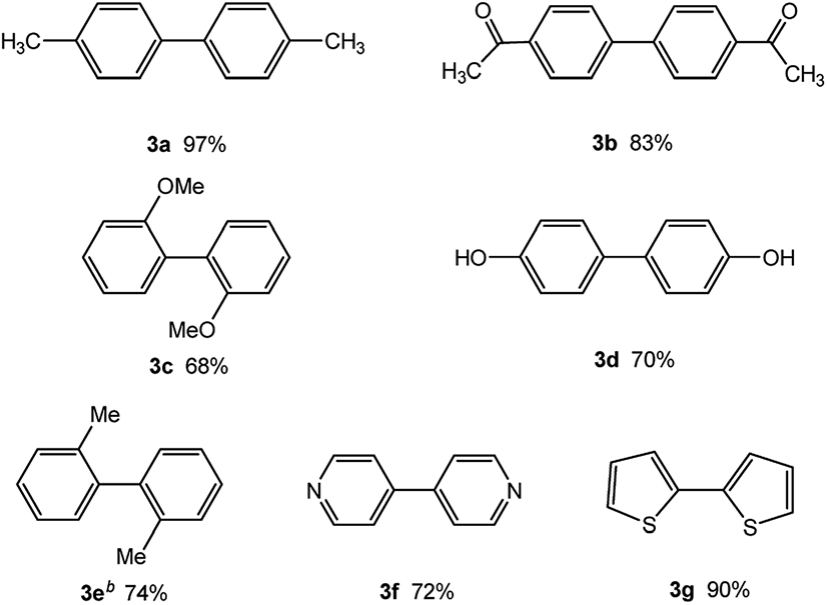

a Reaction conditions: 1 (1.0 equiv.), B_2_pin_2_ (0.5 equiv.), K_3_PO_4_·7H_2_O (3.0 equiv.), XPhos-Pd-G2 (0.5 mol%), XPhos (0.5 mol%), EtOH (0.20 M). Isolated yield.

b H_2_O (0.3 ml) added into the reaction mixture after 2 h.

In light of further application of the borylation methodology in the subsequent synthesis of unsymmetrical biaryls, we were prompted to examine a two-step one-pot procedure. The Suzuki–Miyaura cross-coupling reaction was successfully achieved at room temperature by adding a second aryl chloride and aqueous K_3_PO_4_·7H_2_O after the borylation reaction of the first chloride 1m. This reaction sequence was completed without isolation of the borylated intermediates. An excess equivalent of neither the boronate esters nor the second aryl halides component was required in the reaction. Additionally, no additional catalyst was added before conducting the second reaction of the sequence. As illustrated in Table 5, the protocol exhibited high catalytic activity for the Suzuki–Miyaura cross-coupling reaction at room temperature and tolerated a wide range of substrates. Both electron-rich and electron-deficient aryl chlorides as the second chlorides afforded good to excellent yields (Table 5, entries 4a, 4d and 4e). The free amino group on the phenyl ring had almost no effect on Suzuki–Miyaura cross-coupling (Table 5, entry 4b). The traditionally challenging trifluoromethyl proceeded very well in the coupling process with 1m under the reaction conditions (Table 5, entry 4c). Additionally, heteroaryl chlorides also gave arylation products in good yields. When 3-chloropyridine, 6-chloroindole and 4-chloro-1-methylpyrazole were used in the coupling reaction, their corresponding biaryl products were obtained in 64%, 80% and 76% yield, respectively (Table 5, entries 4f, 4g, 4h). This work is the first direct room temperature synthesis of unsymmetrical biaryl compounds from two aryl chlorides.

**Table 5:** Palladium-catalyzed one-pot two-step synthesis of unsymmetrical biaryl compounds^a^

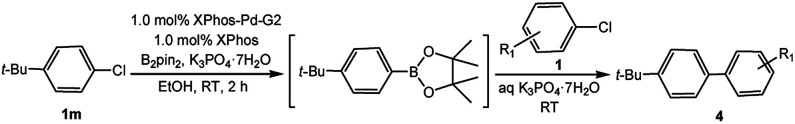
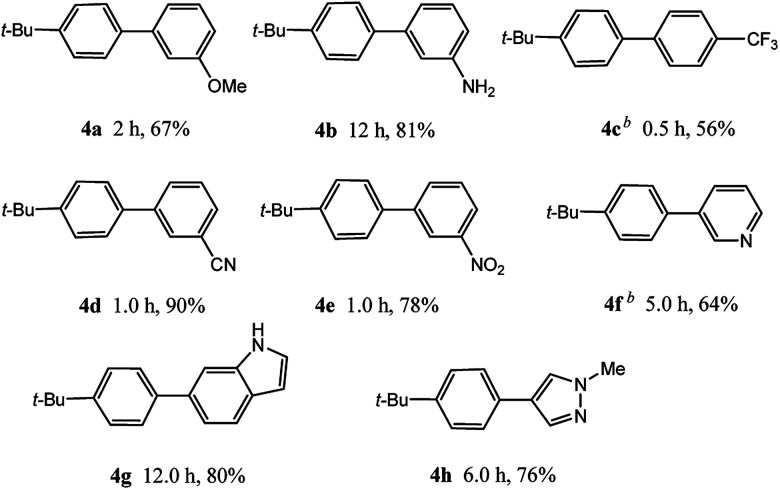

a Reaction conditions: (a) 1m (1.1 equiv.), XPhos-Pd-G2 (1.0 mol%), XPhos (1.0 mol%), B_2_pin_2_ (1.2 equiv.), K_3_PO_4_·7H_2_O (3.0 equiv.), EtOH (0.275 M), RT, 2 h; (b) second aryl chloride (1.0 equiv.),3.0 M aq. K_3_PO_4_·7H_2_O (3.0 equiv.), RT. Isolated yield based on 1m.

b XPhos-Pd-G2 (1.5 mol%) and XPhos (1.5 mol%).

Encouraged by the success encountered with the aforementioned strategy, we further explored its synthetic utility. Since the two aryl chlorides used in the one-pot synthesis of unsymmetrical biaryls have distinguishing features, the order in which the chlorides are used can have a significant effect on the result of the reaction. Therefore, two strategies of forward-order and reverse-order were attempted, which allowed a choice in the addition order of the two chlorides in the reaction. As shown in Table 6, after the first chloride was subjected to borylation, the second chloride was used as the electrophile in the Suzuki–Miyaura cross-coupling reaction, and then the order was reversed. The first chloride borylated in the first step in the forward-order strategy could be used as the electrophile in the second step in the reverse-order strategy. We found that unprotected phenol and amine resulted in significantly higher yields when they were used in the first step (Table 6, entries 1 and 4). Aryl chlorides bearing a trifluoromethyl or nitro group performed better when used as the second chloride (Table 6, entries 2 and 4). However, aldehydes provided superior results when used in the first step (Table 6, entry 3). Additionally, no reduction of the aldehyde was observed, as previously reported in the haloaryl borylation.^20*b*,21^ In the case of the coupling of heteroaryls, the yield was unexpectedly better if the electron-deficient partner was borylated (Table 6, entries 6 and 7). 2-Chloroquinoline, however, was an exception and led to lower yields when used in the first step (Table 6, entry 5). This study illustrates that exploring the forward-order and reverse-order strategy on a small scale could be a method for finding the highest yielding order before scaling up the reaction.

**Table 6:** Comparison of forward-order and reverse-order strategies in synthesis of unsymmetrical biaryl compounds^a^

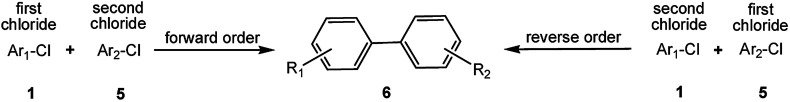					
Entry	Ar_1_–Cl	Ar_2_–Cl	Product	Forward yield^b^ (%)	Reverse yield^b^ (%)
1	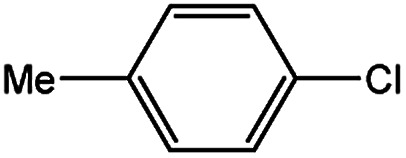	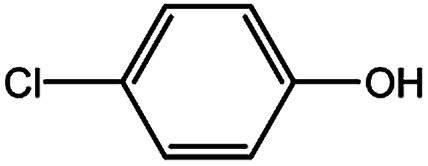	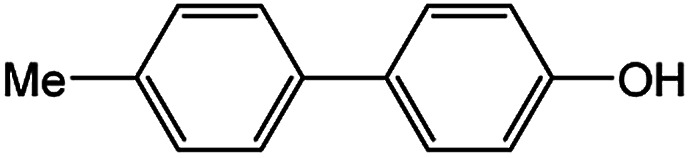	87	98
2	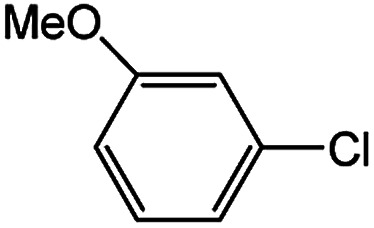	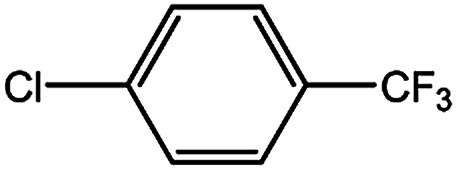	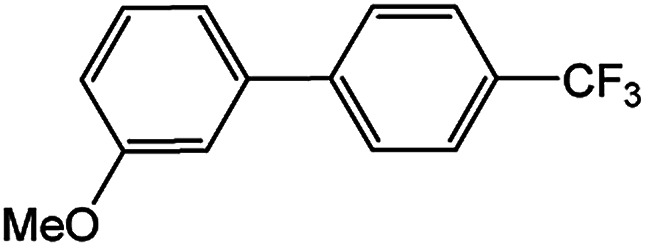	84	25
3	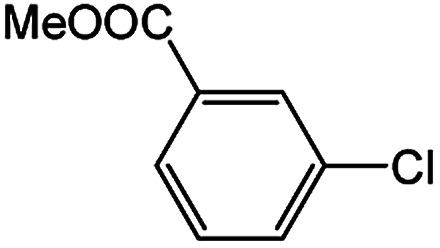	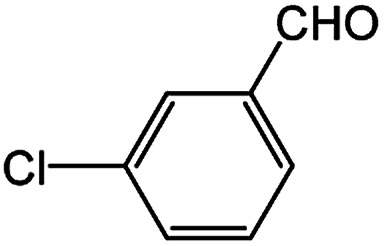	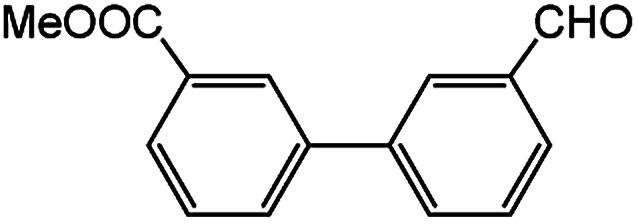	65	93
4	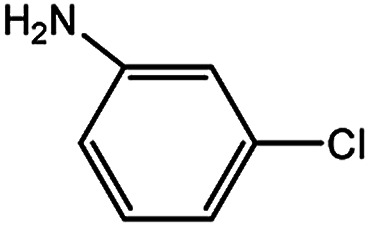	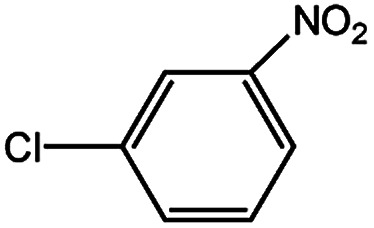	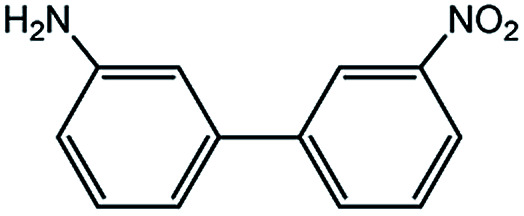	91^c^	20
5	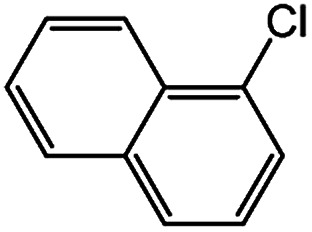	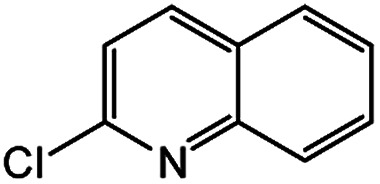	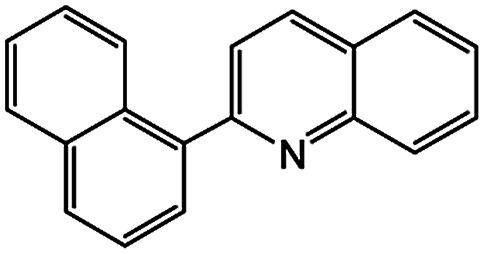	77^c^	42^c^
6	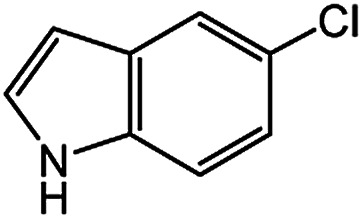	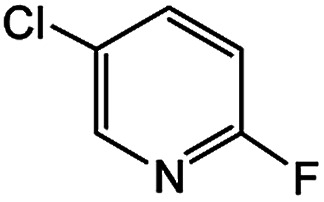	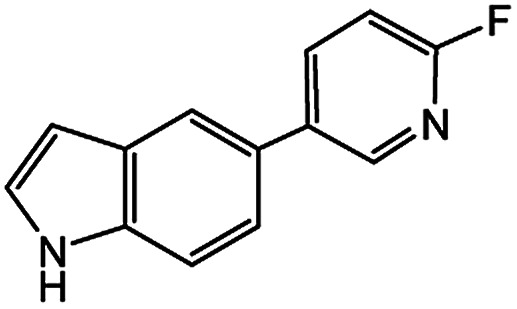	38^d^	79^c^
7	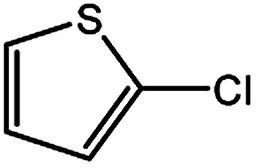	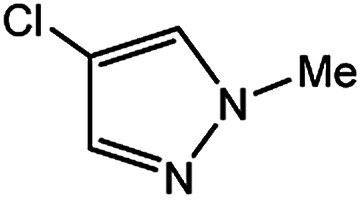	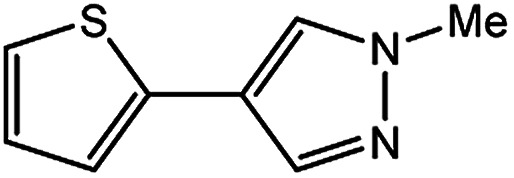	35^c^	85^c^

a General conditions: (a) first chloride (1.1 equiv.), XPhos-Pd-G2 (1.0 mol%), XPhos (1.0 mol%), B_2_pin_2_ (1.2 equiv.), K_3_PO_4_·7H_2_O (3.0 equiv.), EtOH (0.275 M), RT, 2 h; (b) second chloride (1.0 equiv.), 3.0 M aq. K_3_PO_4_·7H_2_O (3.0 equiv.), RT, 8 h.

b Yield of isolated product.

c 15 h for the second step.

d 6 h for the second step.

## Conclusions

In summary, an efficient Pd-catalyzed room temperature synthesis of diverse and functionalized arylboronate esters has been developed. This method allows borylation of widely accessible aryl chlorides, and was also successfully applied to one-pot two-step borylation/Suzuki–Miyaura cross-coupling reactions, providing a facile synthetic approach to symmetrical and unsymmetrical biaryl compounds. Compared with previous palladium-catalyzed Suzuki–Miyaura cross-coupling methods, this new protocol allows traditionally challenging aryl chlorides to act as coupling partners. Boronic acids no longer have to be purchased or isolated. Because of the room temperature conditions and reduced reaction times, functional group tolerance was also greatly improved. Further, several advantages, such as extremely mild reaction conditions, low catalyst loading, and the use of a more environmentally friendly solvent, makes this method very attractive.

## Conflicts of interest

There are no conflicts to declare.

## Supplementary Material

RA-008-C8RA01381K-s001
