# Supplementary material for: Room-temperature borylation and one-pot two-step borylation/Suzuki–Miyaura cross-coupling reaction of aryl chlorides
Source: RSC Adv. 2018 Apr 11;8(25):13643–8. doi: 10.1039/c8ra01381k (PMC9079898; doi:10.1039/c8ra01381k)
Supplement: RA-008-C8RA01381K-s001 [file RA-008-C8RA01381K-s001.pdf]

Electronic Supplementary Material (ESI) for RSC Advances.
This journal is © The Royal Society of Chemistry 2018

Supporting Information

Room-temperature borylation and one-pot two-step borylation/Suzuki-Miyaura cross-coupling reaction of aryl chlorides

Hong Ji,*[a] Li-Yang Wu,[a] Jiang-Hong Cai,[a] Guo-Rong Li,[a] Na-Na Gan[a] and Zhao-Hua Wang[b]

[a]Key Laboratory of Molecular Target & Clinical Pharmacology, School of Pharmaceutical Sciences & the Fifth Affiliated Hospital, Guangzhou Medical University, Guangzhou 511436, P. R. China

E-mail: dljih@126.com

[b]School of Basic Sciences, Guangzhou Medical University, Guangzhou 511436, P. R. China

Table of contents

General information........S2

General procedure for screening........S2

General procedure for optimization of reaction parameters........S2

General procedure for borylation of aryl chlorides........S3

General procedure for synthesis of symmetrical biaryl compounds........S7

General procedure for synthesis of asymmetrical biaryl compounds........S8

Reference........S13

^{1}H and ^{13}C NMR spectra of all compounds........S15

General Information

All the reactions were carried out under N_2 atmosphere. Melting points were determined by the open capillary tube method and are uncorrected. ^{1}H and ^{13}C NMR spectra were recorded on Bruker AV Ⅲ (300 MHz) and JOEL ECA-400 spectrometers by using $CDCl_3$ or DMSO-d_6 as the solvent and tetramethylsilane (TMS) as the internal standard. Silica gel (200-300 mesh) was used for column chromatography.

General procedure for screening

A glass vessel was charged with Palladium Catalyst (0.02 mmol), ligand (0.04 mmol), boron reagent (3.00 mmol), and KOAc (294 mg, 3.00 mmol) in dioxane (2 ml). The mixture under nitrogen was stirred for 10 min, followed by the addition of **1a** (127 mg, 1.00 mmol). The reaction mixture was stirred at the indicated temperature for the indicated time in Table 1. The reaction solution was diluted with ethyl acetate (4 ml) and filtered through a thin pad of celite (eluting with ethyl acetate) and the eluent was concentrated under reduced pressure. The crude material so obtained was purified via column chromatography on silica gel (petroleum ether/ethyl acetate).

2-(4-methylphenyl)-4,4,5,5-tetramethyl-1,3,2-dioxaborolane (2a) [1]

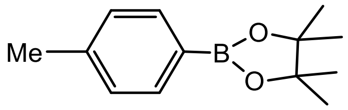

White solid, m. p. 53-55 ℃. ^{1}H NMR (400 MHz, $CDCl_3$) δ 7.71 (d, J = 7.8 Hz, 2H), 7.19 (d, J = 7.7 Hz, 2H), 2.37 (s, 3H), 1.34 (s, 12H). ^{13}C NMR (101 MHz, $CDCl_3$) δ 141.39, 134.73, 128.49, 83.57, 24.79, 21.72.

General procedure for optimization of reaction parameters

A glass vessel was charged with XPhos-Pd-G2 (15.7 mg, 0.02 mmol), XPhos (19 mg, 0.04 mmol), B_2pin_2 (508 mg, 2.00 mmol), and base (3.00 mmol) in the solvent (2 ml) indicated in the table 2. The mixture under nitrogen was stirred for 10 min, followed by the addition of **1a** (127 mg, 1.00 mmol). The reaction mixture was stirred at room

temperature for 30 minutes. The reaction solution was diluted with ethyl acetate (4 ml) and filtered through a thin pad of celite (eluting with ethyl acetate) and the eluent was concentrated under reduced pressure. The crude material so obtained was purified via column chromatography on silica gel (petroleum ether/ethyl acetate).

General procedure for borylation of aryl chlorides

A glass vessel was charged with XPhos-Pd-G2 (4 mg, 0.005 mmol), XPhos (1.2 mg, 0.0025 mmol), B_2pin_2 (305 mg, 1.20 mmol), and $K_3PO_4 \cdot 7H_2O$ (1.01 g, 3.00 mmol) in EtOH (2 ml). The mixture under nitrogen was stirred for 10 min, followed by the addition of **1** (1.00 mmol). The reaction mixture was stirred at room temperature until the reaction was complete as monitored by TLC. The reaction solution was diluted with ethyl acetate (4 ml) and filtered through a thin pad of celite (eluting with ethyl acetate) and the eluent was concentrated under reduced pressure. The crude material so obtained was purified via column chromatography on silica gel (petroleum ether/ethyl acetate).

2-(3-Methoxyphenyl)-4,4,5,5-tetramethyl-1,3,2-dioxaborolane (2b)[1] (Table 3, entry 1)

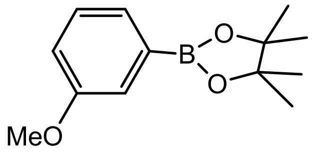

White solid, m. p. 106-107℃. ^{1}H NMR (300 MHz, $CDCl_3$) δ 7.43 – 7.37 (m, 1H), 7.33 (d, J = 3.5 Hz, 1H), 7.30 – 7.26 (m, 1H), 7.01 (ddd, J = 8.2, 2.8, 1.2 Hz, 1H), 3.83 (s, 3H), 1.34 (s, 12H). ^{13}C NMR (101 MHz, $CDCl_3$) δ 158.98, 128.88, 127.12, 118.66, 117.82, 83.75, 55.14, 24.80.

2-(4-Hydroxymethylphenyl)-4,4,5,5-tetramethyl-1,3,2-dioxaborolane (2c) [2] (Table 3, entry 2)

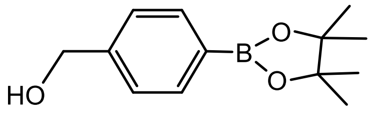

White solid, m. p. 122-124℃. ^{1}H NMR (400 MHz, $CDCl_3$) δ 7.81 (d, J = 8.0 Hz, 2H), 7.37 (d, J = 8.0 Hz, 2H), 4.72 (s, 2H), 1.35 (s, 12H). ^{13}C NMR (101 MHz, $CDCl_3$) δ

143.94, 134.95, 126.02, 83.78, 65.12, 24.79.

2-(4-Hydroxylphenyl)-4,4,5,5-tetramethyl-1,3,2-dioxaborolane (2d) [1] (Table 3, entry 3)

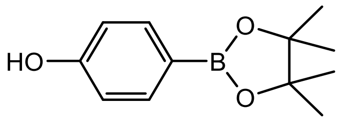

White solid, m. p. 109-110℃. ^{1}H NMR (300 MHz, $CDCl_3$) δ 7.70 (d, J = 8.5 Hz, 2H), 6.87 – 6.77 (m, 2H), 1.33 (s, 12H).. ^{13}C NMR (101 MHz, $CDCl_3$) δ 158.78, 136.71, 114.96, 83.79, 24.72.

2-(2-Aminophenyl)-4,4,5,5-tetramethyl-1,3,2-dioxaborolane (2e) [3] (Table 3, entry 4)

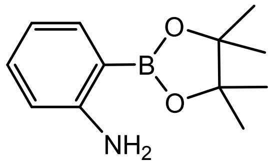

White solid, m. p. 89-90℃. ^{1}H NMR (400 MHz, $CDCl_3$) δ 7.61 (dd, J = 7.4, 1.7 Hz, 1H), 7.21 (ddd, J = 8.1, 7.2, 1.7 Hz, 1H), 6.67 (td, J = 7.3, 1.0 Hz, 1H), 6.61 – 6.57 (m, 1H), 4.73 (s, 2H), 1.34 (s, 12H). ^{13}C NMR (101 MHz, $CDCl_3$) δ 153.58, 136.73, 132.67, 116.80, 114.70, 83.43, 24.85.

2-(4,4,5,5-Tetramethyl-1,3,2-dioxaborolan-2-yl)-benzaldehyde (2f) [1] (Table 3, entry 5)

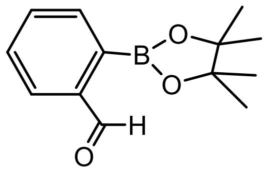

White solid, m. p. 71-73℃. ^{1}H NMR (400 MHz, $CDCl_3$) δ 10.55 (s, 1H), 7.98 – 7.93 (m, 1H), 7.88 – 7.84 (m, 1H), 7.61 – 7.52 (m, 2H), 1.39 (s, 12H). ^{13}C NMR (101 MHz, $CDCl_3$) δ 194.46, 141.11, 135.34, 132.86, 130.60, 127.75, 84.26, 24.72.

4-(4,4,5,5-Tetramethyl-1,3,2-dioxaborolan-2-yl)-benzaldehyde (2g) [3] (Table 3, entry 6)

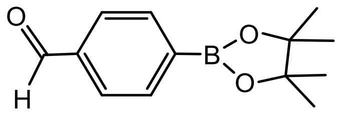

White solid, m. p. 55-57℃. ^{1}H NMR (400 MHz, $CDCl_3$) δ 10.05 (s, 1H), 7.97 (d, J = 7.9 Hz, 2H), 7.87 (d, J = 8.0 Hz, 2H), 1.37 (s, 12H). ^{13}C NMR (101 MHz, $CDCl_3$) δ 192.79, 137.95, 135.16, 128.70, 84.30, 24.82.

1-(4-(4,4,5,5-Tetramethyl-1,3,2-dioxaborolan-2-yl)phenyl)-ethanone (2h) [1] (Table 3, entry 7)

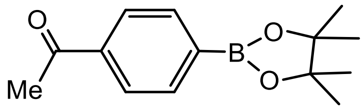

White solid, m. p. 63-65℃. ^{1}H NMR (400 MHz, $CDCl_3$) δ 7.92 (td, J = 8.2, 6.5 Hz, 4H), 2.63 (s, 3H), 1.36 (s, 12H). ^{13}C NMR (101 MHz, $CDCl_3$) δ 198.56, 138.82, 134.85, 127.25, 84.17, 26.82, 24.82.

2-(3-Nitrophenyl)-4,4,5,5-tetramethyl-1,3,2-dioxaborolane (2i) [1] (Table 3, entry 8)

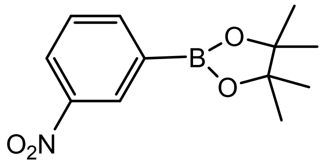

White solid, m. p. 108-109℃. ^{1}H NMR (300 MHz, $CDCl_3$): δ 8.64 (d, J = 1.7 Hz, 1H), 8.30 (ddd, J = 8.2, 2.4, 1.1 Hz, 1H), 8.10 (d, J = 7.3 Hz, 1H), 7.54 (t, J = 7.8 Hz, 1H), 1.37 (s, 12H). ^{13}C NMR (101 MHz, $CDCl_3$) δ 147.65, 140.62, 129.31, 128.70, 125.80, 84.52, 24.78.

3-(4,4,5,5-Tetramethyl-1,3,2-dioxaborolan-2-yl)benzonitrile (2j) [3] (Table 3, entry 9)

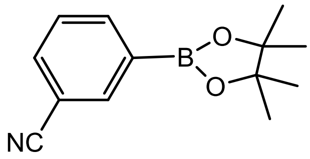

White solid, m. p. 93-94℃. ^{1}H NMR (300 MHz, $CDCl_3$) δ 8.09 (s, 1H), 8.04 – 7.96 (m, 1H), 7.72 (dt, J = 7.8, 1.5 Hz, 1H), 7.48 (d, J = 7.6 Hz, 1H), 1.35 (s, 12H). ^{13}C NMR (101 MHz, $CDCl_3$) δ 138.65, 138.28, 134.27, 128.30, 118.71, 111.95, 84.38,

24.76.

2-Nitro-4-(4,4,5,5-tetramethyl-1,3,2-dioxaborolan-2-yl) aniline (2k) [4] (Table 3, entry 10)

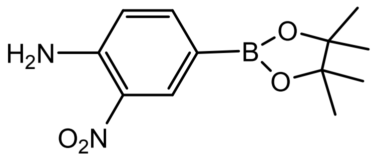

White solid, m. p. 176-177°C. ^{1}H NMR (300 MHz, $CDCl_3$) δ 8.59 (d, J = 1.1 Hz, 1H), 7.72 (dd, J = 8.3, 1.3 Hz, 1H), 6.77 (d, J = 8.3 Hz, 1H), 6.21 (s, 2H), 1.33 (s, 13H). ^{13}C NMR (101 MHz, $CDCl_3$) δ 146.43 (s), 141.04 (s), 133.70 (s), 131.99 (s), 117.93 (s), 83.95 (s), 24.80 (s).

4,4,5,5-Tetramethyl-2-(thiophen-2-yl)-1,3,2-dioxaborolan (2l) [1] (Table 3, entry 11)

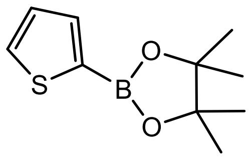

White solid, m. p. 76-78℃. ^{1}H NMR (300 MHz, $CDCl_3$) δ 7.65 (dd, J = 5.9, 4.1 Hz, 2H), 7.19 (dd, J = 4.6, 3.5 Hz, 1H), 1.35 (s, 12H). ^{13}C NMR (101 MHz, $CDCl_3$) δ 137.12, 132.33, 128.18, 84.03, 24.73.

3-(4,4,5,5-Tetramethyl-1,3,2-dioxaborolan-2-yl)pyridine (2m) [5] (Table 3, entry 12)

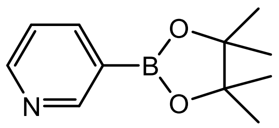

White solid, m. p. 102-104℃. ^{1}H NMR (400 MHz, $CDCl_3$) δ 8.95 (s, 1H), 8.67 (dd, J = 4.9, 1.9 Hz, 1H), 8.06 (d, J = 7.5 Hz, 1H), 7.32 – 7.27 (m, 1H), 1.36 (s, 12H). ^{13}C NMR (101 MHz, $CDCl_3$) δ 155.32, 151.87, 142.19, 123.05, 84.14, 24.78.

4-(4,4,5,5-Tetramethyl-1,3,2-dioxaborolan-2-yl)-*1H*-pyrazole (2m) [6] (Table 3, entry 13)

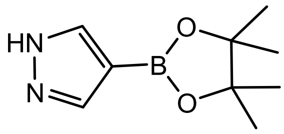

White solid, m. p. 143-145℃. ^{1}H NMR (400 MHz, $CDCl_3$) δ 7.91 (s, 2H), 1.34 (s,

12H). ^{13}CNMR (101 MHz, $CDCl_3$) δ 140.27 (s), 83.37 (s), 24.75 (s).

General procedure for synthesis of symmetrical biaryl compounds

A glass vessel was charged with XPhos-Pd-G2 (4 mg, 0.005 mmol), XPhos (2.4 mg, 0.005 mmol), B_2pin_2 (127 mg, 0.50 mmol), and $K_3PO_4·7H_2O$ (1.01 g, 3.00 mmol) in EtOH (5 ml). The mixture under nitrogen was stirred for 10 min, followed by the addition of aryl chlorides (1.00 mmol). The reaction mixture was stirred at room temperature for 8 h. The reaction solution was diluted with ethyl acetate (6 ml) and filtered through a thin pad of celite (eluting with ethyl acetate) and the eluent was concentrated under reduced pressure. The crude material so obtained was purified via column chromatography on silica gel (petroleum ether/ethyl acetate).

4,4'-Dimethylbiphenyl (3a) [7]

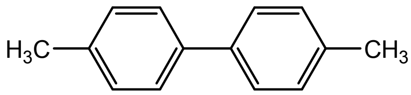

White solid, m. p. 118-119 °C. ^{1}H NMR (400 MHz, $CDCl_3$) δ 7.48 (d, J = 7.8 Hz, 4H), 7.24 (d, J = 7.9 Hz, 4H), 2.39 (s, 6H). ^{13}C NMR (101 MHz, $CDCl_3$) δ 138.18, 136.67, 129.40, 126.76, 21.08.

4,4'-Diacetylbiphenyl (3b) [8]

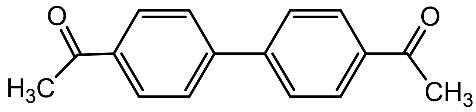

White solid, m. p. 193-195℃. ^{1}H NMR (400 MHz, $CDCl_3$) δ 8.07 (d, J = 8.3 Hz, 4H), 7.73 (d, J = 8.3 Hz, 4H), 2.66 (s, 6H). ^{13}C NMR (101 MHz, $CDCl_3$) δ 197.68, 144.21, 136.35, 128.95, 127.37, 26.73.

2,2'-Dimethoxybiphenyl (3c) [9]

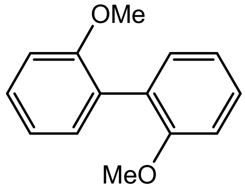

White solid, m. p.152-154℃. ^{1}H NMR (300 MHz, $CDCl_3$) δ 7.33 (ddd, J = 8.1, 7.5, 1.8 Hz, 2H), 7.26 (d, J = 1.8 Hz, 1H), 7.23 (d, J = 1.8 Hz, 1H), 7.00 (ddd, J = 8.2, 6.5,

2.8 Hz, 4H), 3.77 (s, 6H). ^{13}C NMR (101 MHz, $CDCl_3$) δ 156.97, 131.40, 128.55, 127.75, 120.28, 111.03, 55.62.

2,2'-Biphenol (3d) [10]

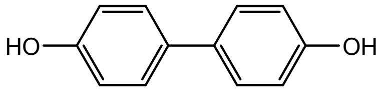

Grey solid, m. p. 281-282℃. ^1H NMR (400 MHz, DMSO-d_6) δ 9.64 – 9.30 (m, 2H), 7.63 – 7.26 (m, 4H), 6.88 (d, J = 8.4 Hz, 4H). ^{13}C NMR (101 MHz, DMSO-d_6) δ 156.35, 131.31, 127.12, 115.73.

2,2'-Dimethylbiphenyl (3e) [11]

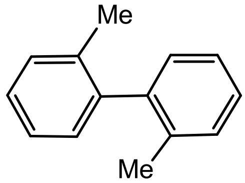

White solid, m. p. 90-92℃. ^1H NMR (300 MHz, $CDCl_3$) δ 7.28 – 7.18 (m, 6H), 7.14 – 7.07 (m, 2H), 2.08 – 2.02 (m, 6H). ^{13}C NMR (101 MHz, $CDCl_3$) δ 141.56, 135.78, 129.77, 129.25, 127.12, 125.50, 19.80.

4,4-Bipyridine (3f) [12]

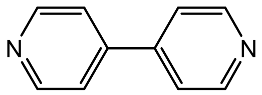

White solid, m. p. 109-111℃. ^1H NMR (400 MHz, $CDCl_3$) δ 8.75 (d, J = 5.9 Hz, 4H), 7.55 (d, J = 6.0 Hz, 4H). ^{13}C NMR (101 MHz, $CDCl_3$) δ 150.52, 145.35, 121.27.

2,2'-Bithiophene (3g) [5]

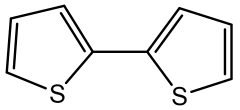

White solid, m. p. 32-33℃. ^1H NMR (400 MHz, $CDCl_3$) δ 7.21 (dd, J = 5.1, 0.8 Hz, 2H), 7.18 (dd, J = 3.6, 0.7 Hz, 2H), 7.02 (dd, J = 5.0, 3.7 Hz, 2H). ^{13}C NMR (101 MHz, $CDCl_3$) δ 137.30, 127.74, 124.31, 123.69.

General procedure for synthesis of asymmetrical biaryl compounds

A glass vessel was charged with XPhos-Pd-G2 (8 mg, 0.01 mmol), XPhos (4.8 mg, 0.01 mmol), B_2pin_2 (305 mg, 1.20 mmol), and $K_3PO_4{\cdot}7H_2O$ (1.01 g, 3.00 mmol) in EtOH (4 ml). The mixture under nitrogen was stirred for 10 min, followed by the addition of the first chloride (1.10 mmol). The reaction mixture was stirred at room temperature for 2 h. At this point the second aryl chloride (1.0 mmol) and $K_3PO_4{\cdot}7H_2O$ (3.0 M aqueous solution, 1.0 ml) were added to the reaction mixture under nitrogen. The reaction mixture was stirred at room temperature for the time indicated in Table 5 and 6. The reaction solution was diluted with ethyl acetate (5 ml) and filtered through a thin pad of celite (eluting with ethyl acetate) and the eluent was concentrated under reduced pressure. The crude material so obtained was purified via column chromatography on silica gel (petroleum ether/ethyl acetate).

3-Methoxy-4'-(*tert*-butyl)-1,1'-biphenyl (4a) [13]

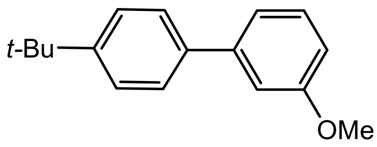

White solid, m. p. 107-109℃. ^{1}H NMR (300 MHz, CDCl$_3$) δ 7.58 – 7.41 (m, 4H), 7.34 (t, J = 7.9 Hz, 1H), 7.21 – 7.14 (m, 1H), 7.14 – 7.09 (m, 1H), 6.88 (ddd, J = 8.2, 2.6, 0.9 Hz, 1H), 3.86 (s, 3H), 1.36 (s, 9H). ^{13}C NMR (101 MHz, CDCl$_3$) δ 159.86, 150.40, 142.59, 138.16, 129.65, 126.80, 125.66, 119.54, 112.72, 112.37, 55.23, 34.52, 31.34.

4'-(*tert*-Butyl)-[1,1'-biphenyl]-3-amine (4b) [14]

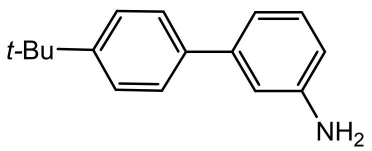

White solid, m. p. 146-148℃. ^{1}H NMR (300 MHz, CDCl$_3$) δ 7.54 – 7.40 (m, 4H), 7.21 (t, J = 7.8 Hz, 1H), 7.03 – 6.96 (m, 1H), 6.91 (t, J = 1.9 Hz, 1H), 6.71 – 6.61 (m, 1H), 1.35 (s, 9H). ^{13}C NMR (101 MHz, CDCl$_3$) δ 150.09, 146.60, 142.16, 138.37, 129.53, 126.64, 125.51, 117.47, 113.81, 113.70, 34.43, 31.31.

4-(*tert*-Butyl)-4'-(trifluoromethyl)-1,1'-biphenyl (4c) [15]

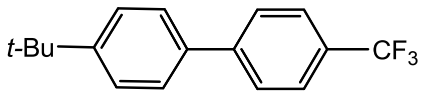

White solid, m. p. 133-134℃. ^{1}H NMR (300 MHz, $CDCl_3$) δ 7.68 (s, 4H), 7.58 – 7.46 (m, 4H), 1.37 (s, 9H). ^{13}C NMR (101 MHz, $CDCl_3$) δ 151.35, 144.53, 136.81, 127.19, 126.91, 125.95, 125.66, 125.62, 34.62, 31.30.

4'-(*tert*-Butyl)-[1,1'-biphenyl]-3-carbonitrile (4d) [14]

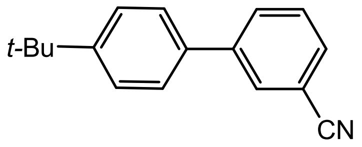

White solid, m. p. 103-105℃. ^{1}H NMR (300 MHz, $CDCl_3$) δ 7.85 (dd, J = 2.3, 1.1 Hz, 1H), 7.83 – 7.77 (m, 1H), 7.60 (dt, J = 7.7, 1.4 Hz, 1H), 7.53 (d, J = 7.7 Hz, 1H), 7.50 (s, 4H), 1.37 (s, 9H). ^{13}C NMR (101 MHz, $CDCl_3$) δ 151.45, 142.09, 135.75, 131.14, 130.32, 130.28, 129.42, 126.59, 125.97, 118.81, 112.74, 34.50, 31.17.

4'-(*tert*-Butyl)-3-nitro-1,1'-biphenyl (4e) [16]

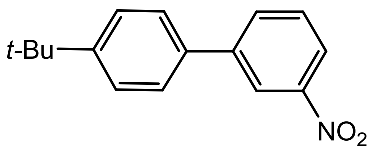

White solid, m. p. 111-112℃. ^{1}H NMR (300 MHz, $CDCl_3$) δ 8.45 (t, J = 1.9 Hz, 1H), 8.18 (ddd, J = 8.2, 2.3, 1.0 Hz, 1H), 7.91 (ddd, J = 7.8, 1.7, 1.0 Hz, 1H), 7.65 – 7.48 (m, 5H), 1.38 (s, 9H).^{13}C NMR (101 MHz,) δ 151.69, 148.64, 142.62, 135.63, 132.74, 129.56, 126.73, 126.06, 121.65, 121.63, 34.59, 31.22.

3-[4-(*tert*-Butyl)phenyl]pyridine (4f) [17]

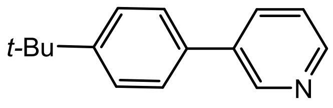

White solid, m. p. 87-88℃. ^{1}H NMR (300 MHz, $CDCl_3$) δ 8.85 (d, J = 1.7 Hz, 1H), 8.57 (dd, J = 4.8, 1.6 Hz, 1H), 7.88 (ddd, J = 7.9, 2.3, 1.7 Hz, 1H), 7.52 (d, J = 1.9 Hz, 4H), 7.36 (ddd, J = 7.9, 4.8, 0.8 Hz, 1H), 1.37 (s, 9H). ^{13}C NMR (101 MHz, $CDCl_3$) δ 151.20, 148.12, 148.07, 136.43, 134.81, 134.14, 126.74, 126.00, 123.47, 34.56, 31.20.

6-[4-(*tert*-Butyl)phenyl]-1*H*-indole (4g) [18]

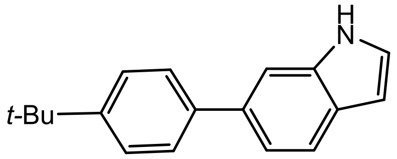

White solid, m. p. 74-76℃. ^{1}H NMR (300 MHz, $CDCl_3$) δ 8.15 (brs, 1H), 7.68 (d, J = 8.3 Hz, 1H), 7.65 – 7.54 (m, 3H), 7.51 – 7.43 (m, 2H), 7.39 (dd, J = 8.2, 1.6 Hz, 1H), 7.21 (dd, J = 3.2, 2.5 Hz, 1H), 6.56 (ddd, J = 3.1, 2.0, 0.9 Hz, 1H), 1.37 (s, 9H). ^{13}C NMR (101 MHz, $CDCl_3$) δ 149.52, 139.32, 136.33, 135.34, 126.95, 125.63, 124.66, 120.78, 119.70, 109.32, 102.41, 34.46, 31.39.

4-[4-(*tert*-Butyl)phenyl]-1-methyl-1*H*-pyrazole (4h) [19]

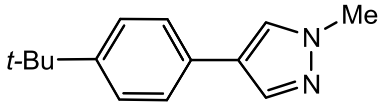

White solid, m. p. 68-69℃. ^{1}H NMR (300 MHz, $CDCl_3$) δ 7.74 (s, 1H), 7.57 (s, 1H), 7.40 (d, J = 1.1 Hz, 4H), 3.94 (s, 3H), 1.33 (s, 9H). ^{13}C NMR (101 MHz, $CDCl_3$) δ 149.20, 136.59, 129.69, 126.62, 125.64, 125.16, 123.02, 38.92, 34.38, 31.25.

4'-Methyl-[1,1'-biphenyl]-4-ol (6a) [20] (Table 6, entry 1)

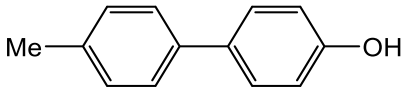

White solid, m. p. 153-154℃. ^{1}H NMR (400 MHz, $CDCl_3$) δ 7.45 (t, J = 8.7 Hz, 4H), 7.23 (d, J = 7.9 Hz, 2H), 6.90 (d, J = 8.6 Hz, 2H), 4.96 (s, 1H), 2.39 (s, 3H). ^{13}C NMR (101 MHz, $CDCl_3$) δ 154.77, 137.80, 136.39, 133.86, 129.41, 128.15, 126.52, 115.52, 21.06.

3-Methoxyl-4'-(trifluoromethyl)-1,1'-biphenyl (6b) [21] (Table 6, entry 2)

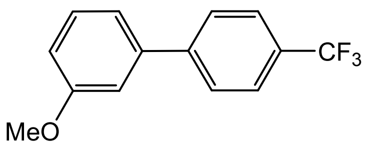

White solid, m. p. 156-158℃. ^{1}H NMR (300 MHz, $CDCl_3$) δ 7.68 (s, 4H), 7.39 (t, J = 7.9 Hz, 1H), 7.18 (ddd, J = 7.6, 1.6, 1.0 Hz, 1H), 7.14 – 7.10 (m, 1H), 6.95 (ddd, J = 8.2, 2.6, 0.9 Hz, 1H), 3.87 (s, 3H).^{13}C NMR (101 MHz, $CDCl_3$) δ 160.06, 144.56, 141.20, 129.99, 127.42, 125.62, 125.68, 125.59, 119.69, 113.41, 113.10, 55.25.

Methyl 3'-formyl-[1,1'-biphenyl]-3-carboxylate (6c) [22] (Table 6, entry 3)

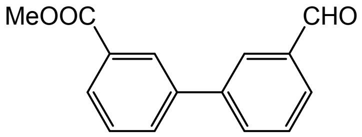

White solid, m. p. 101-103℃. ^{1}H NMR (400 MHz, $CDCl_3$) δ 10.03 (s, 1H), 8.07 (dd, J = 5.8, 4.0 Hz, 3H), 7.83 (ddd, J = 7.9, 4.8, 1.4 Hz, 2H), 7.65 – 7.61 (m, 2H), 7.57 (t, J = 7.7 Hz, 1H), 3.88 (s, 3H). ^{13}C NMR (101 MHz, $CDCl_3$) δ 192.05, 166.75, 143.98, 140.93, 136.95, 133.08, 130.27, 129.66, 129.58, 129.49, 128.14, 127.08, 52.22.

3'-Nitro-[1,1'-biphenyl]-3-amine (6d) [23] (Table 6, entry 4)

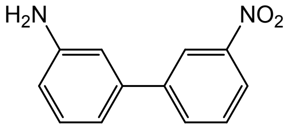

White solid, m. p. 224-225℃. ^{1}H NMR (300 MHz, $CDCl_3$) δ 8.42 (t, J = 2.0 Hz, 1H), 8.18 (ddd, J = 8.2, 2.3, 1.0 Hz, 1H), 7.88 (ddd, J = 7.7, 1.7, 1.1 Hz, 1H), 7.58 (t, J = 8.0 Hz, 1H), 7.26 (t, J = 3.9 Hz, 1H), 7.03 – 6.97 (m, 1H), 6.92 (t, J = 2.0 Hz, 1H), 6.75 (ddd, J = 8.0, 2.3, 0.8 Hz, 1H), 3.82 (brs, 2H). ^{13}C NMR (101 MHz, $CDCl_3$) δ 148.57, 147.08, 143.02, 139.77, 132.96, 130.07, 129.52, 121.91, 121.84, 117.36, 115.13, 113.53.

2-(Naphthalen-1-yl)quinoline (6e) [24] (Table 6, entry 5)

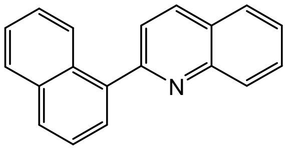

White solid, m. p. 95-97℃. ^{1}H NMR (400 MHz, $CDCl_3$) δ 8.21 (t, J = 8.9 Hz, 2H), 8.13 (d, J = 8.3 Hz, 1H), 7.91 (t, J = 7.6 Hz, 2H), 7.84 (d, J = 8.2 Hz, 1H), 7.77 – 7.71 (m, 1H), 7.71 – 7.68 (m, 1H), 7.65 (d, J = 8.4 Hz, 1H), 7.55 (dd, J = 14.8, 7.4 Hz, 2H), 7.50 – 7.41 (m, 2H). ^{13}C NMR (101 MHz, $CDCl_3$) δ 159.29, 147.99, 138.60, 136.15, 133.88, 131.13, 129.63, 129.59, 129.01, 128.30, 127.65, 127.48, 126.86, 126.48, 125.86, 125.58, 125.28, 123.14.

6-(6-Fluoropyridin-3-yl)-1*H*-indole (6f) [25] (Table 6, entry 6)

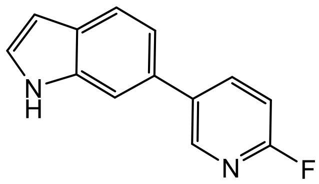

White solid, m. p. 69-71℃. ^{1}H NMR (300 MHz, $CDCl_3$) δ 8.47 (dd, J = 1.6, 0.7 Hz, 1H), 8.40 (brs, 1H), 8.02 (ddd, J = 8.4, 7.7, 2.6 Hz, 1H), 7.73 (d, J = 8.2 Hz, 1H), 7.56 (d, J = 0.7 Hz, 1H), 7.33 – 7.27 (m, 2H), 7.00 (ddd, J = 8.5, 3.0, 0.6 Hz, 1H), 6.60 (ddd, J = 3.0, 2.0, 0.9 Hz, 1H). ^{13}C NMR (101 MHz, $CDCl_3$) δ 163.93, 145.75, 139.88, 136.26, 135.97, 130.70, 127.79, 125.39, 121.41, 119.33, 109.51, 109.09, 102.65.

1-Methyl-4-(thiophen-2-yl)-1*H*-pyrazole (6g) [24] (Table 6, entry 7)

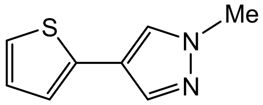

White solid, m. p. 64-65℃. ^{1}H NMR (300 MHz, $CDCl_3$) δ 7.66 (s, 1H), 7.54 (s, 1H), 7.16 (dd, J = 5.0, 1.2 Hz, 1H), 7.03 (ddd, J = 8.5, 4.2, 2.4 Hz, 2H), 3.92 (s, 3H). ^{13}C NMR (101 MHz, $CDCl_3$) δ 136.93, 135.00, 127.56, 126.99, 122.90, 122.37, 117.08, 39.03.

Reference

1 P. B. Dzhevakov, M. A. Topchiy, D. A. Zharkova, *Adv. Syn. Cat.*, 2016, **358**, 977-983.

2 G. H. Zeng, R. S. Zhang, W. M. Wang, W. Wang, F. S. Liang. *ACS. Chem. Bio.*, 2015, **10**, 1404-1410.

3 G. A. Moland, S. L. Trice, S. M. Kennedy, *J. Org. Chem.*, 2012, **77**, 8678−8688.

4 R. D. Hubbard, N. Y. Bamaung, S. D. Fidanze, S. A. Erickson, F. Palazzo, J. L.Wilsbacher, Q. Zhang, L. A.Tucker, X. M. Hu, P. Kovar, D. J. Osterling, E. F. Johnson, J. Bouska, J. Y. Wang, S. K. Davidsen, R. L. Bell, G. S. Sheppard, *Bioorg. Med. Chem. Lett.*, 2009, **19**, 1718-1721.

5 K. L. Billingsley, T. E. Barder, S. L. Buchwald, *Angew. Chem. Int. Ed.*, 2007, **46**, 5359-5363.

6 M. A. Larsen, J. F. Hartwig, *J. Am. Chem. Soc.,* 2014, **136**, 4287-4299.

7 J. O. Bauer, S. Chakraborty, D. Milstein, *ACS Catal.*, 2017, **7**, 4462-4466.

8 M. Dabiri, M. Shariatipour, S. K. Movahed, *RSC Adv.*, 2014, **4**, 39428-39434.

9 G. A. Molander, C. R. Bernardi, *J. Org. Chem.*, 2002 , **33**, 8424-8429.

10 B. Schmidt, M. Riemer, *J. Org. Chem.*, 2014, **79**, 4104-4118.

11 Y. Yuan, Y. Bian, *Appl. Organomet. Chem.*, 2008, **22**, 15-18.

12 R. N. Dhital, C. Kamonsatikul, E. Somsook, H. Sakurai. *Catal. Sci. Technol.*, 2013, **3**, 3030-3035.

13 Q. Chen, Z. Q. Mao, F. Guo, X. H. Liu, *Tetrahedron Lett.*, 2016, **57**, 3735-3738.

14 T. Nguyen, N. German, A. M. Decker, T. L. Langston, T. F. Gamage, C. E. Farquhar, J. X. Li, J. L. Wiley, B. F. Thomas, Y. Zhang, *J. Med. Chem.*, 2017, **60**, 7410-7424.

15 H. Ren, Y. Xu, E. Jeanneau, *Tetrahedron*, 2014, **70**, 2829-2837.

16 T. Nguyen, N. German, A. M. Decker, *J. Med. Chem.*, 2017, **60**, 7410-7424.

17 W. Gati, M. M. Rammah, M. B. Rammah, F. Couty, G. Evano, *J. Am. Chem. Soc.*, 2012, **134**, 9078-9081.

18 M. Prieto, E. Zurita, E. Rosa, L. Muñoz, P. Lloyd-Williams, E. Giralt, *J .Org. Chem.*, 2004, **69**, 6812-6820.

19 H. Cheng, Q. Y. Wu, F. Han, G. F. Yang, *Chinese Chem. Lett.*, 2014, **25**, 705-709.

20 G. A. Edwards, M. A. Trafford, A. E. Hamilton, A. M. Buxton, M. C. Bardeaux, J. M. Chalker. *J. Org. Chem.*, 2014, **79**(5), 2094-2104.

21 L. Zhang, J. Wu, *J. Am. Chem. Soc.*, 2008, **130**, 12250-12251.

22 B. Bhayana, B. P. Fors, S. L. Buchwald, *Org. Lett.*, 2009, **11**, 3954-3957.

23 T. Nguyen, N. German, A. M. Decker, *J. Med. Chem.*, 2017, **60**, 7410-7424.

24 A. M. Berman, R. G. Bergman, J. A. Ellman. *J. Org. Chem.*, 2010, **75**, 7863-7868.

25 H. Xiong, A. T. Hoye, K. H. Fan, X. M. Li, J. Clemens, C. L. Horchler, N. C. Lim, G. Attardo, *Org. Lett.*, 2015, **17**, 3726-3729.

Copies of the ^1H and ^{13}C NMR spectra of the products

^1H and ^{13}C NMR spectra of compound 2a

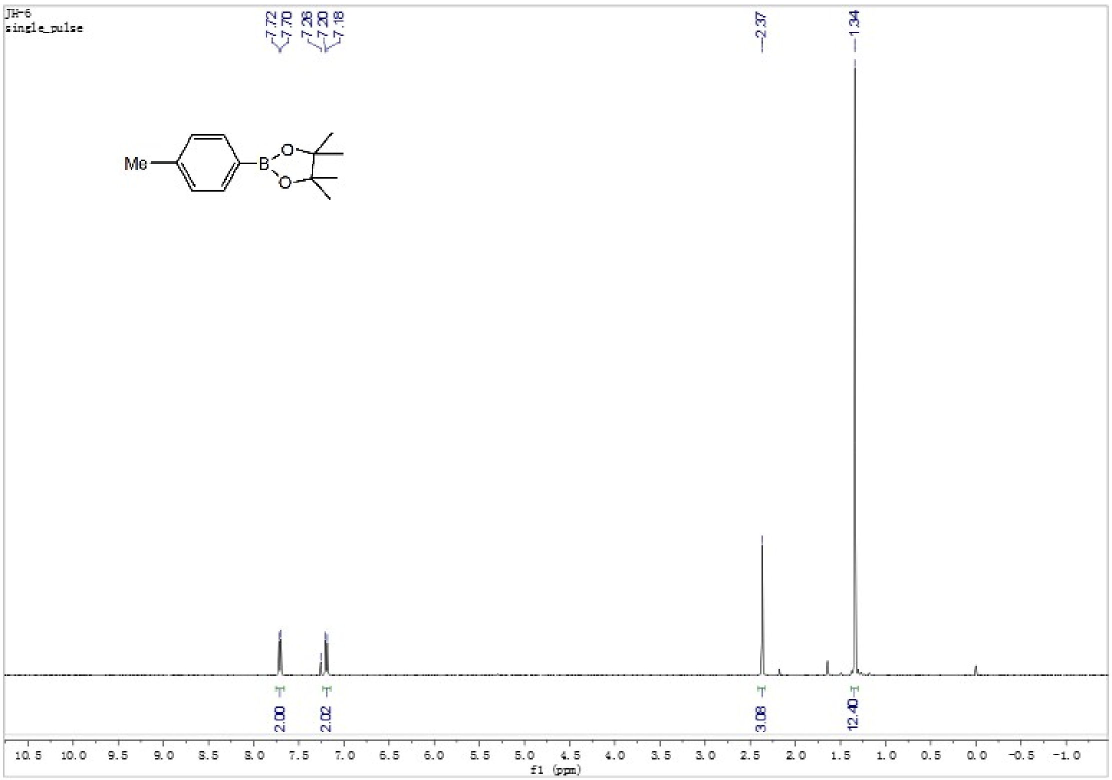

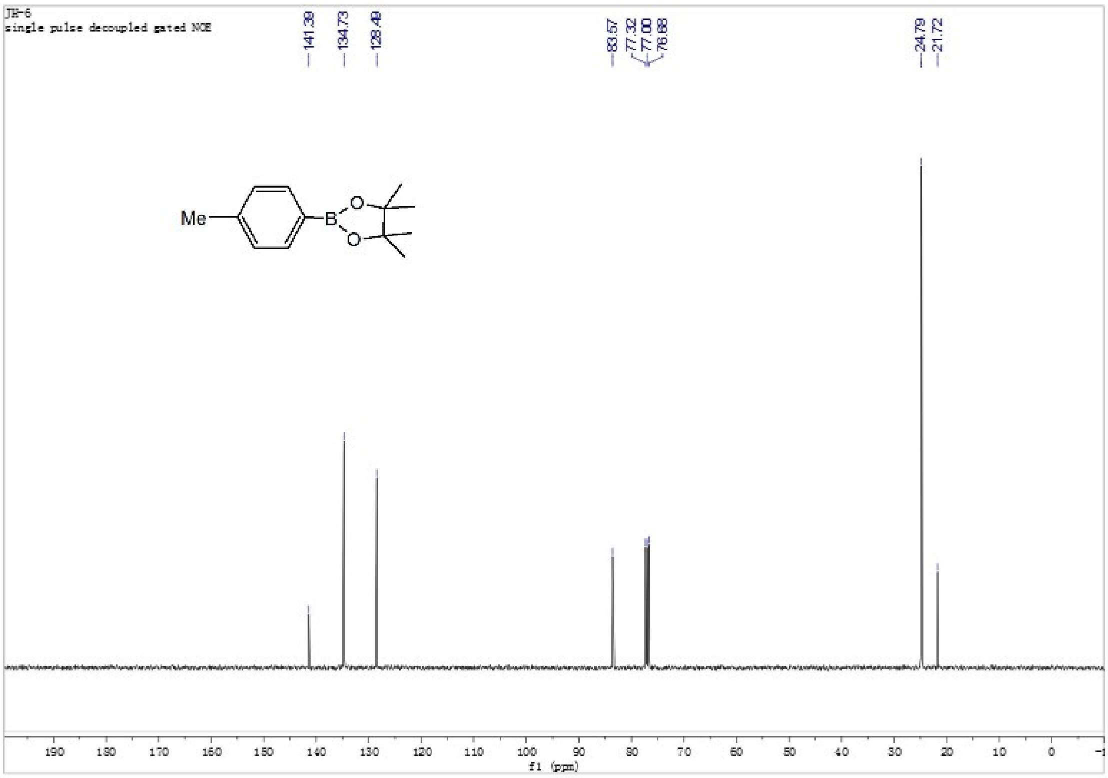

^{1}H and ^{13}C NMR spectra of compound 2b

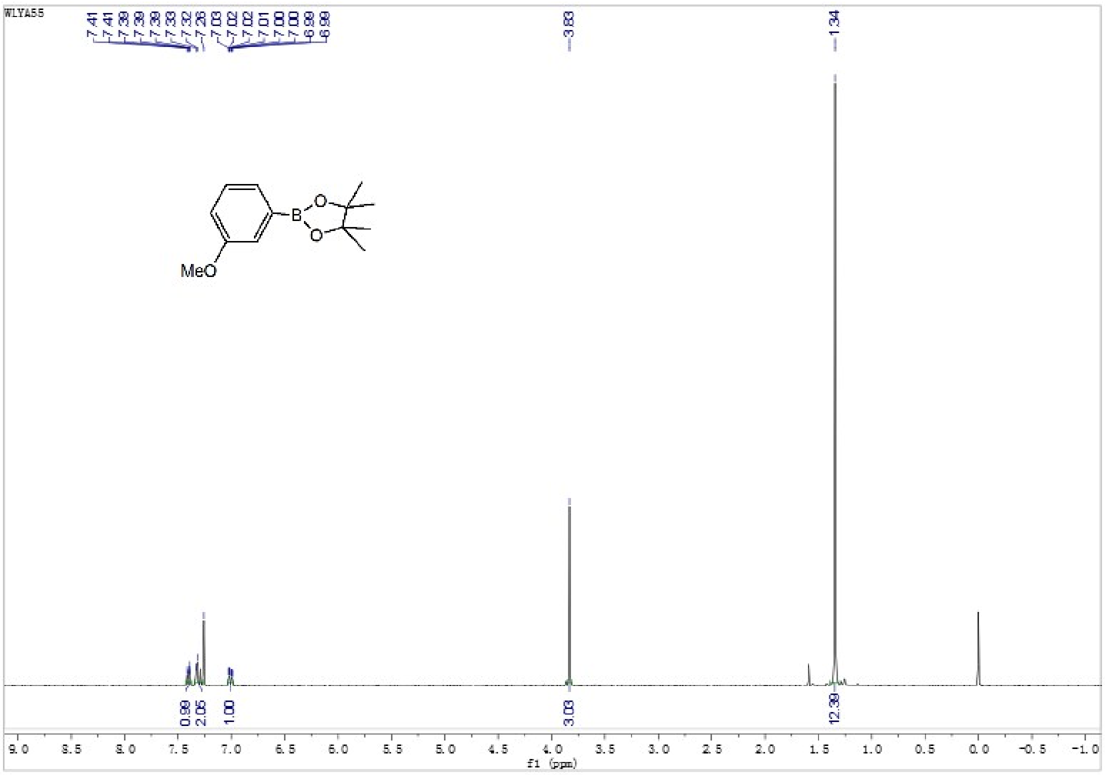

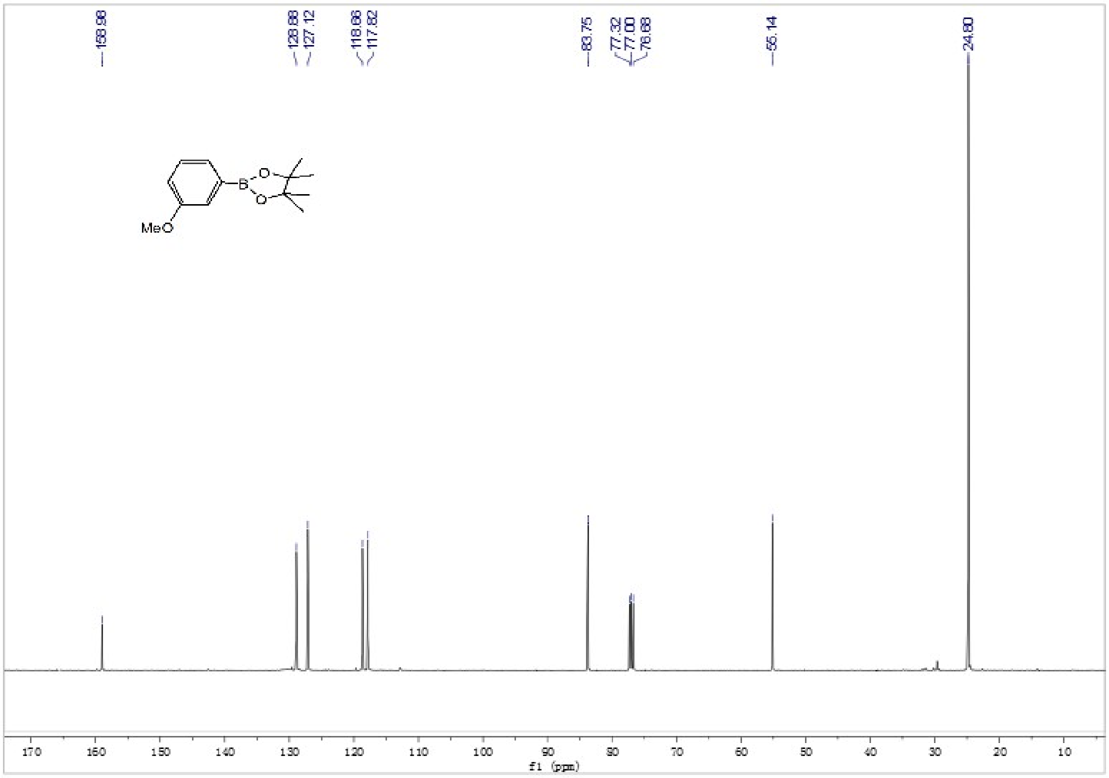

^{1}H and ^{13}C NMR spectra of compound 2c

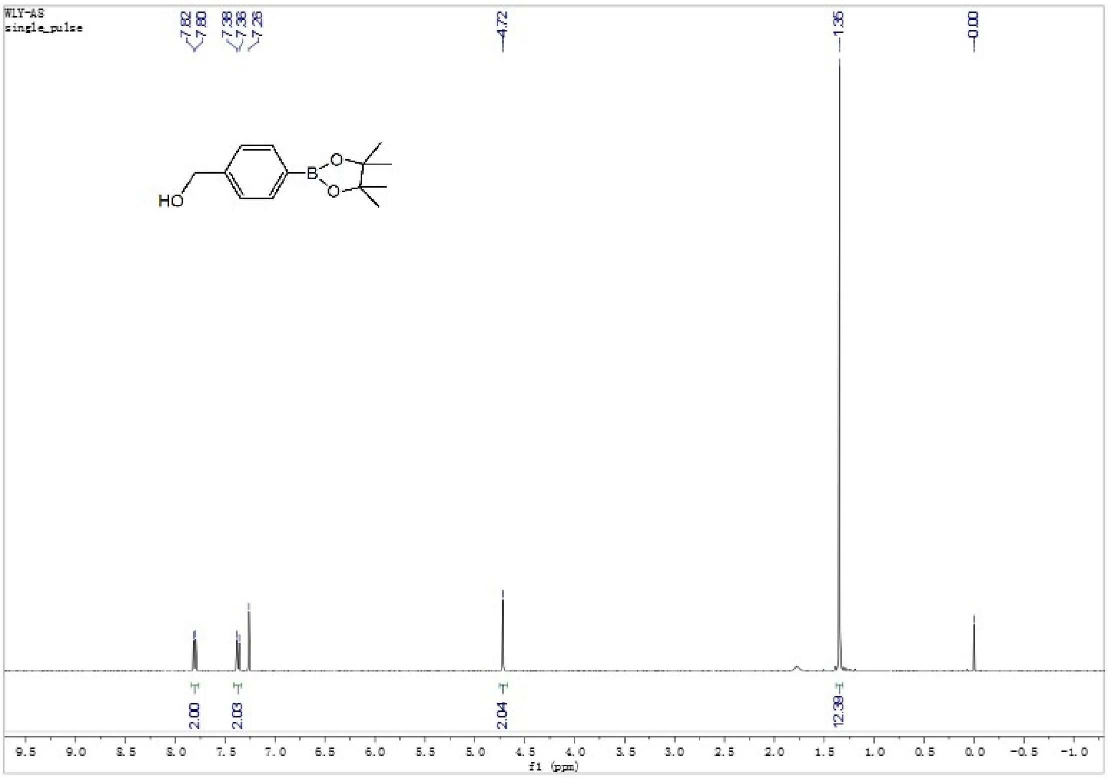

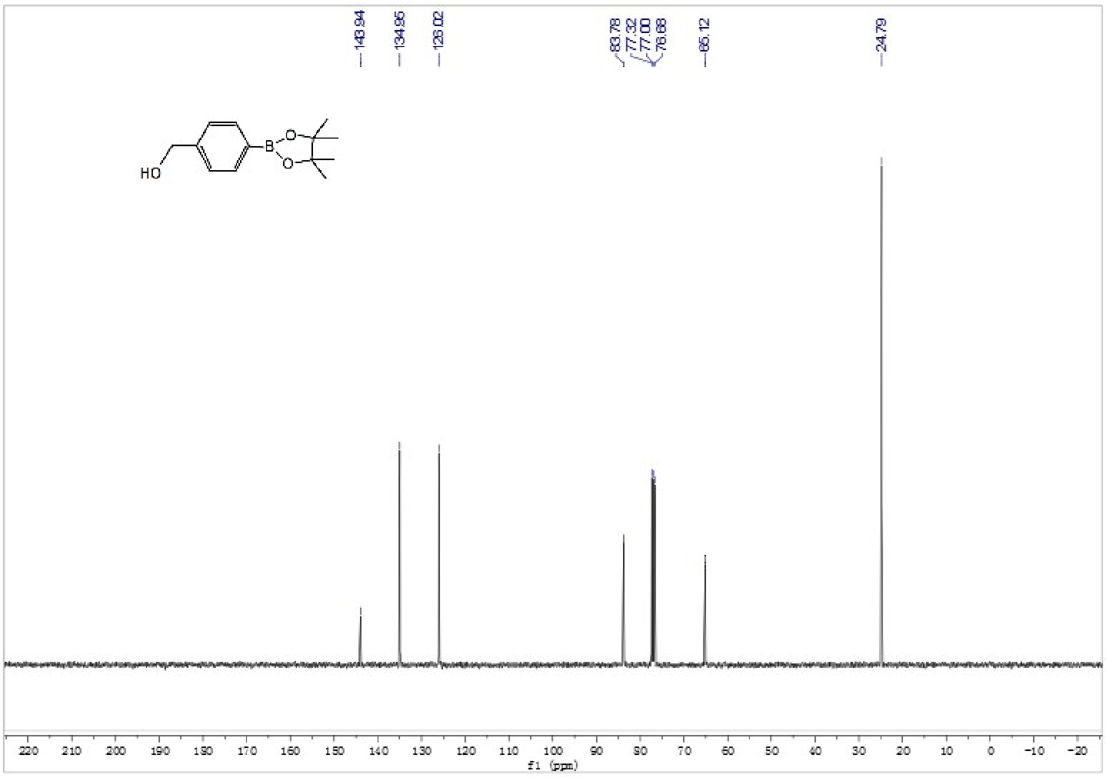

^{1}H and ^{13}C NMR spectra of compound 2d

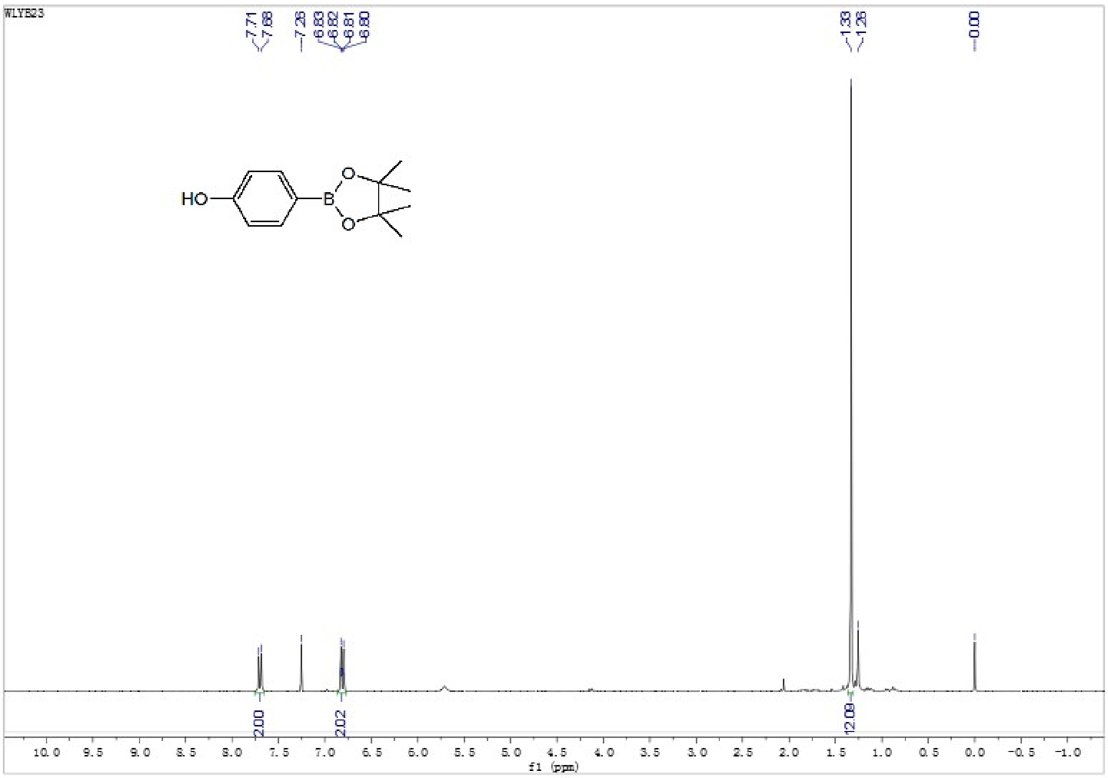

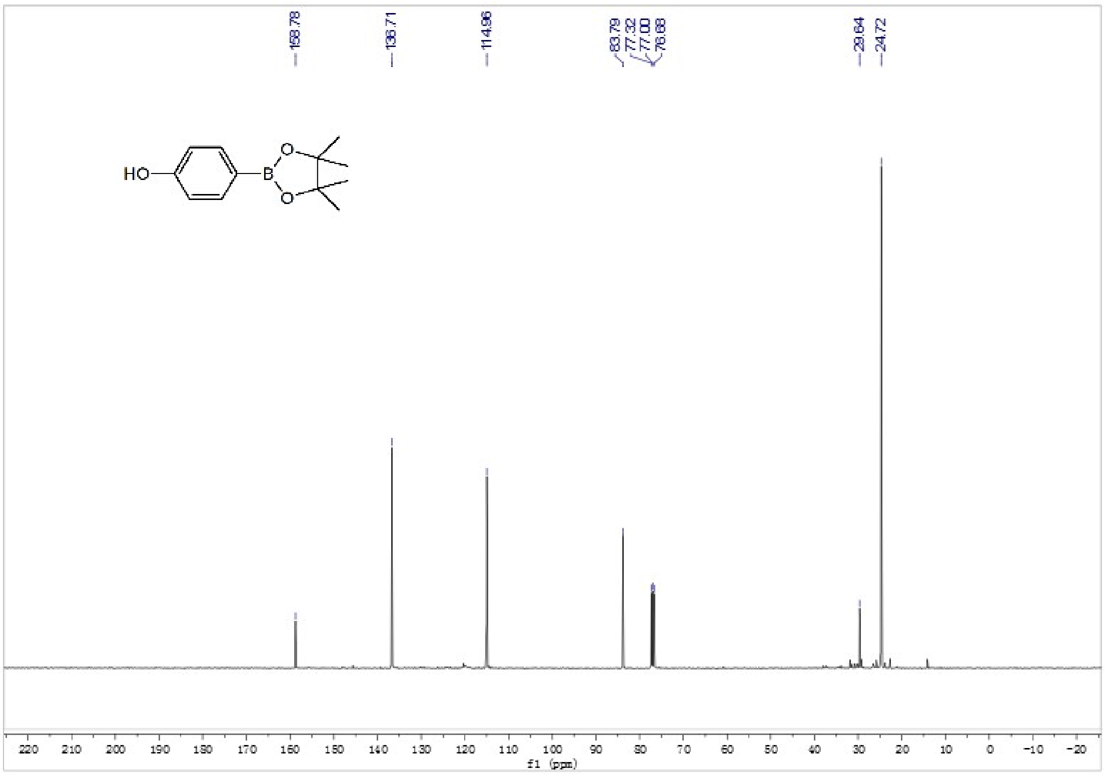

^{1}H and ^{13}C NMR spectra of compound 2e

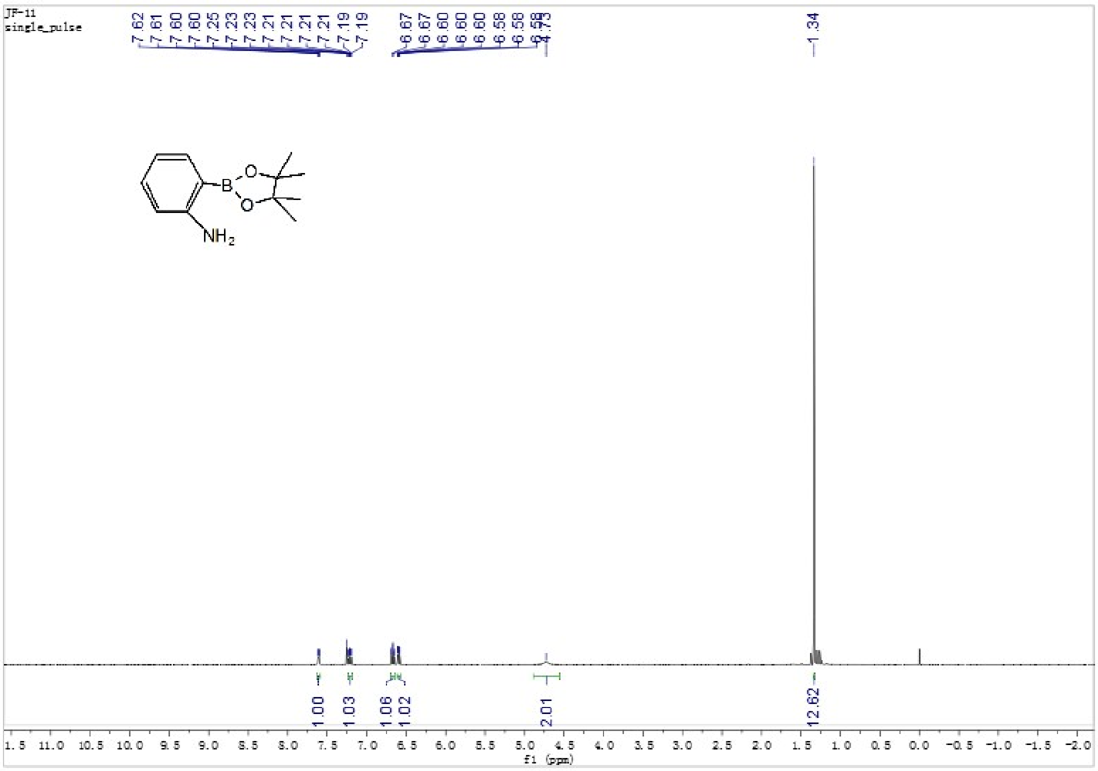

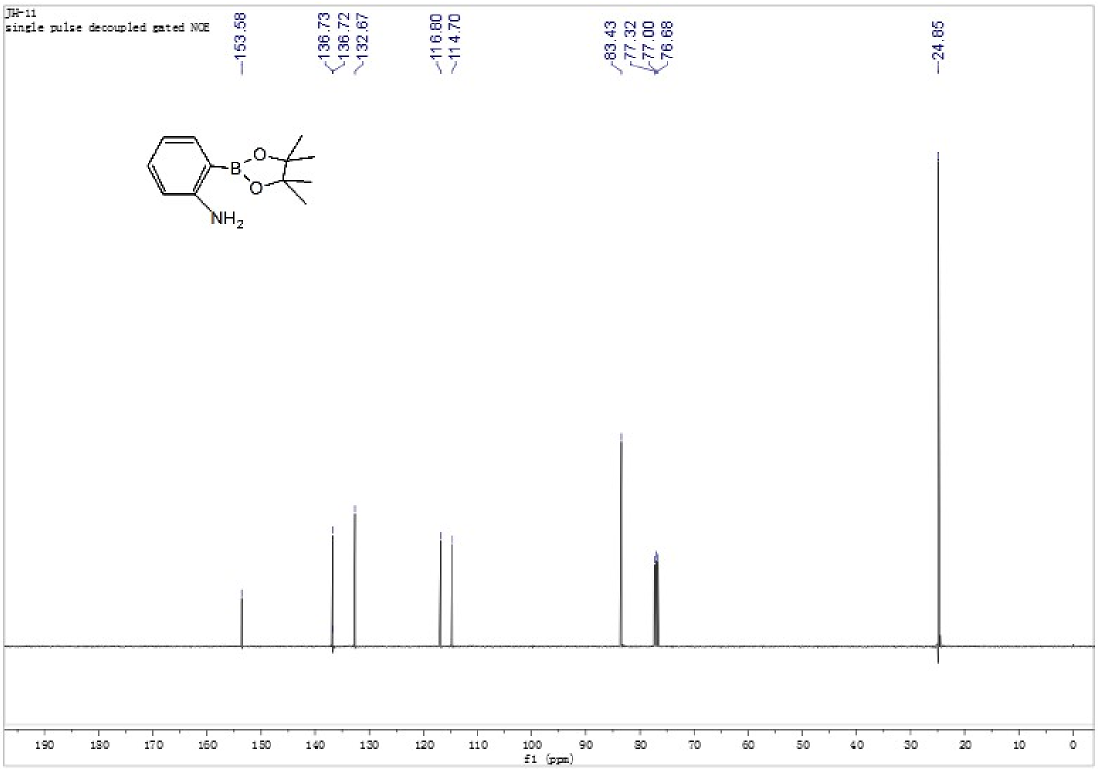

^{1}H and ^{13}C NMR spectra of compound 2f

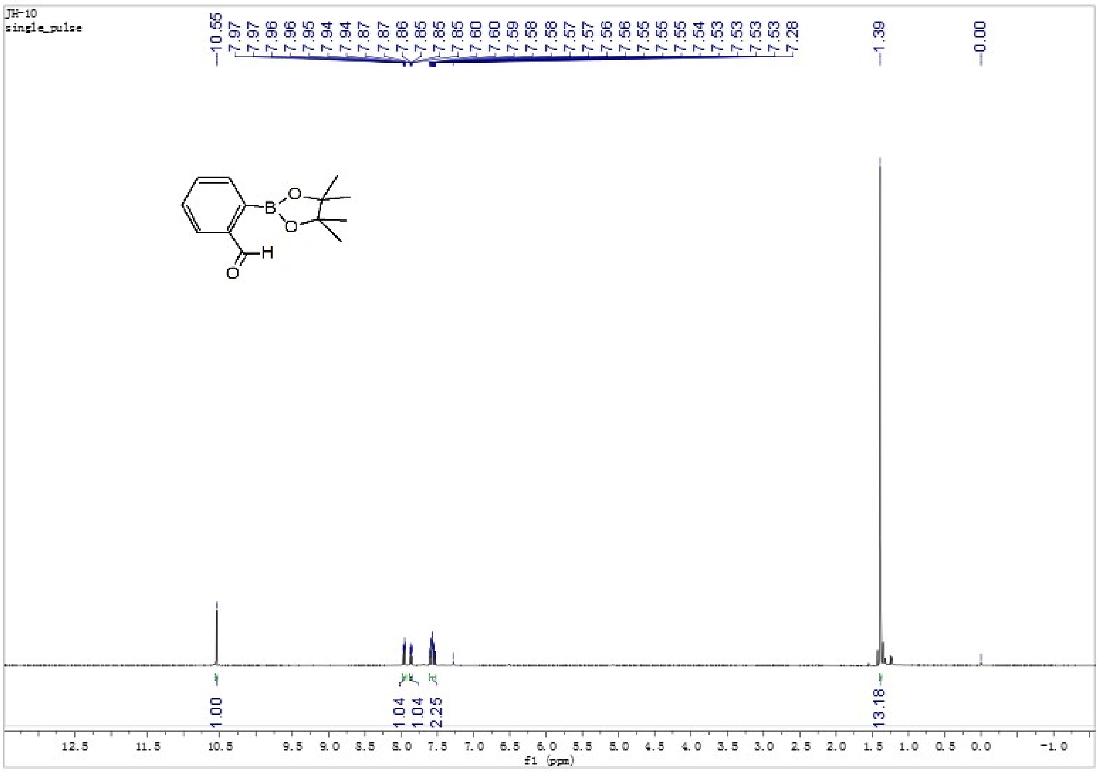

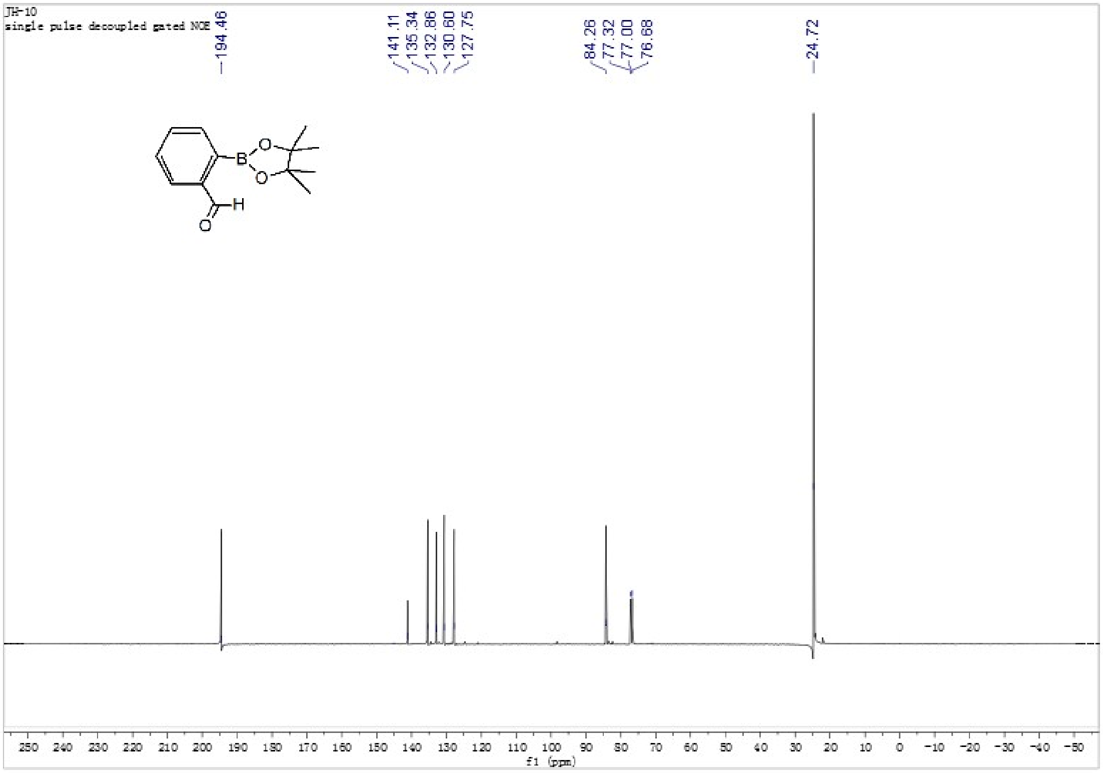

^{1}H and ^{13}C NMR spectra of compound 2g

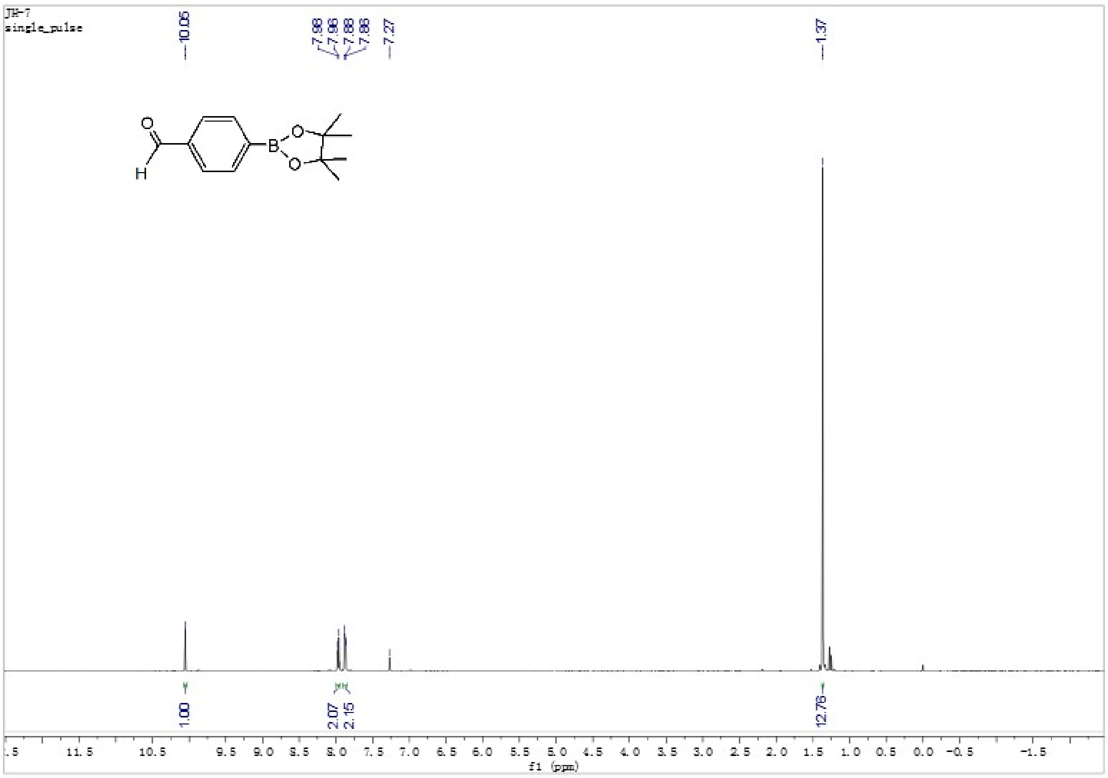

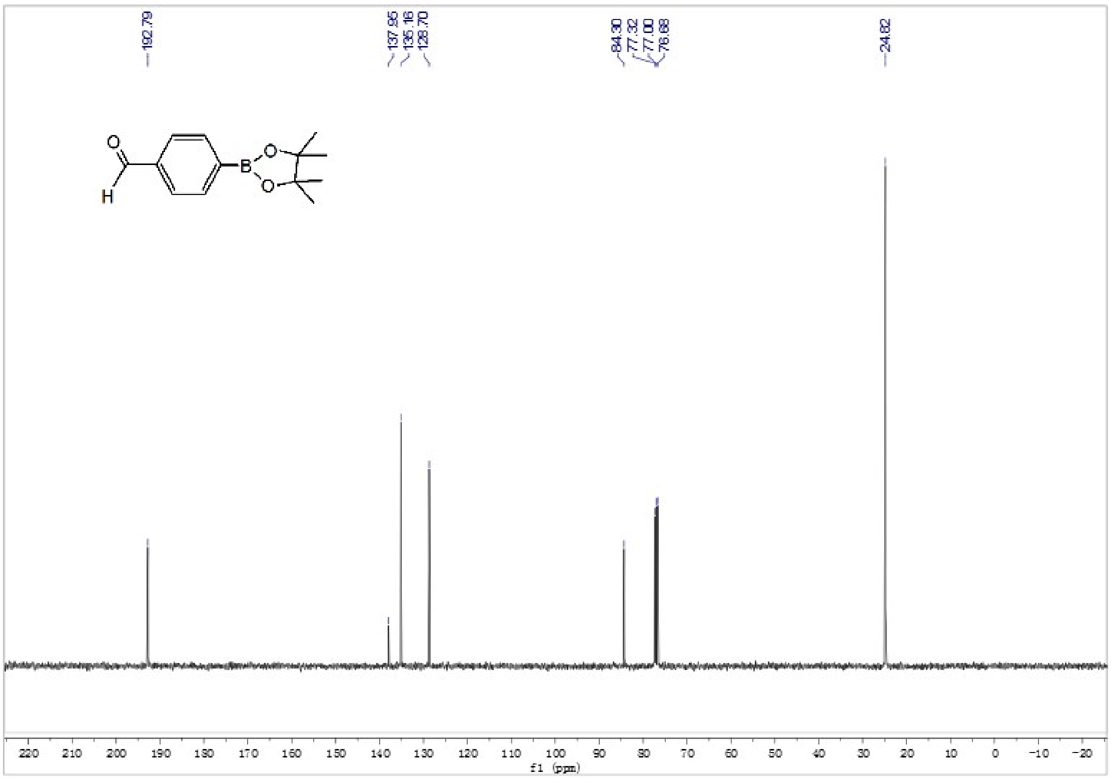

^{1}H and ^{13}C NMR spectra of compound 2h

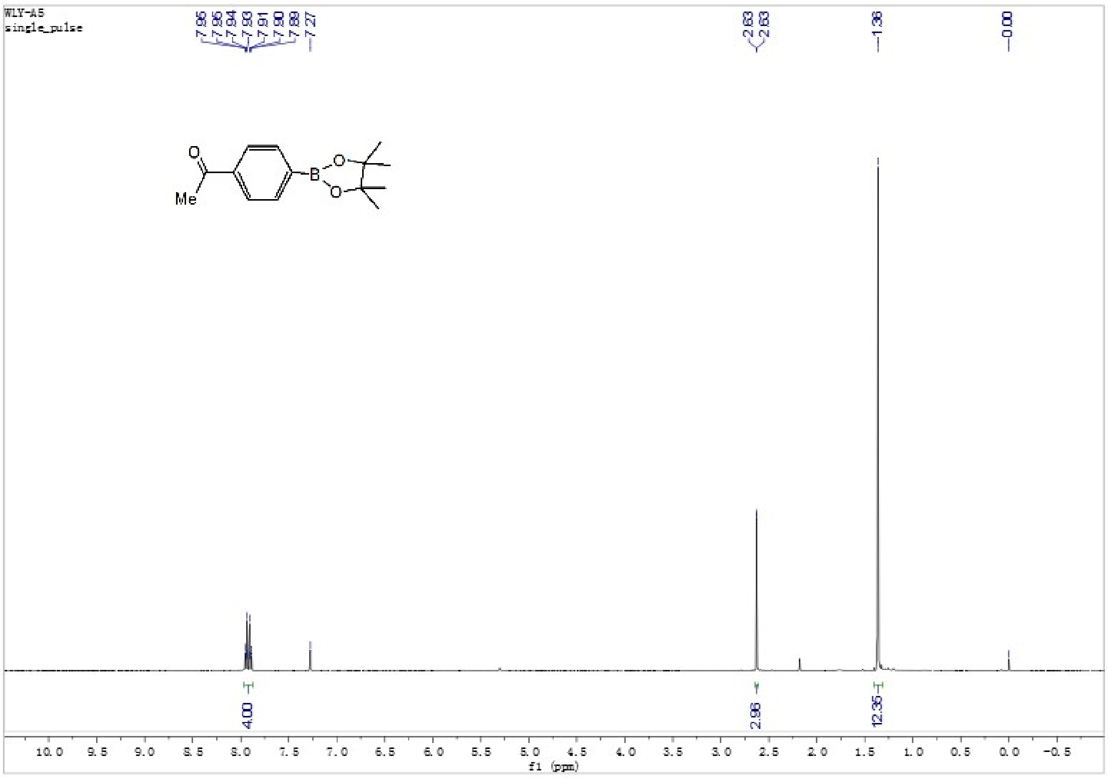

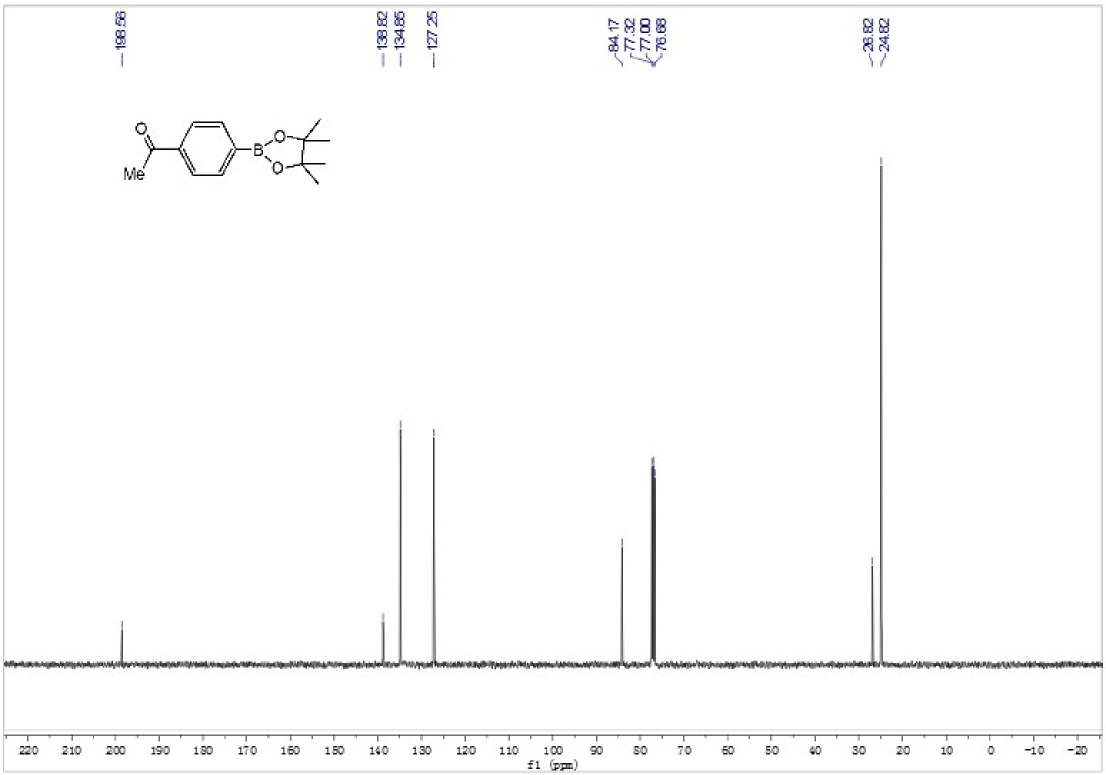

^{1}H and ^{13}C NMR spectra of compound 2i

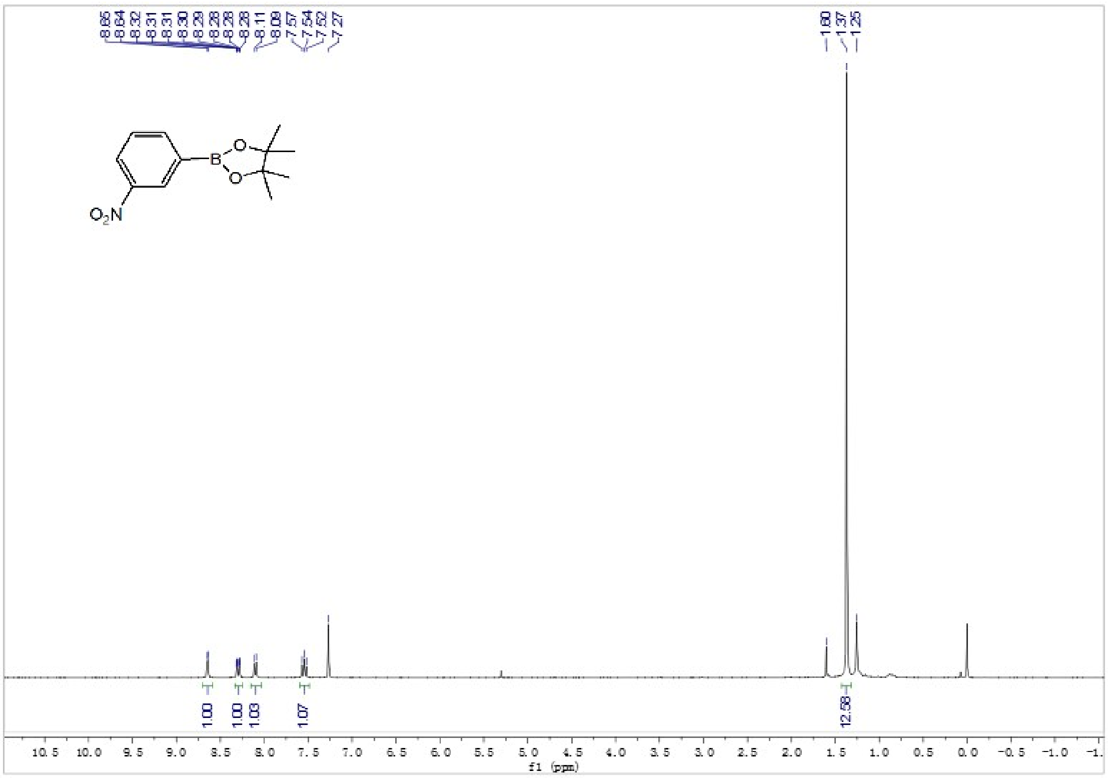

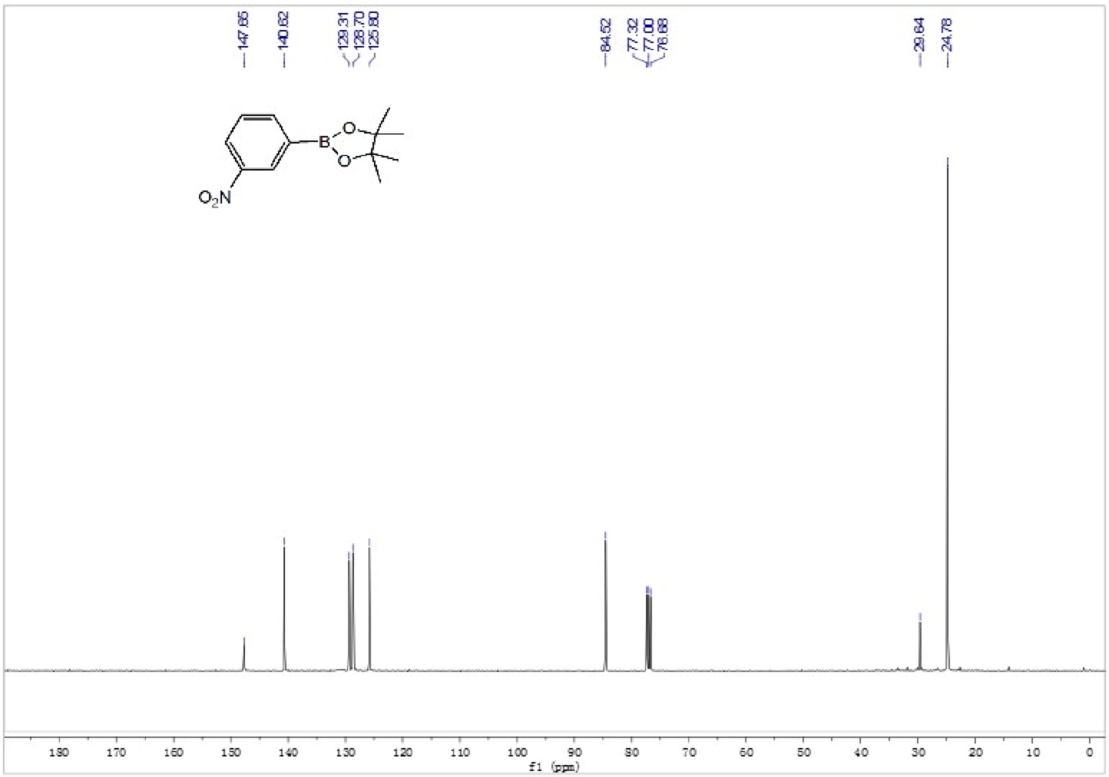

^{1}H and ^{13}C NMR spectra of compound 2j

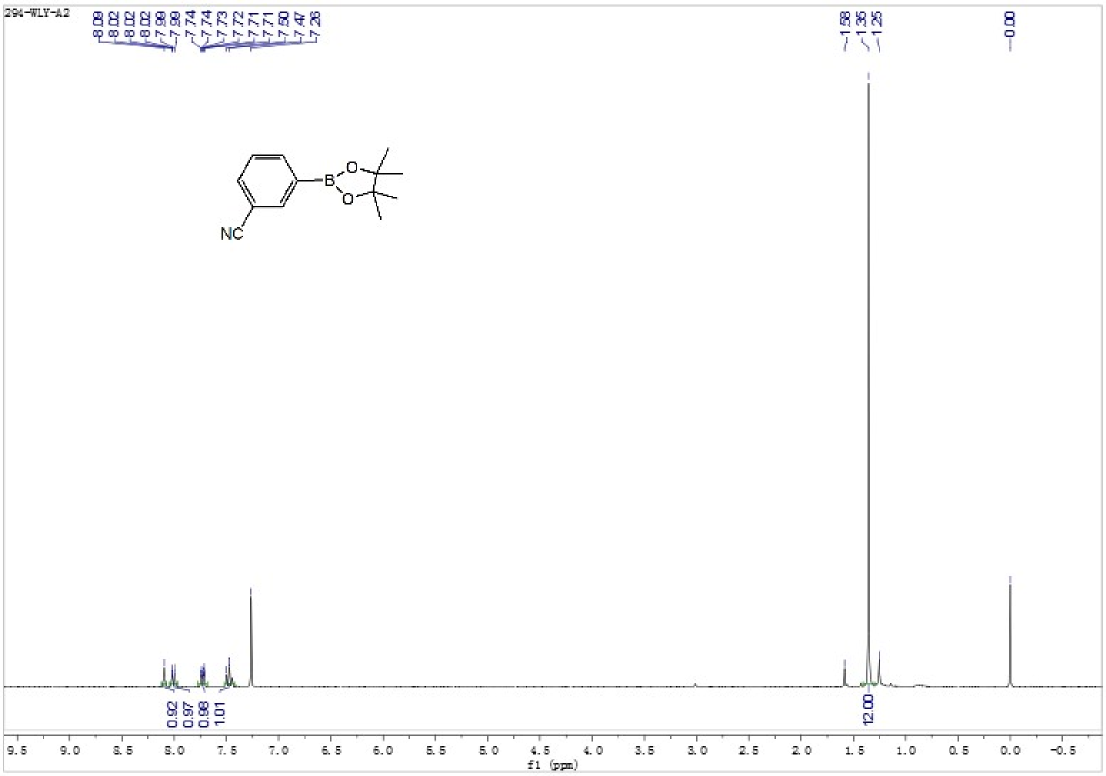

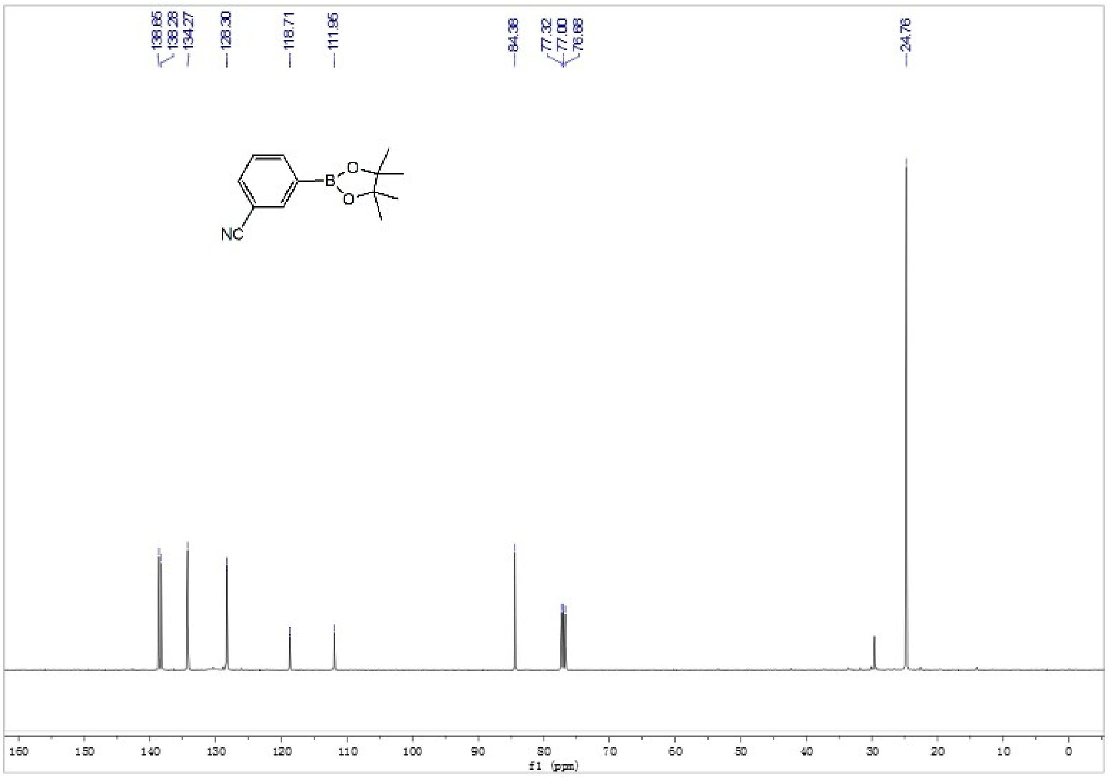

^{1}H and ^{13}C NMR spectra of compound 2k

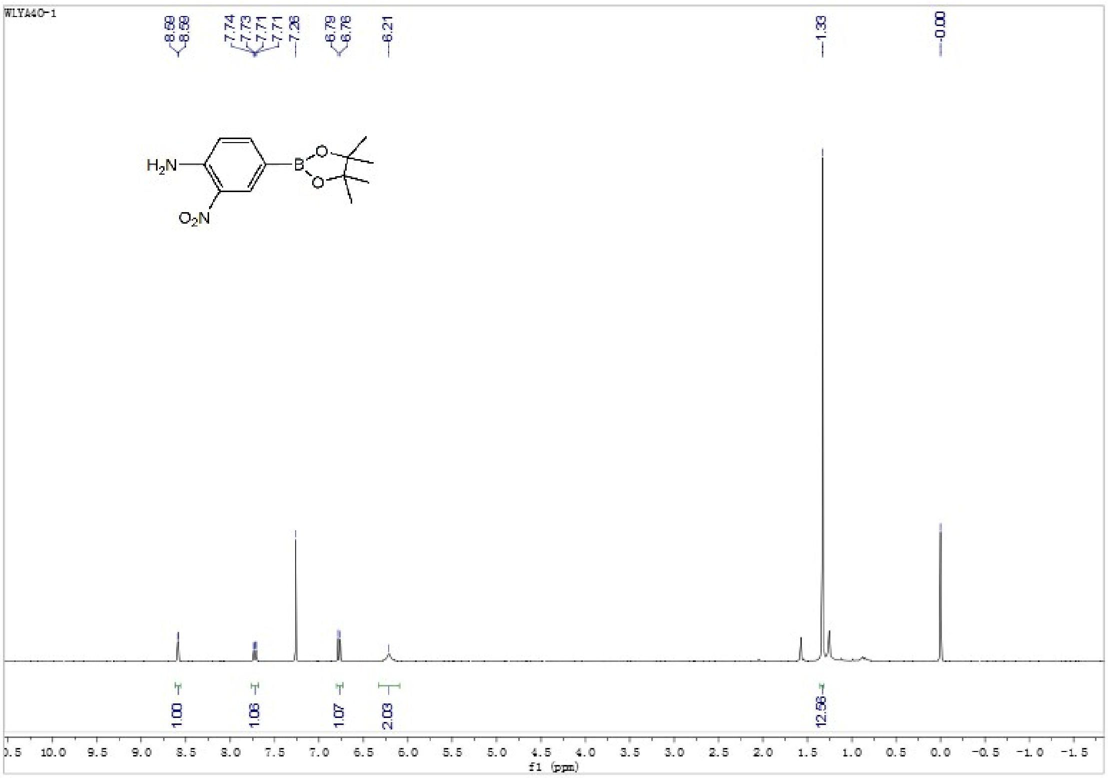

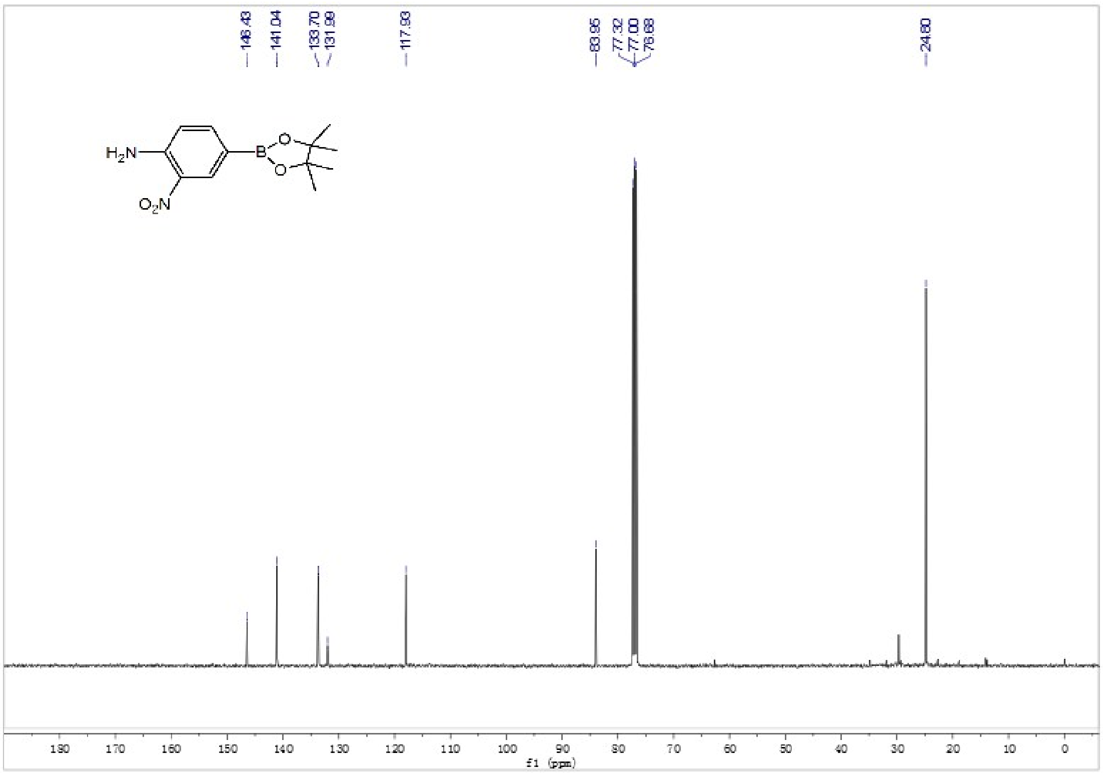

^{1}H and ^{13}C NMR spectra of compound 2l

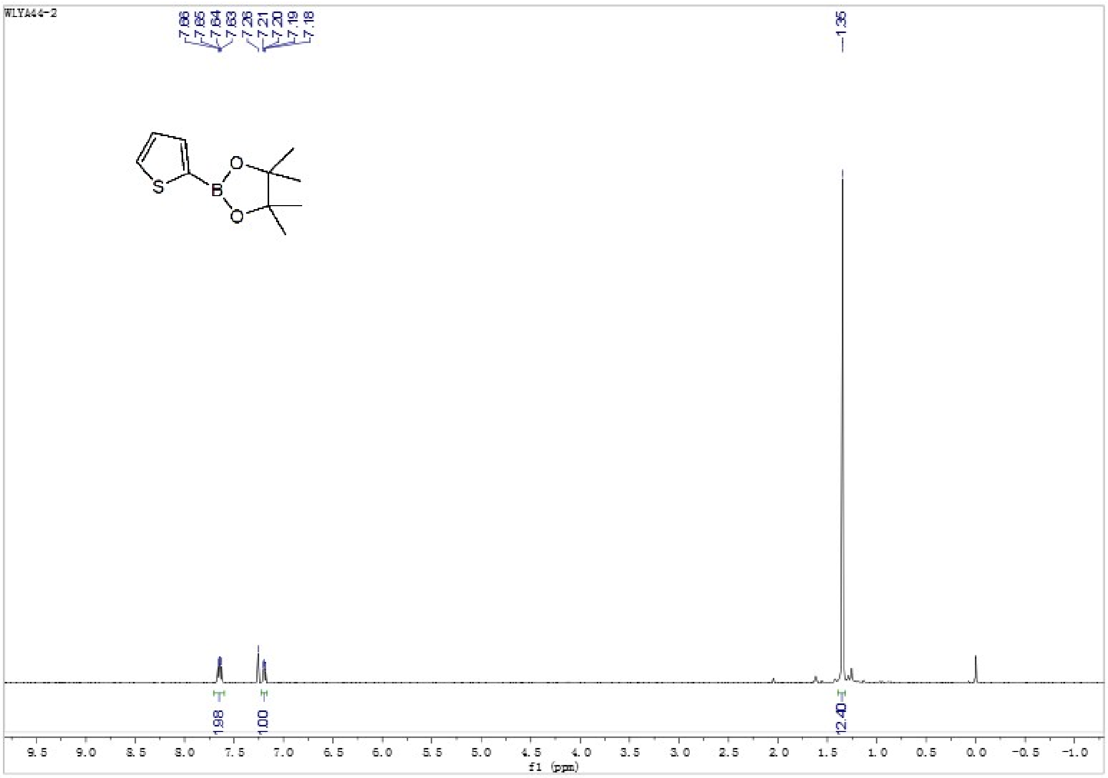

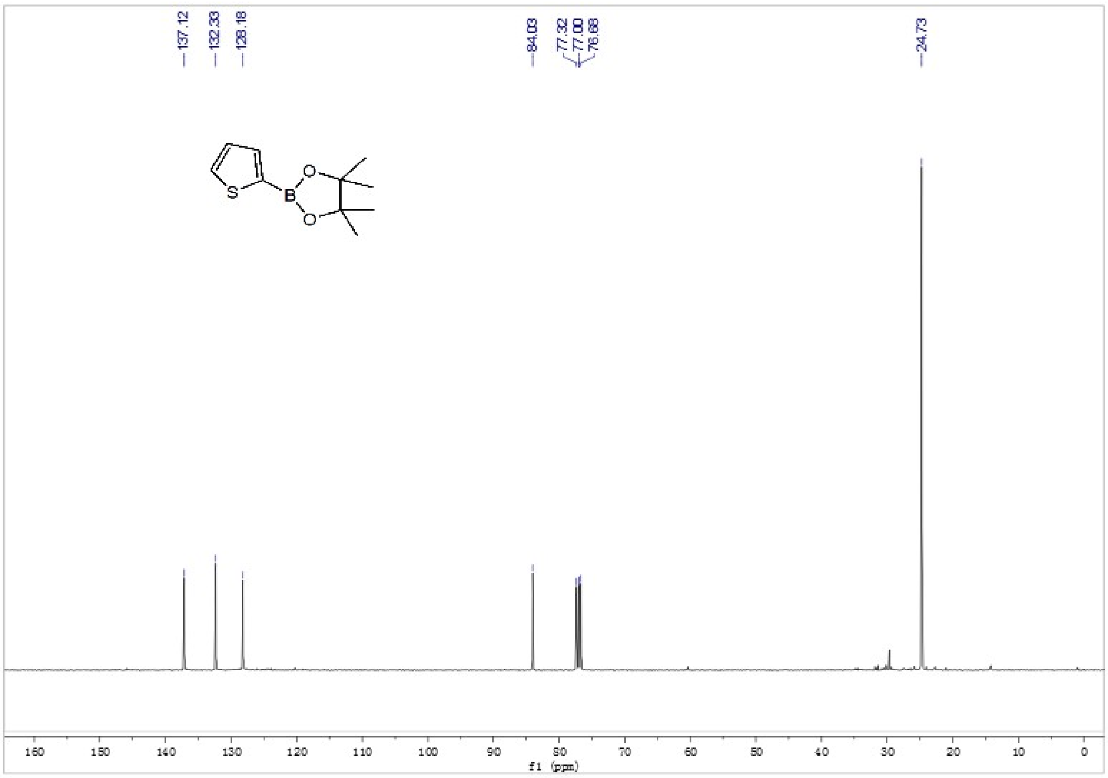

^{1}H and ^{13}C NMR spectra of compound 2m

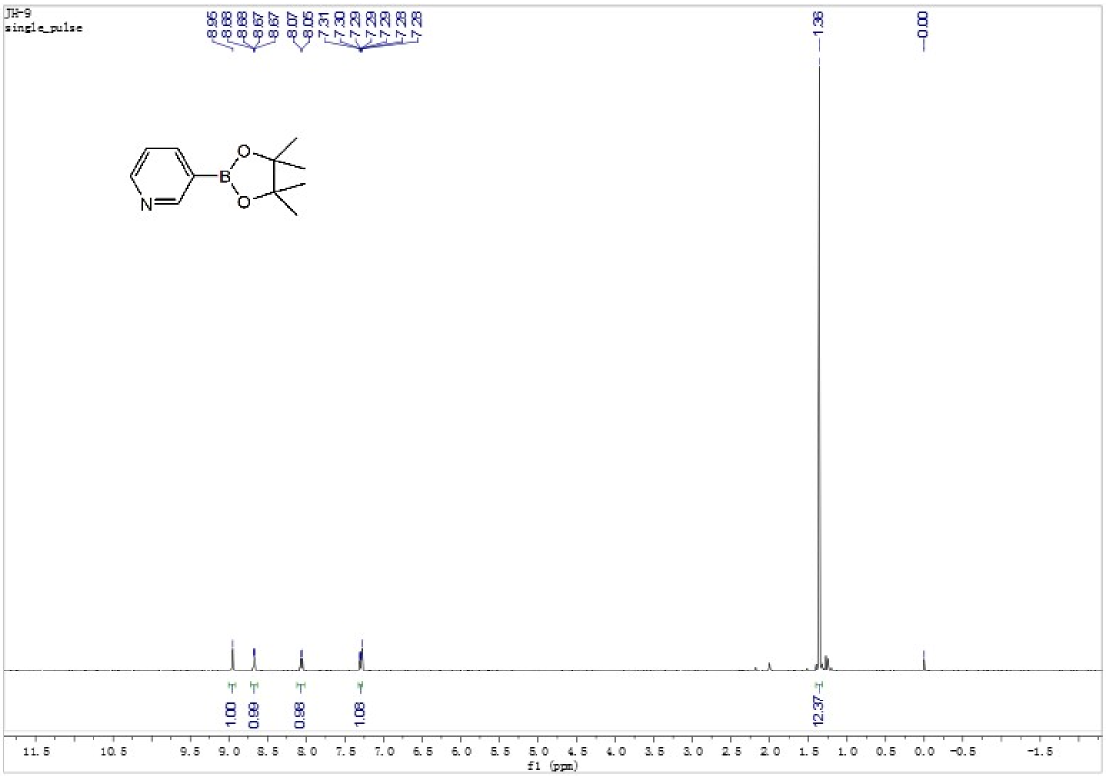

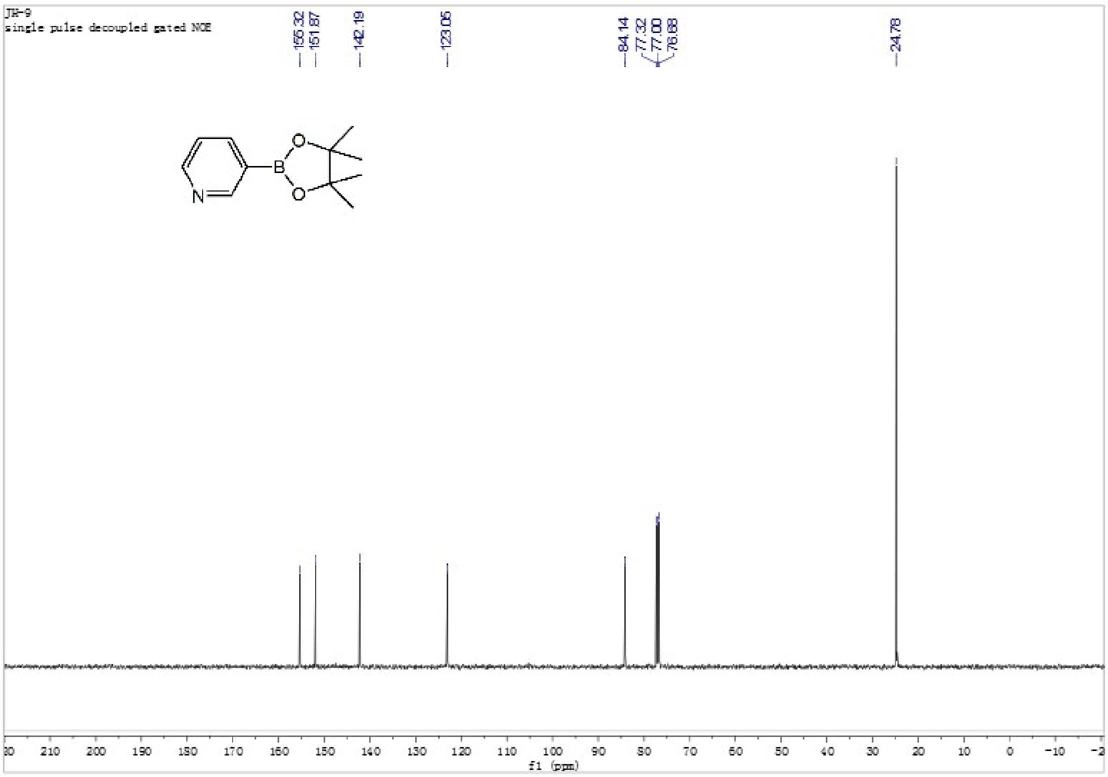

^{1}H and ^{13}C NMR spectra of compound 2n

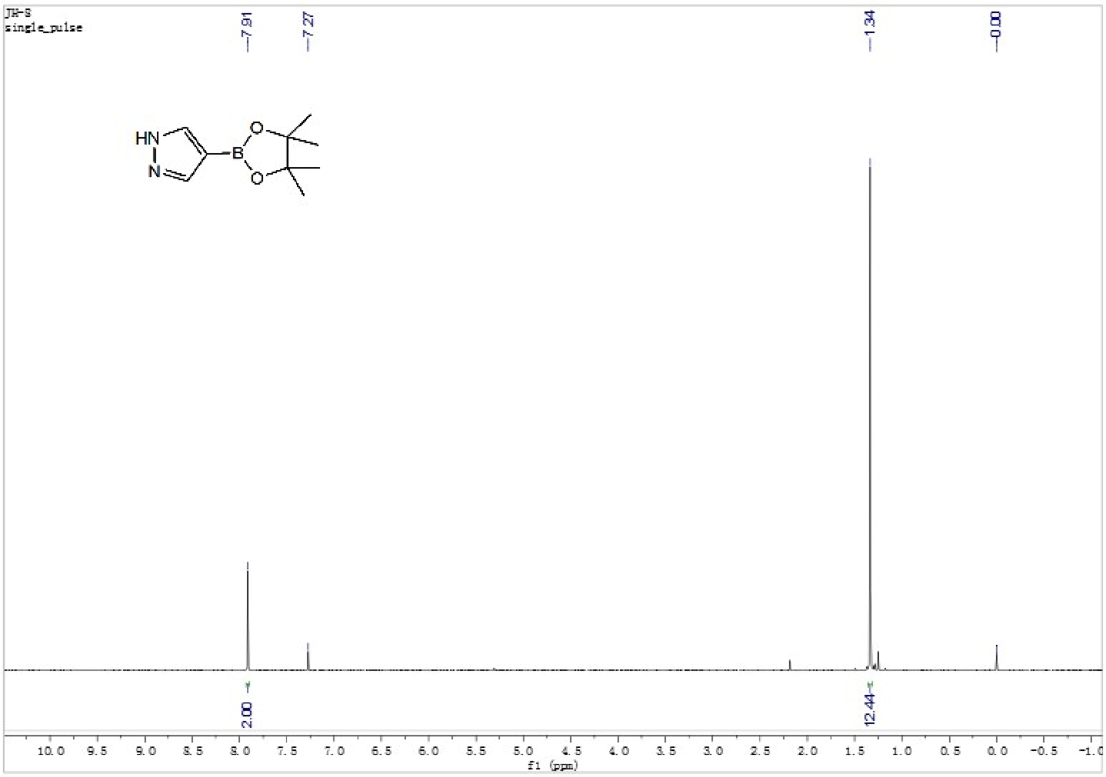

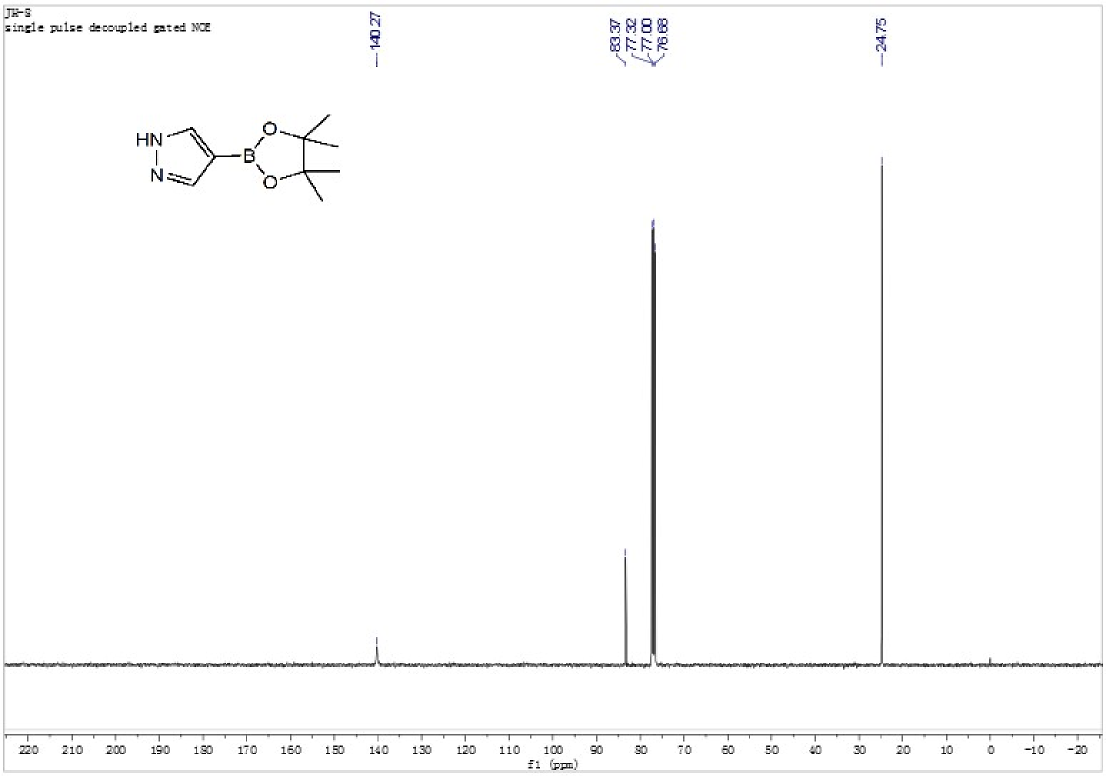

^{1}H and ^{13}C NMR spectra of compound 3a

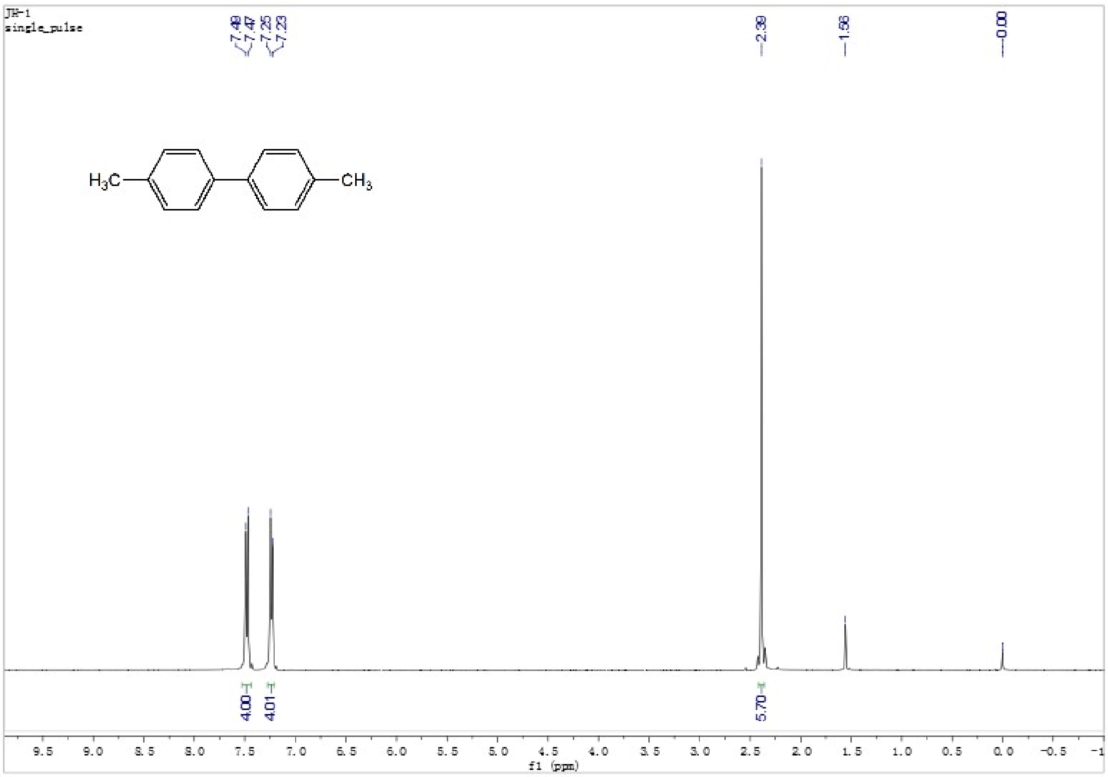

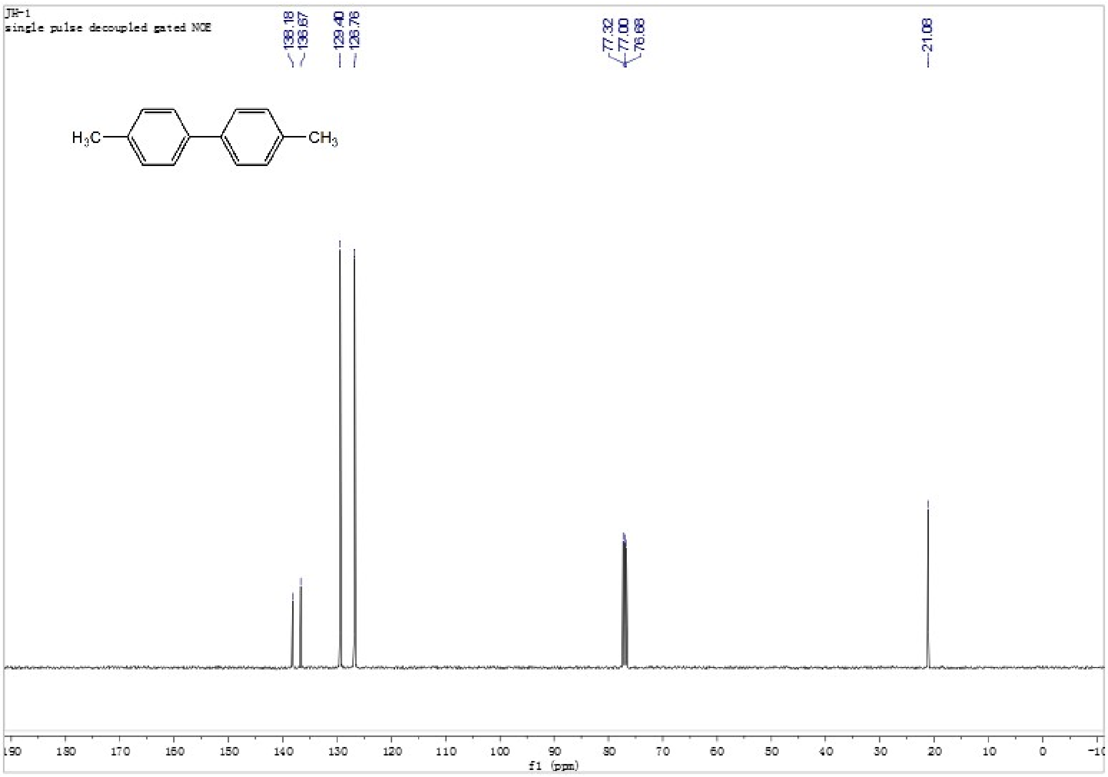

^{1}H and ^{13}C NMR spectra of compound 3b

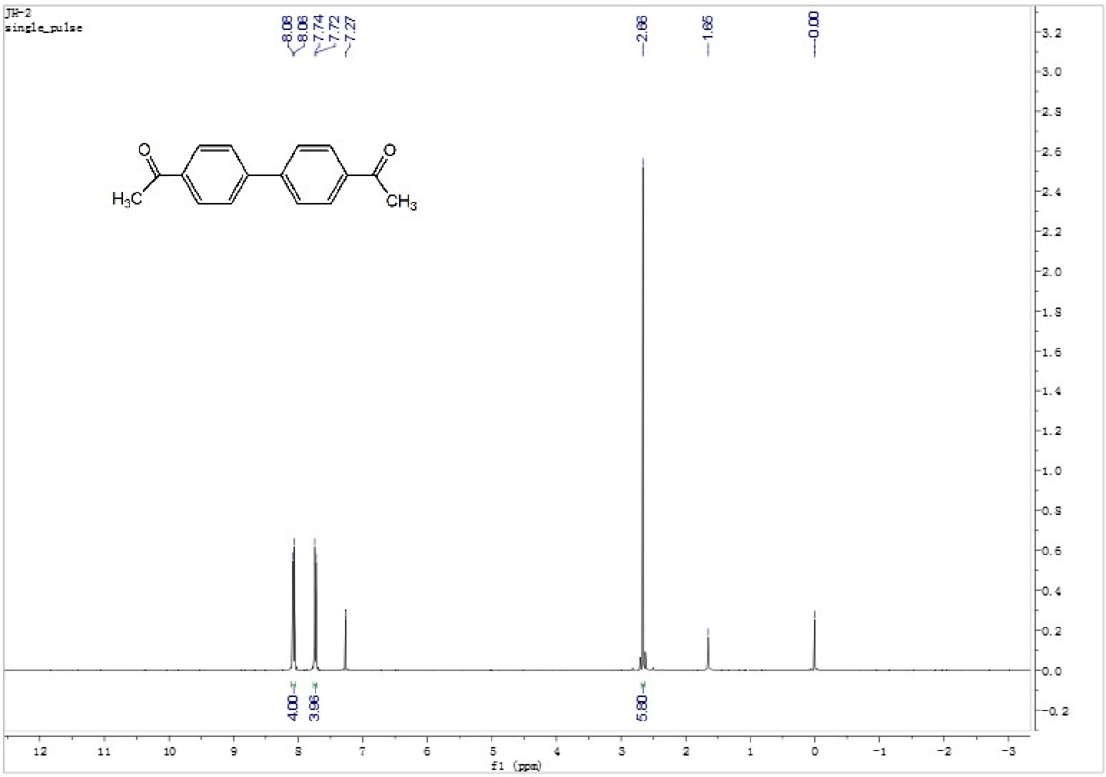

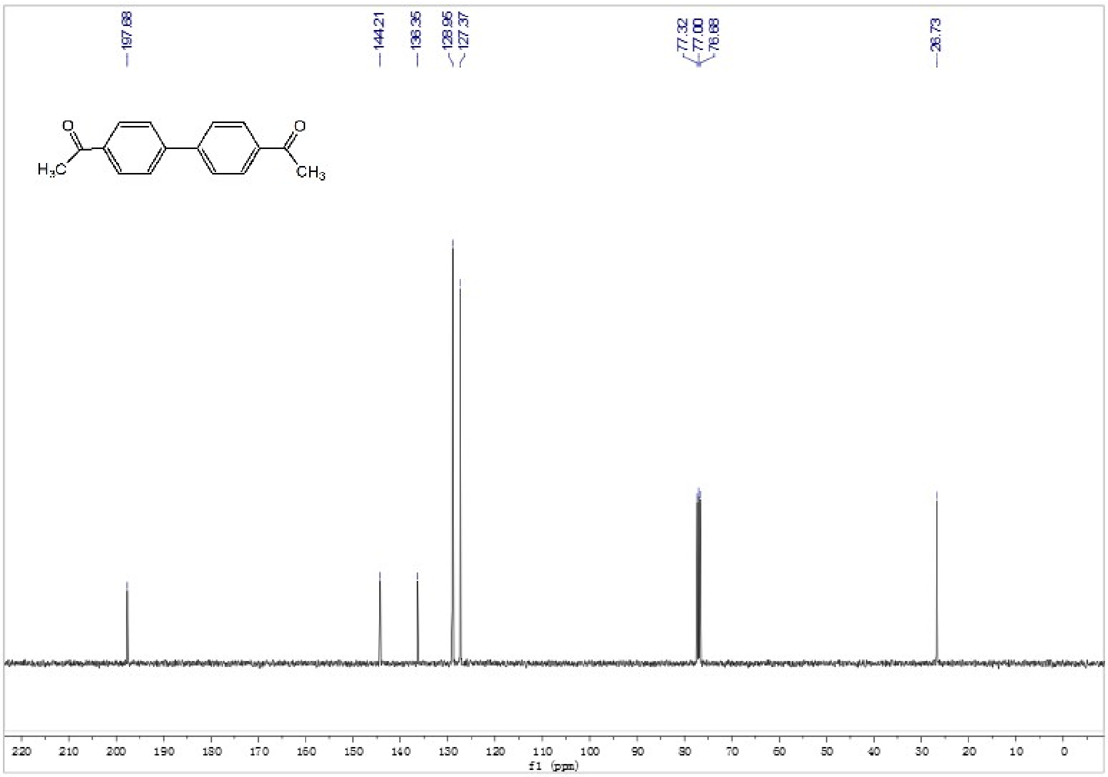

^{1}H and ^{13}C NMR spectra of compound 3c

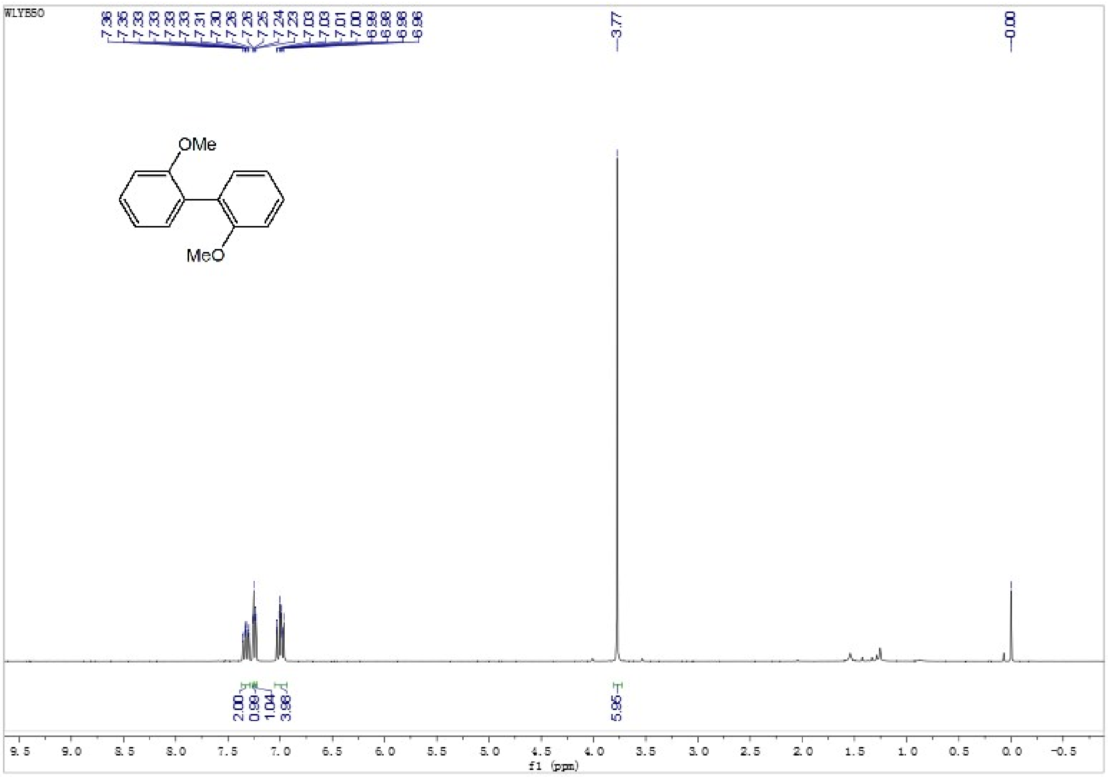

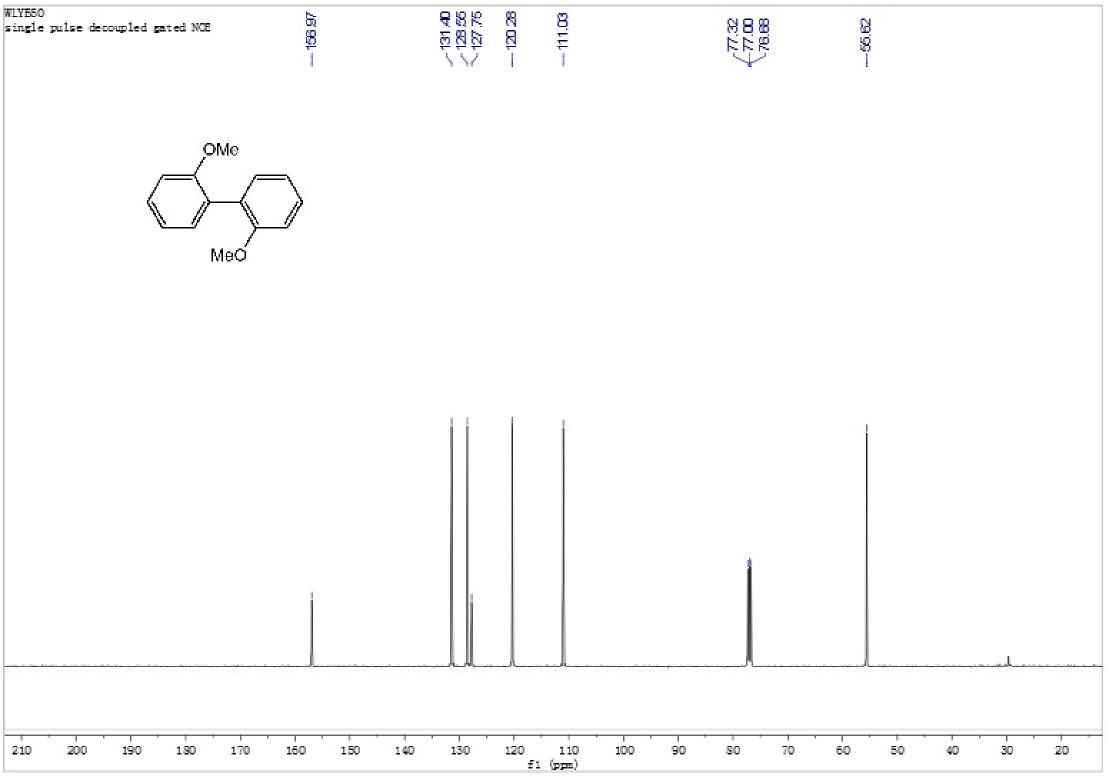

^{1}H and ^{13}C NMR spectra of compound 3d

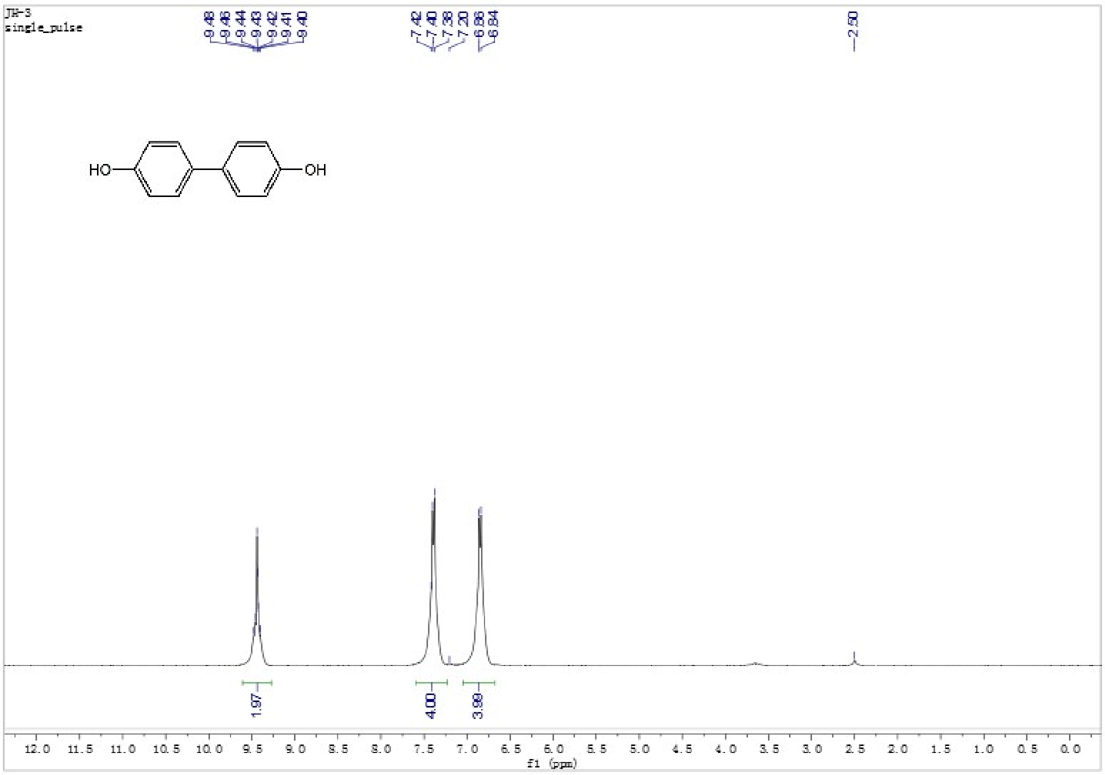

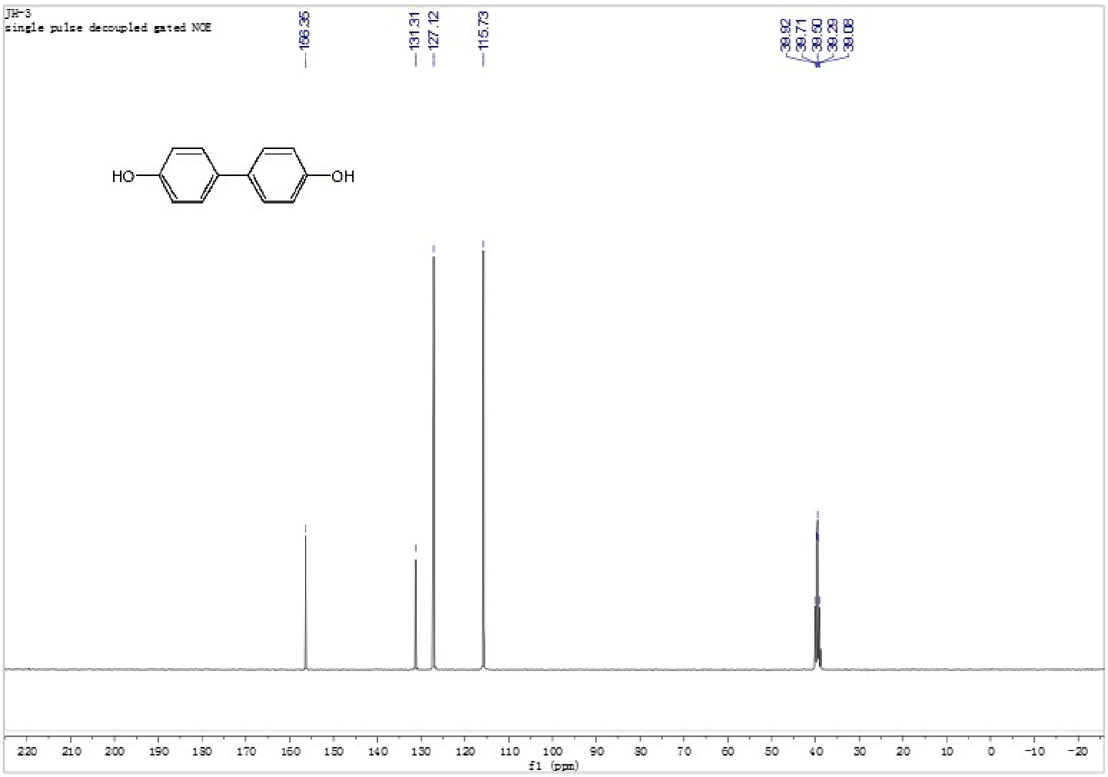

^{1}H and ^{13}C NMR spectra of compound 3e

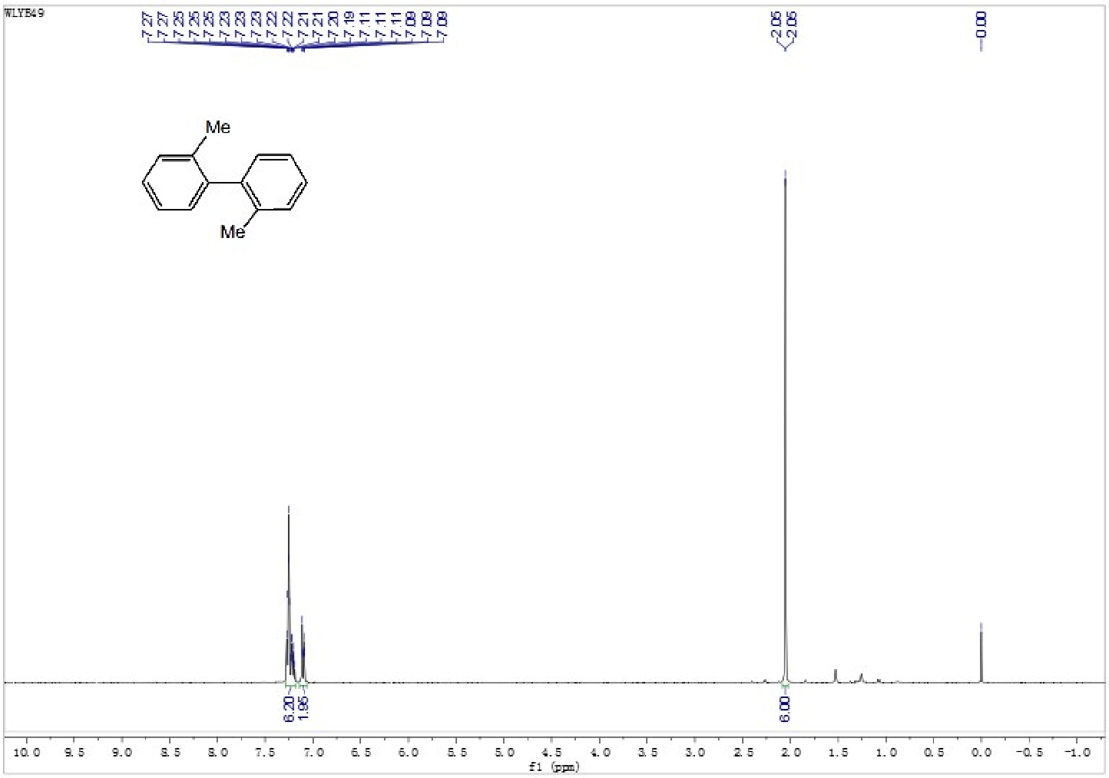

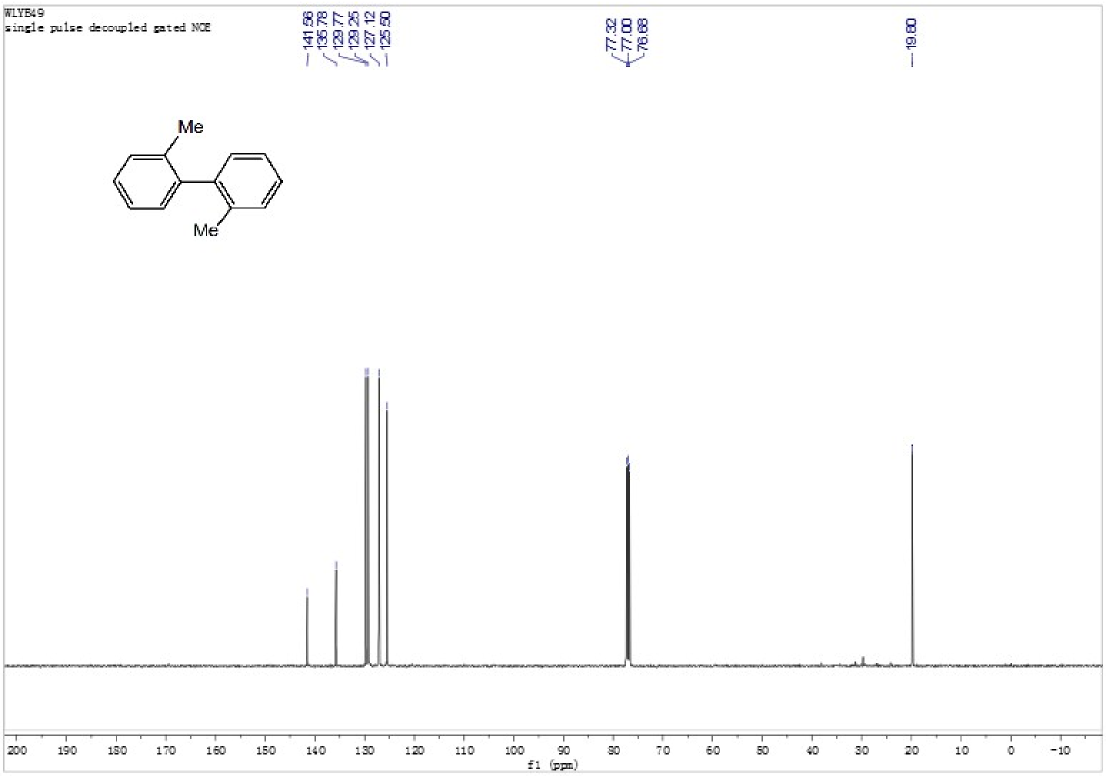

^{1}H and ^{13}C NMR spectra of compound 3f

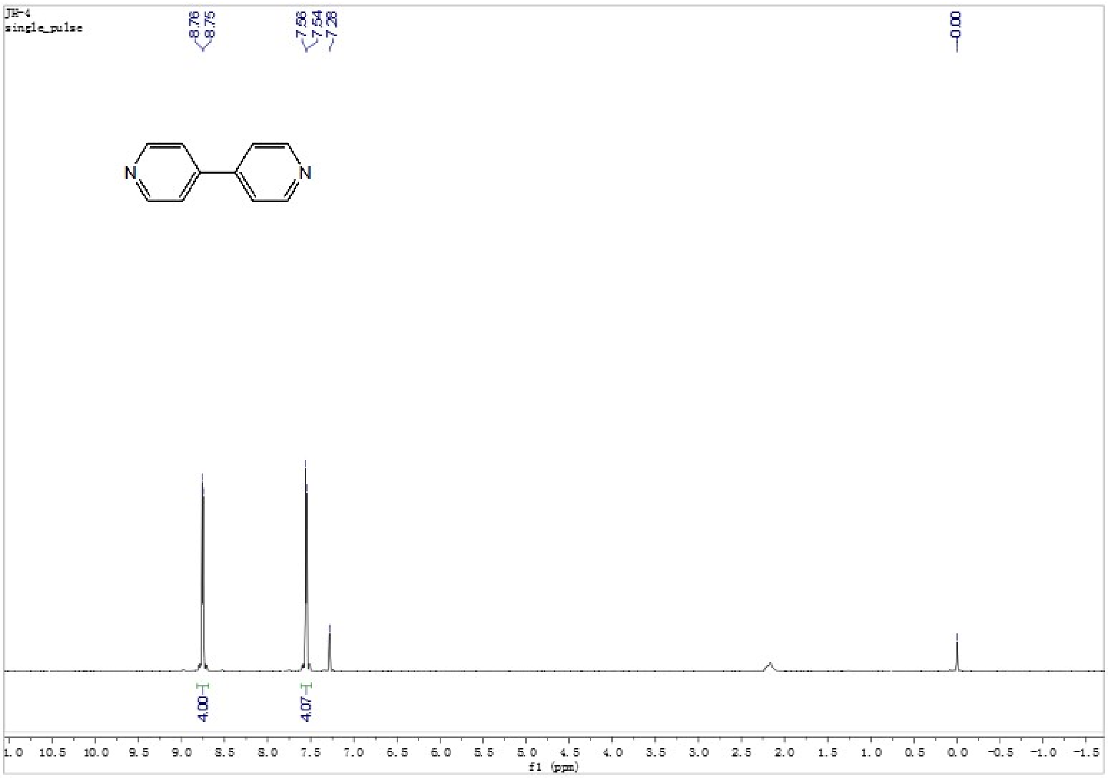

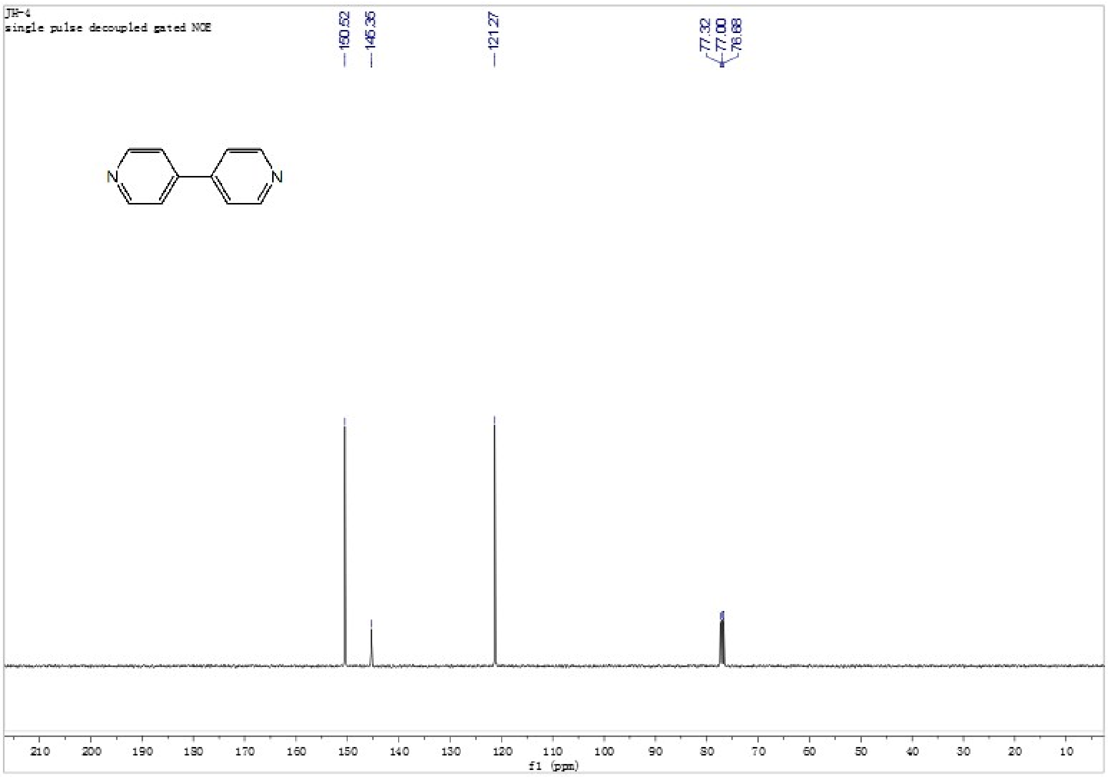

^{1}H and ^{13}C NMR spectra of compound 3g

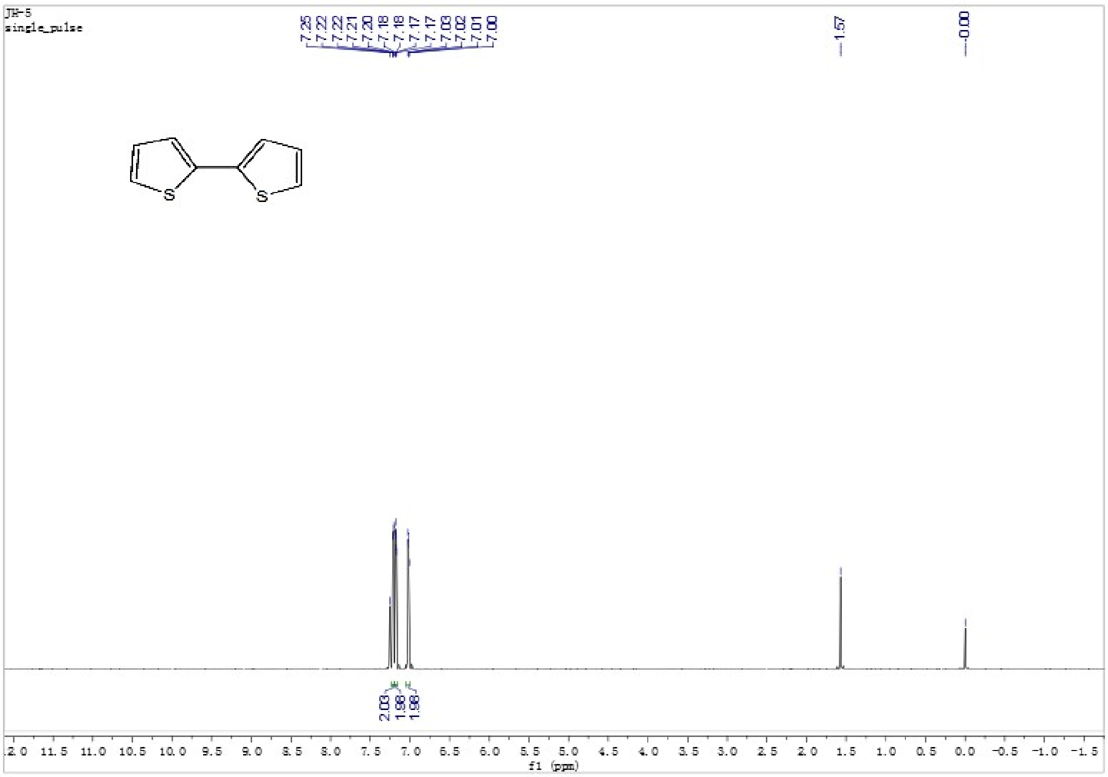

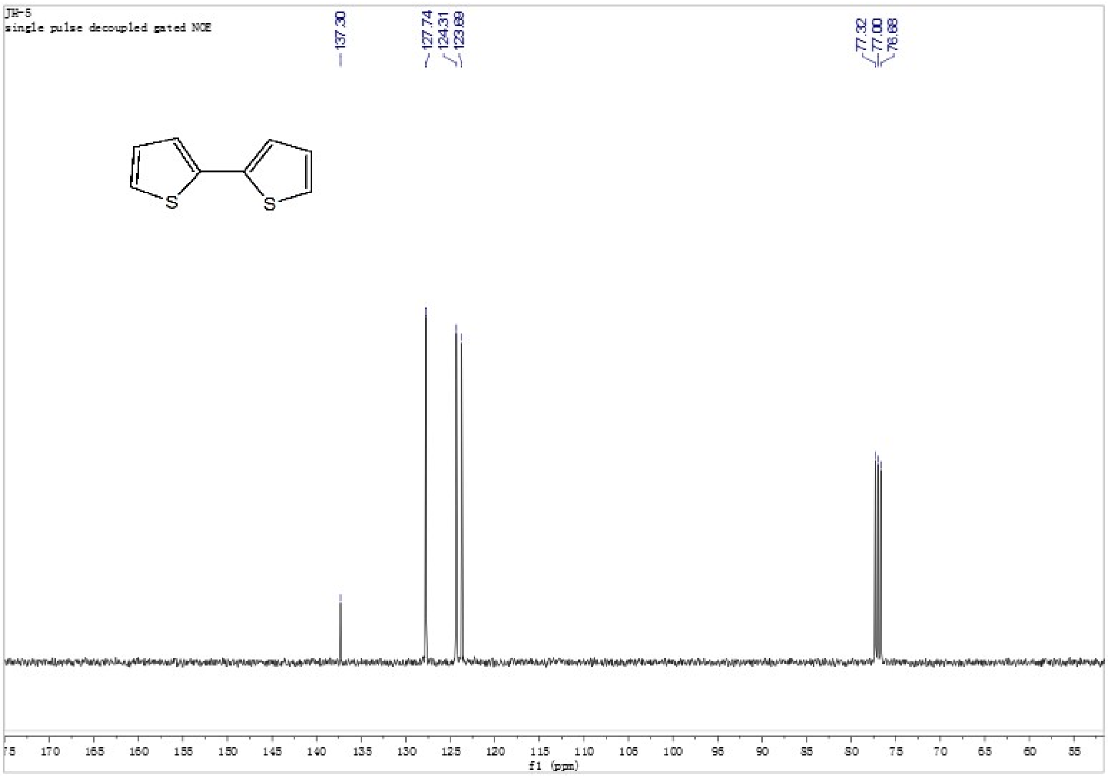

^{1}H and ^{13}C NMR spectra of compound 4a

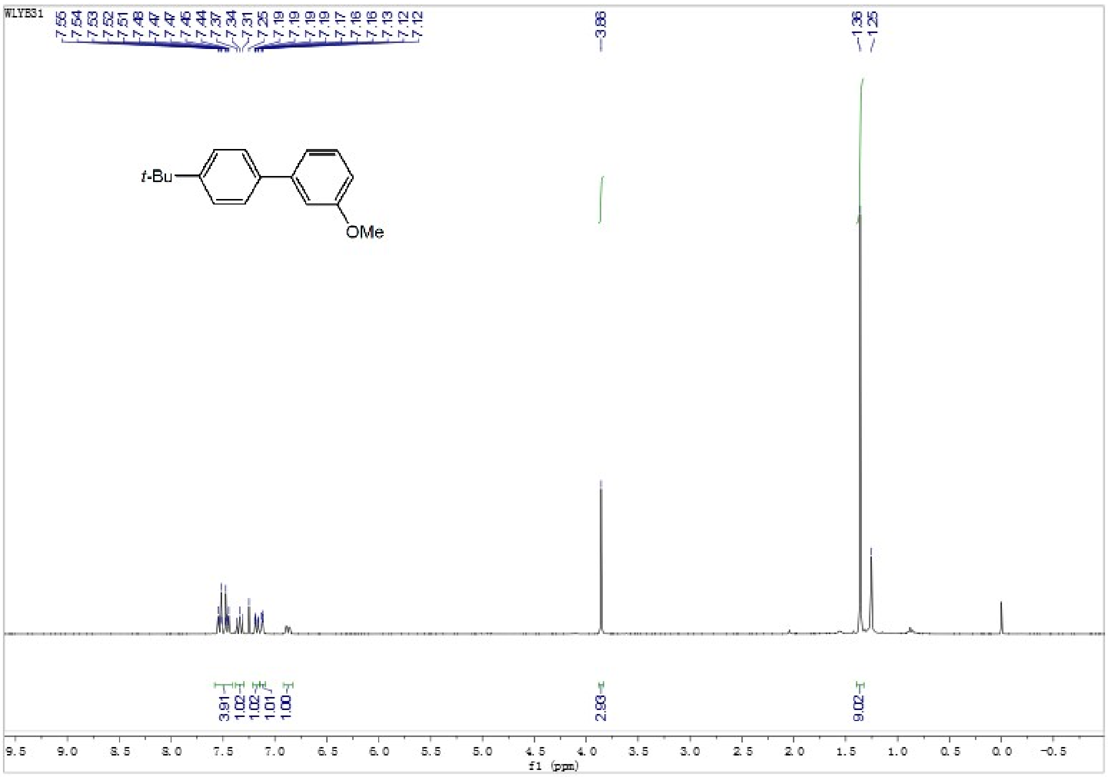

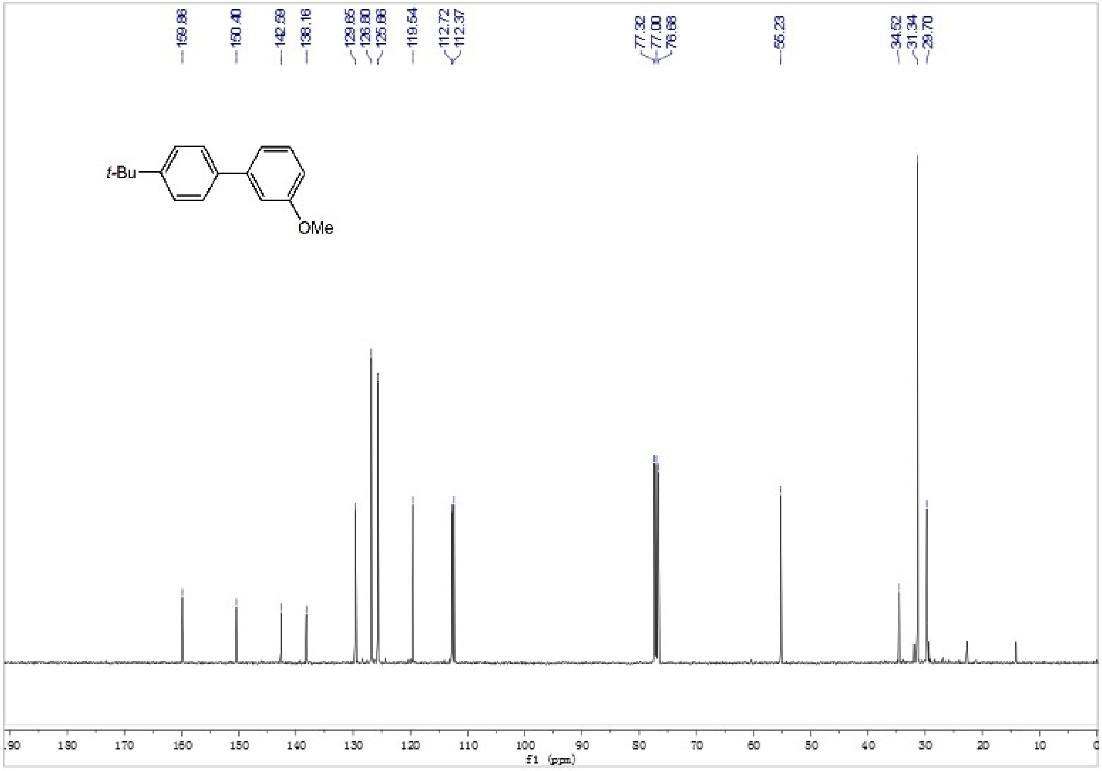

^{1}H and ^{13}C NMR spectra of compound 4b

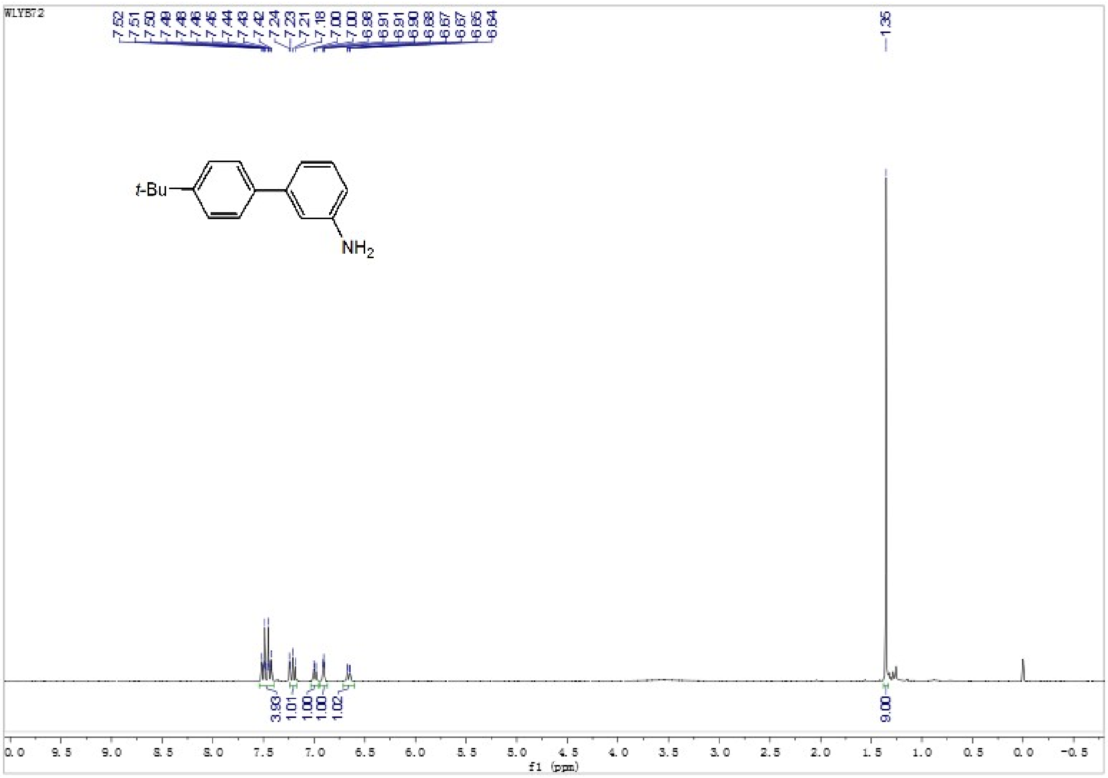

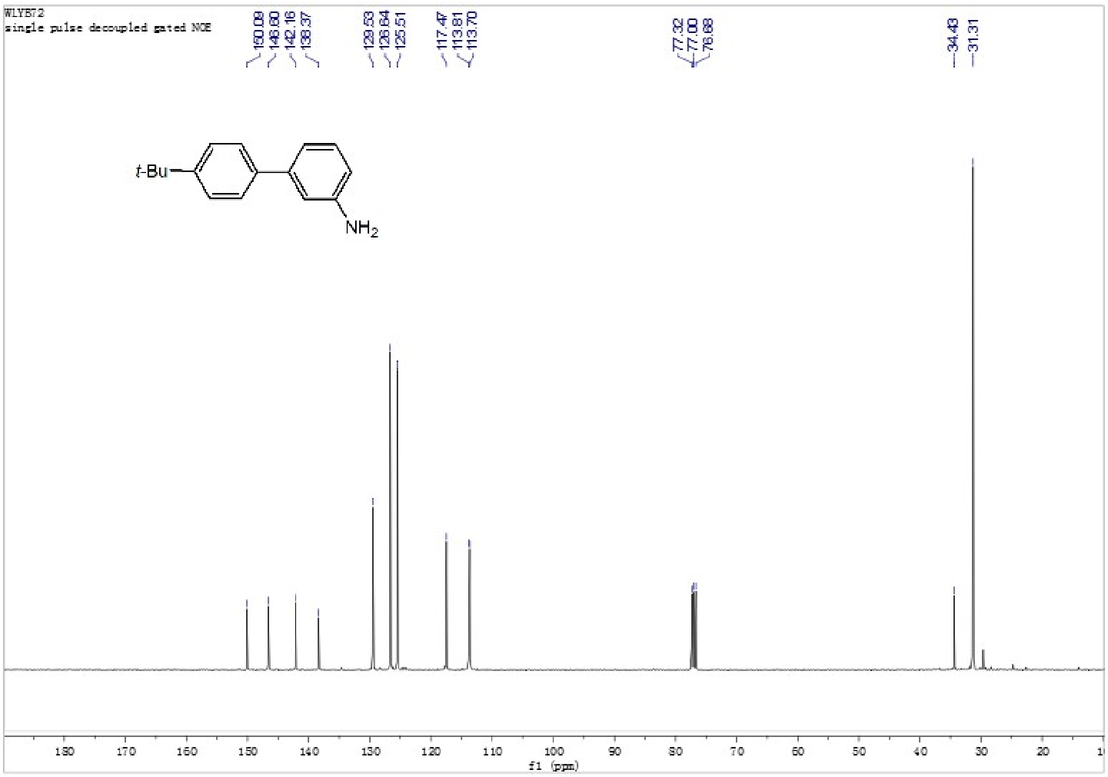

^{1}H and ^{13}C NMR spectra of compound 4c

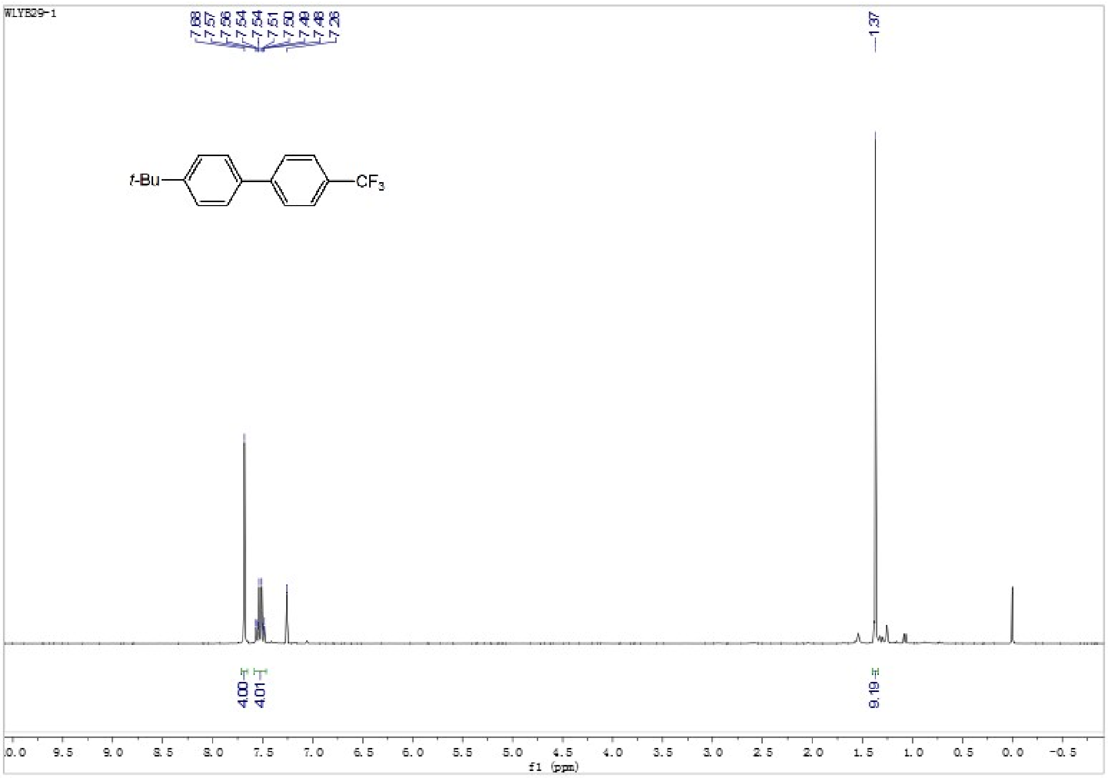

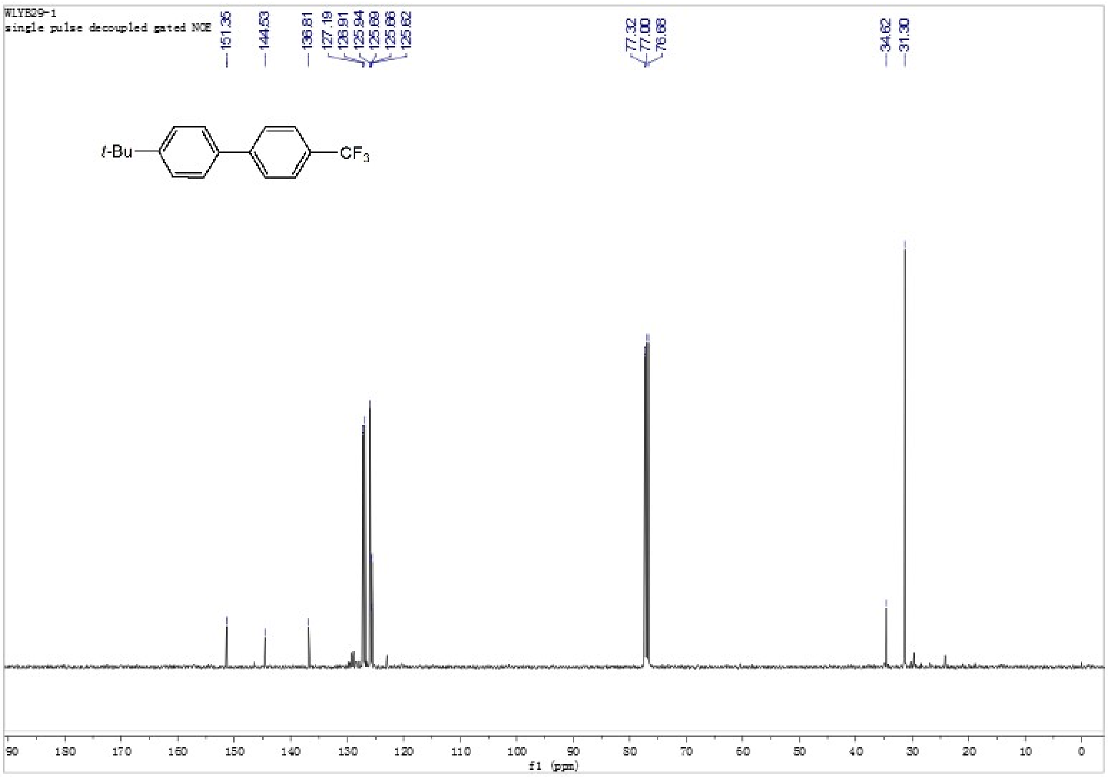

^{1}H and ^{13}C NMR spectra of compound 4d

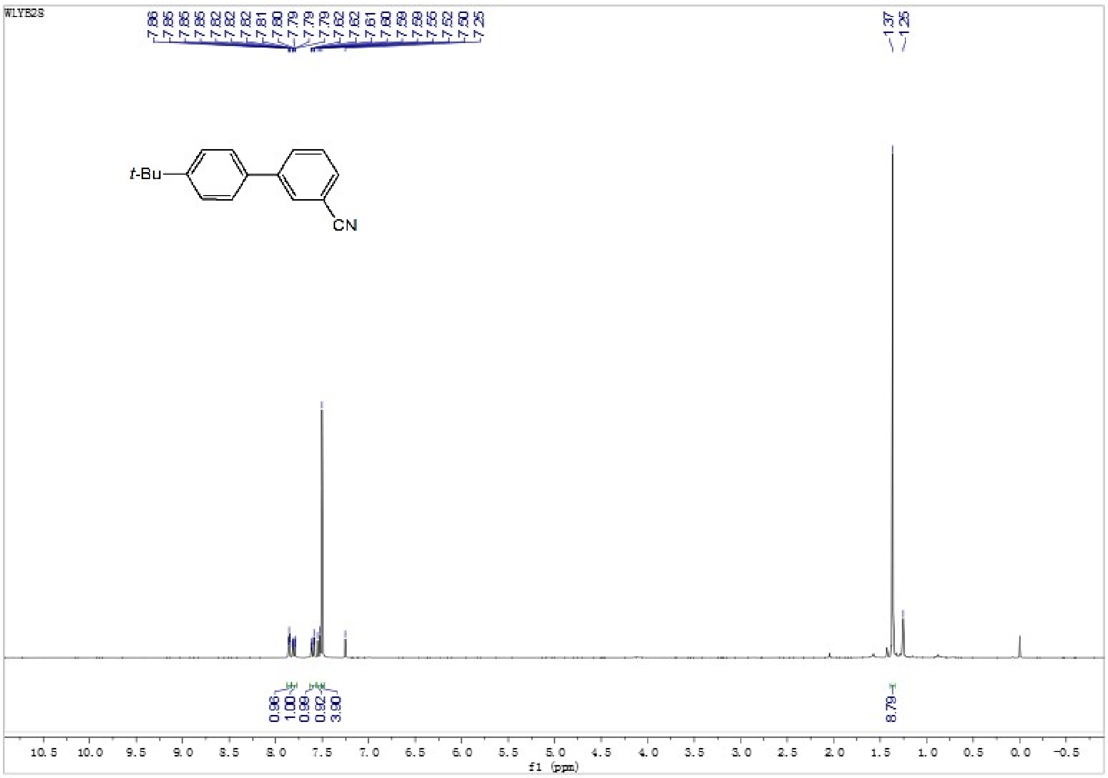

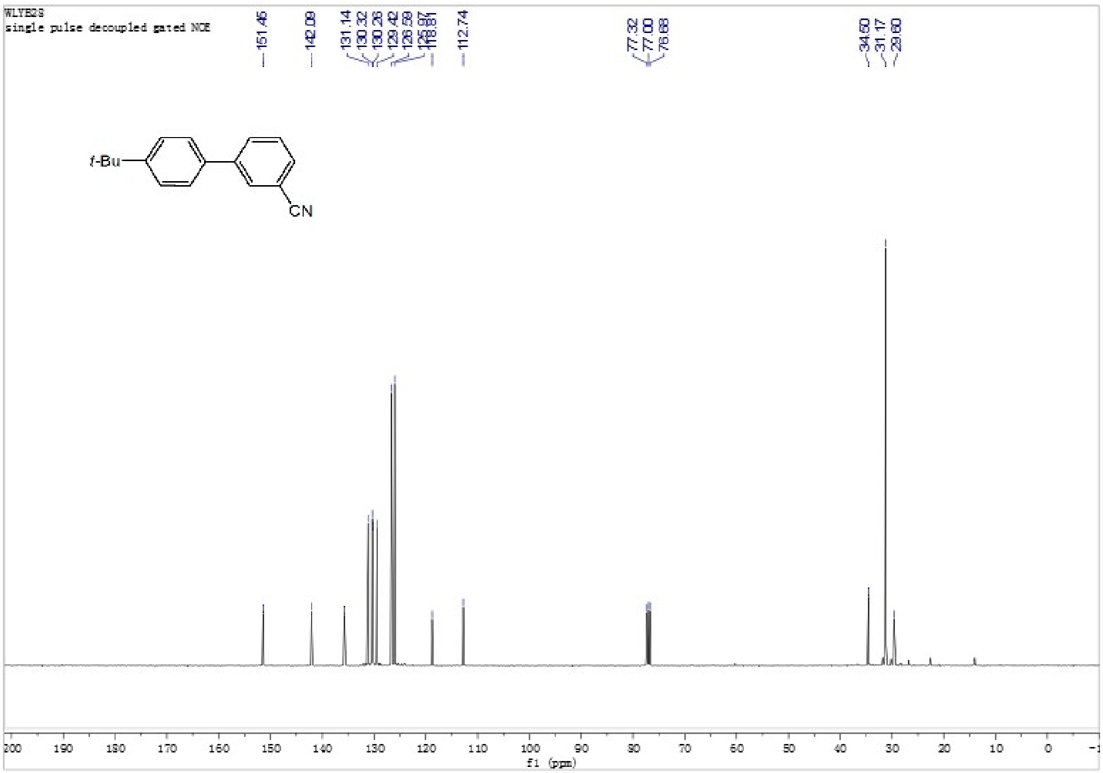

^{1}H and ^{13}C NMR spectra of compound 4e

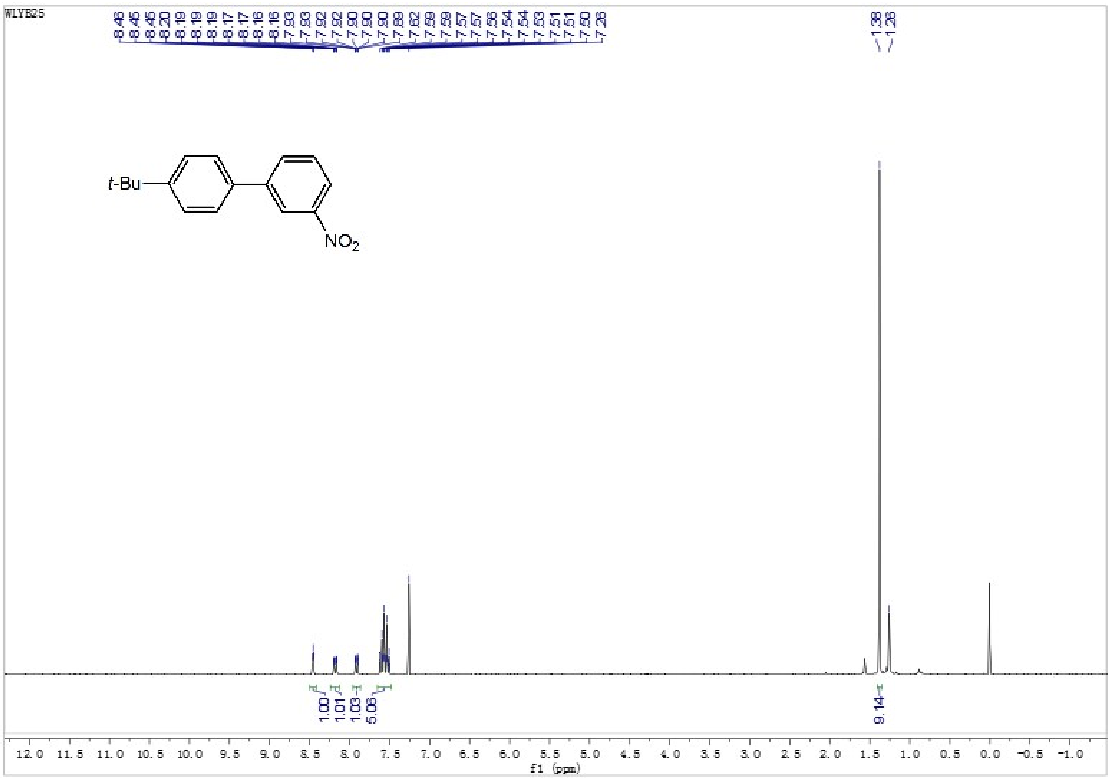

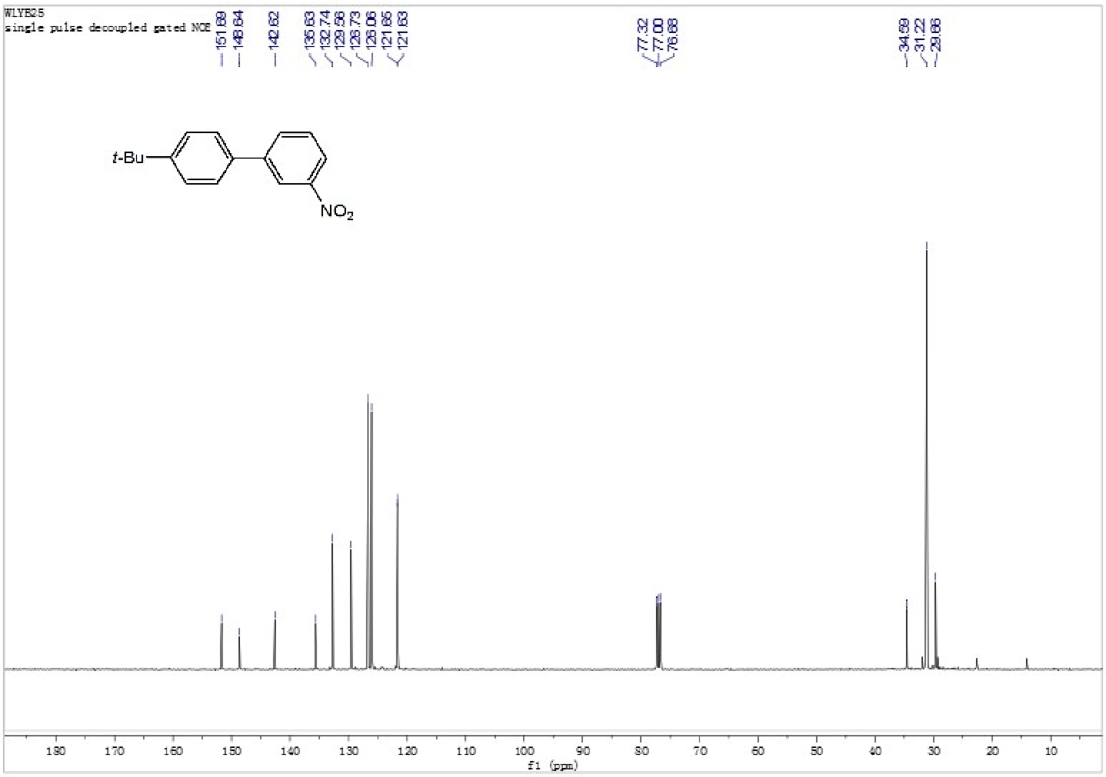

^1H and ^{13}C NMR spectra of compound 4f

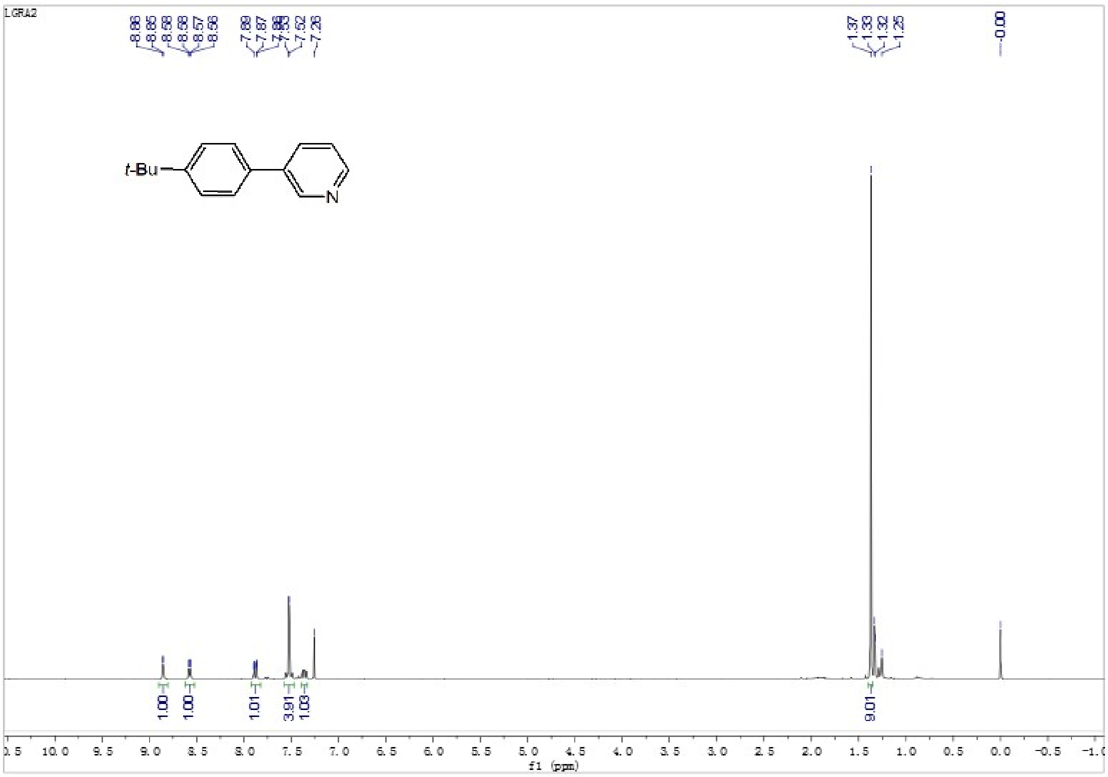

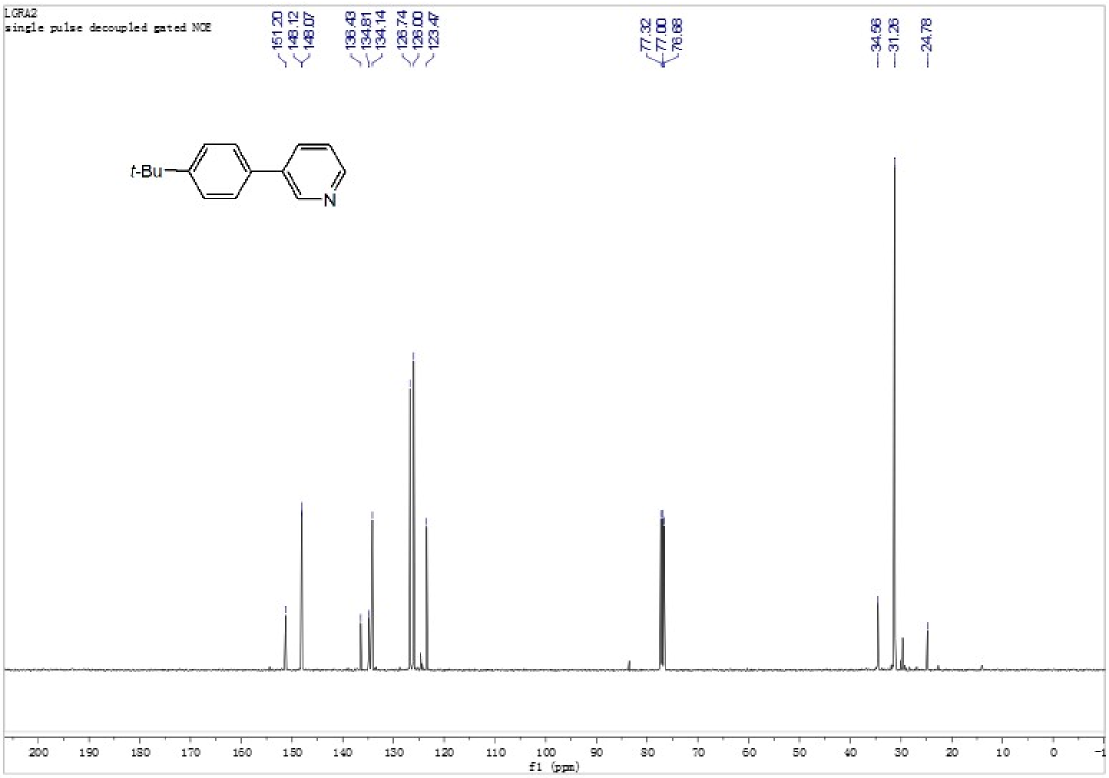

^{1}H and ^{13}C NMR spectra of compound 4g

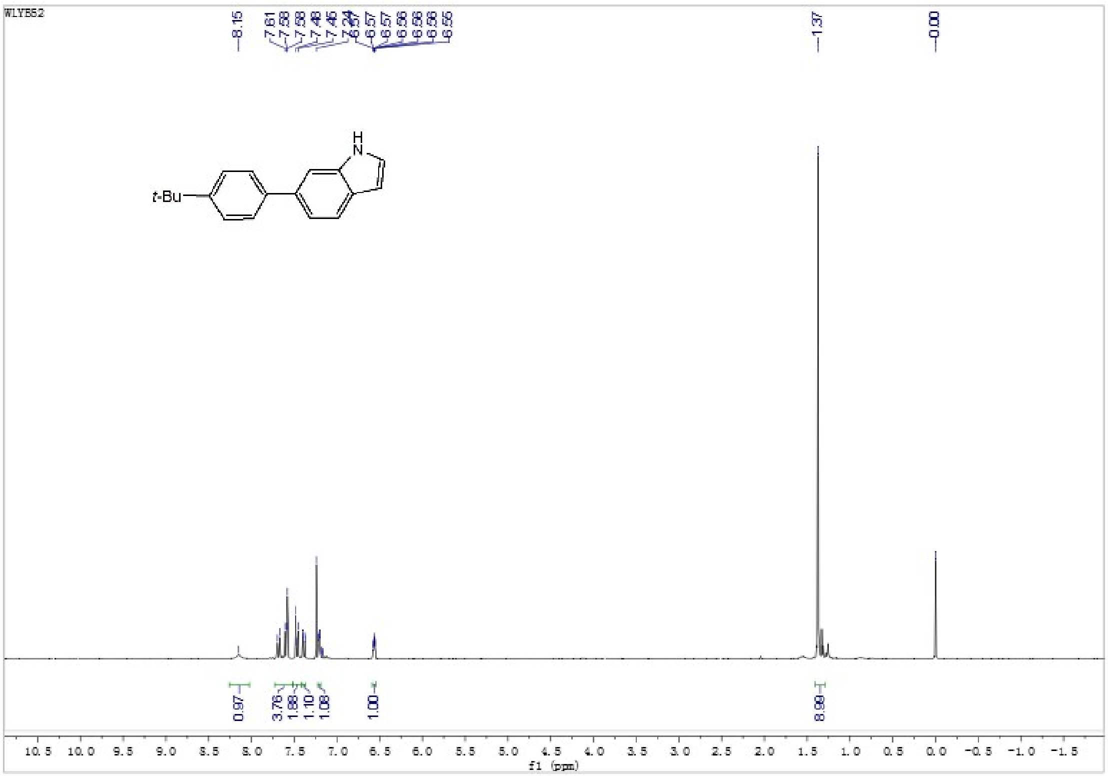

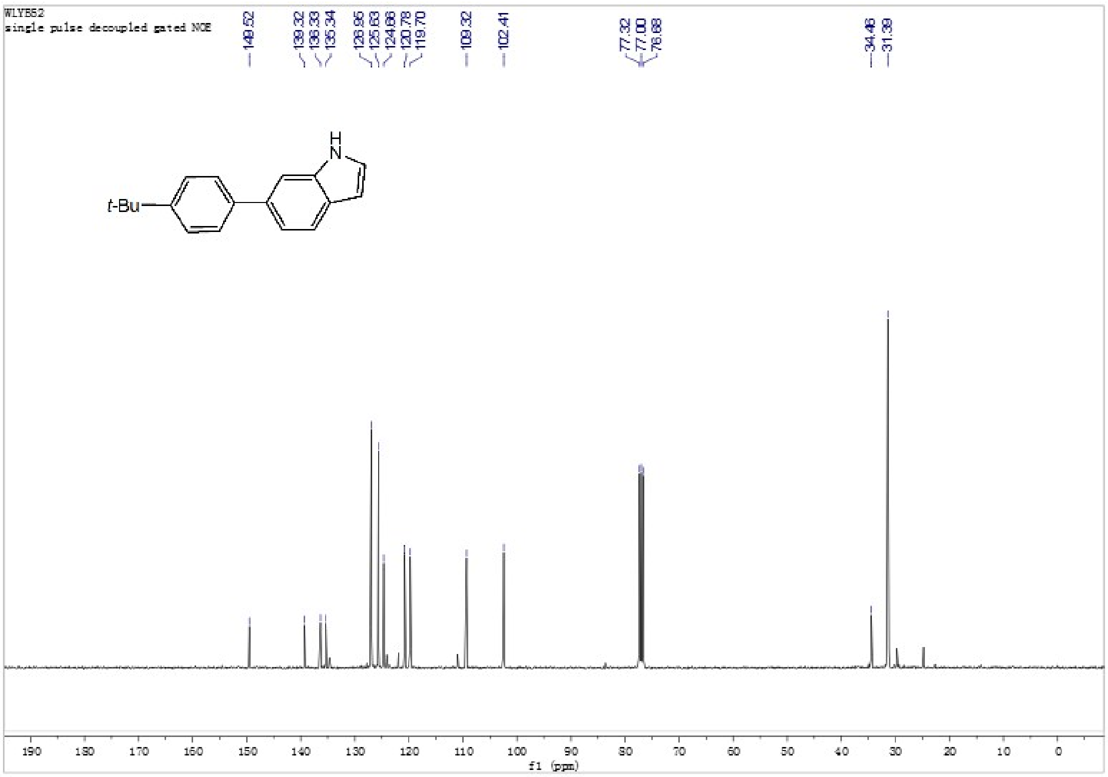

^{1}H and ^{13}C NMR spectra of compound 4h

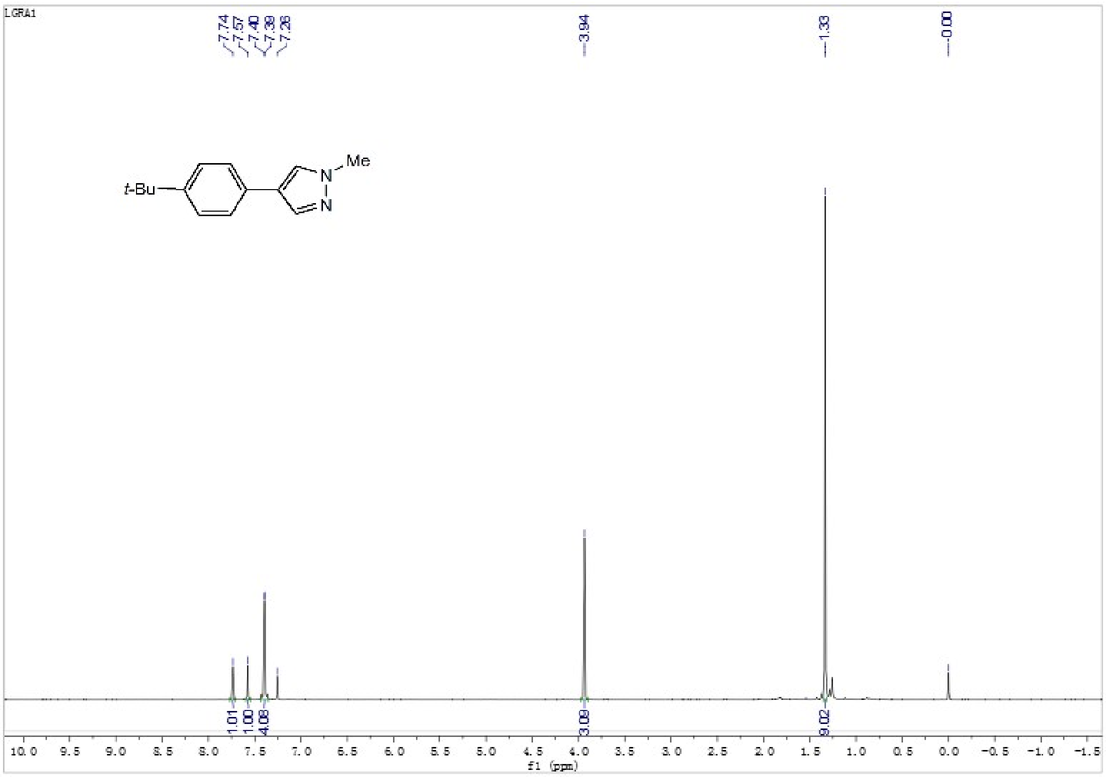

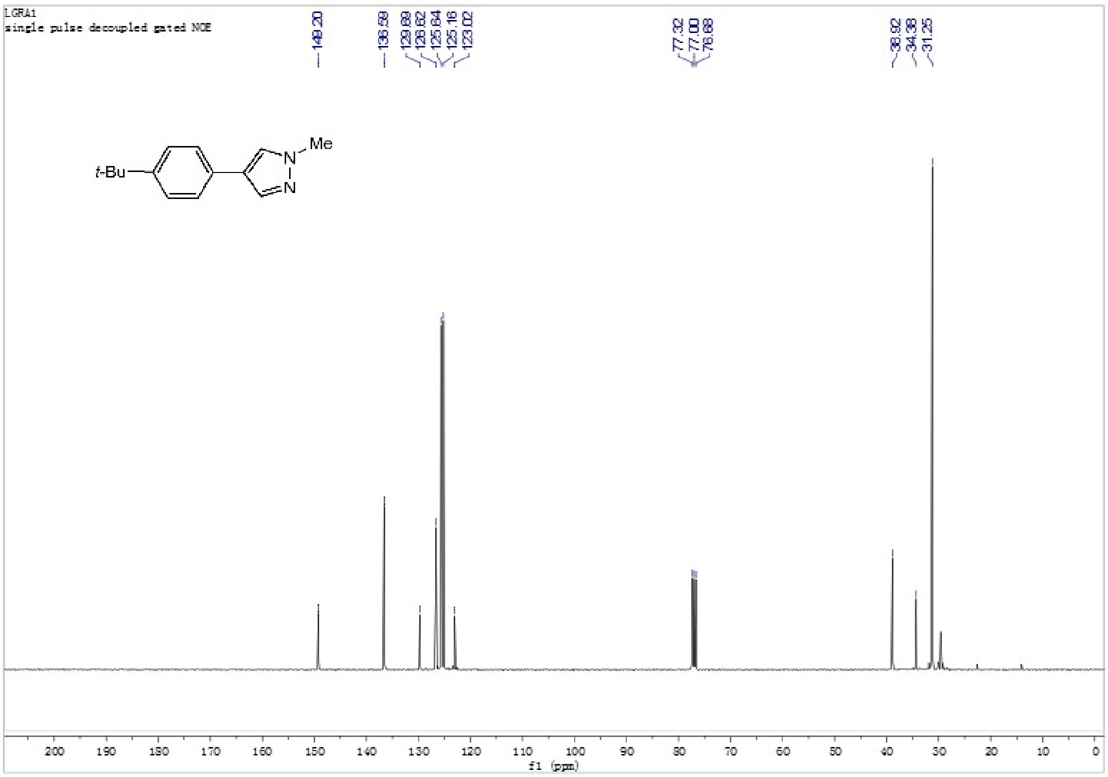

^{1}H and ^{13}C NMR spectra of compound 6a

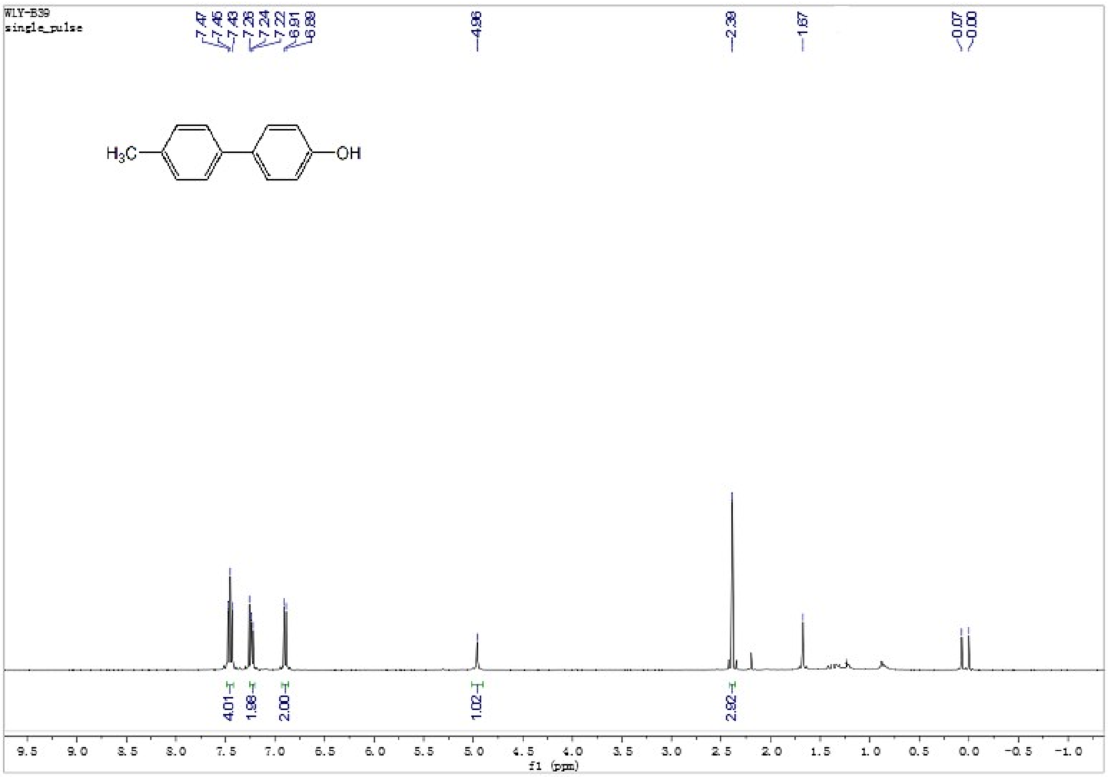

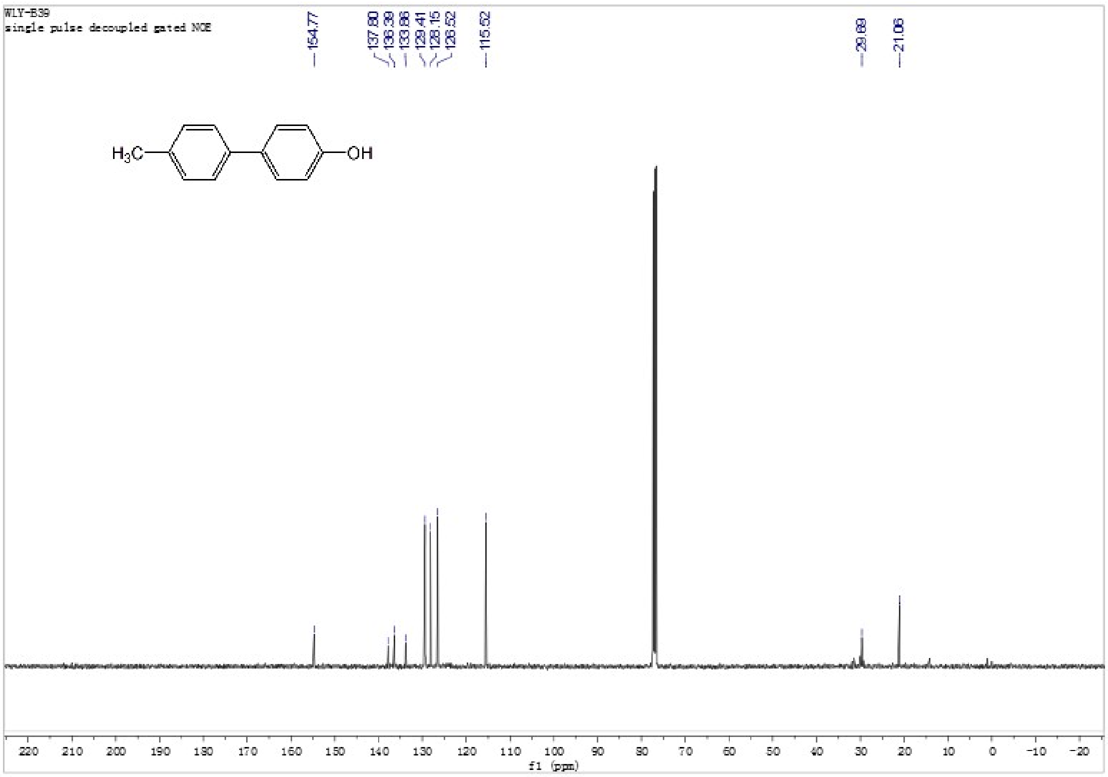

^{1}H and ^{13}C NMR spectra of compound 6b

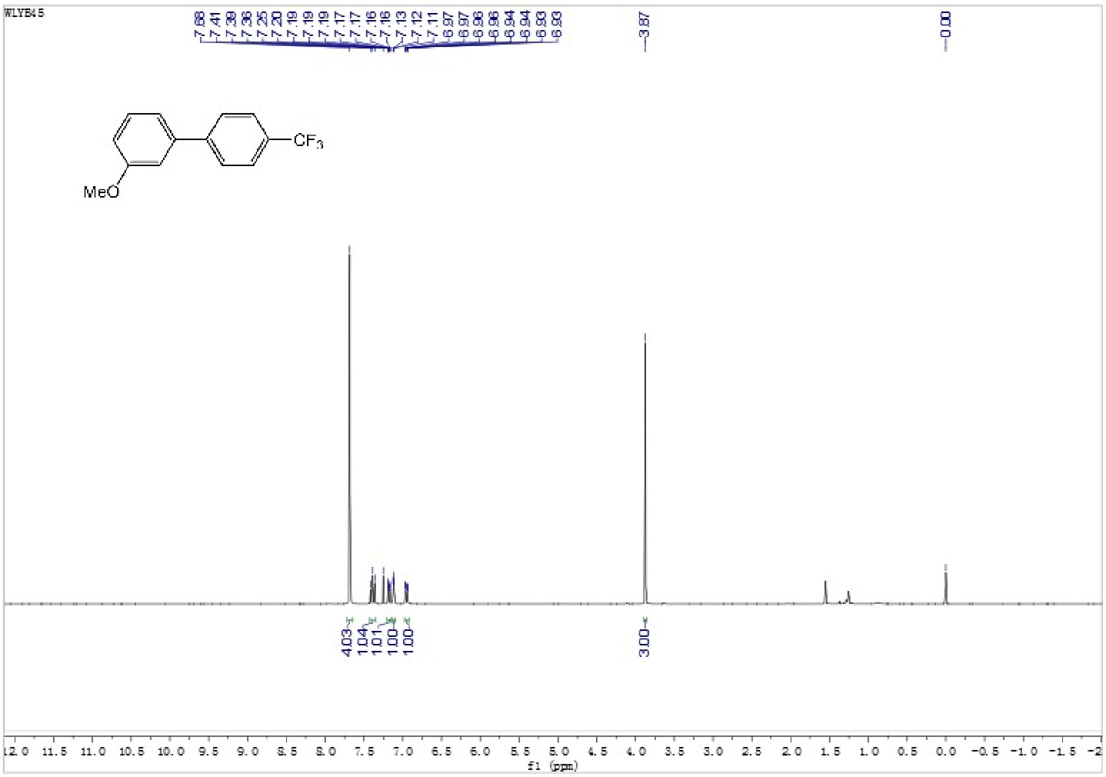

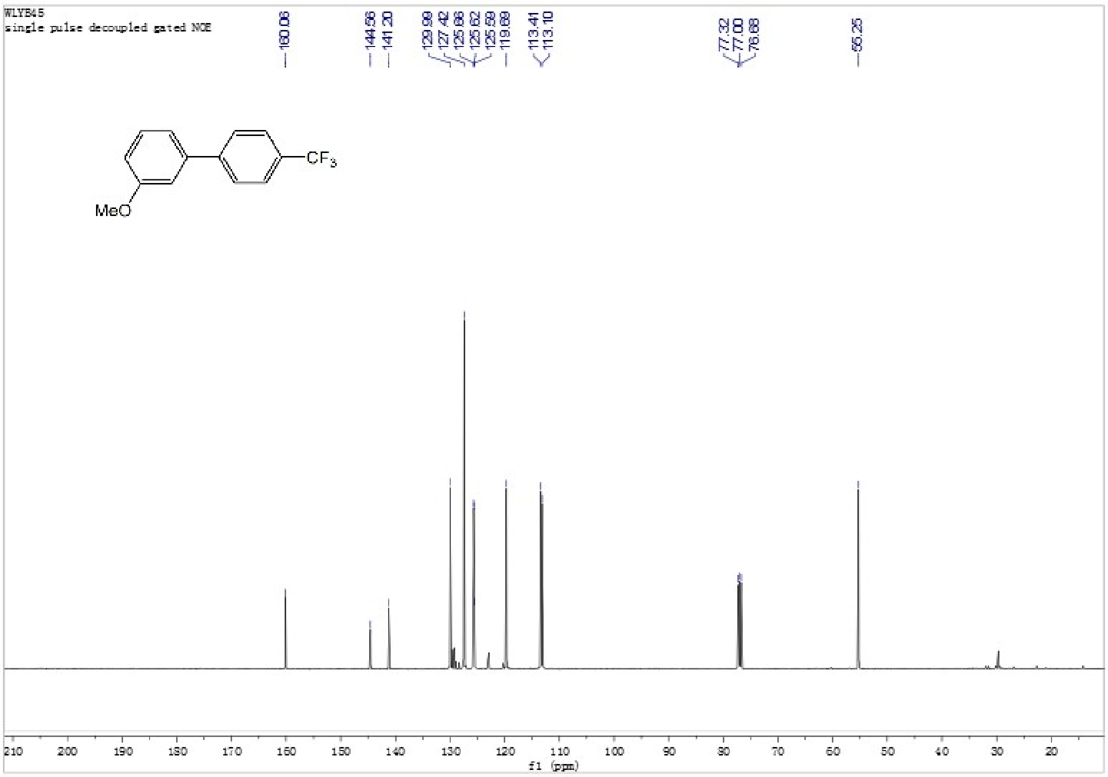

^{1}H and ^{13}C NMR spectra of compound 6c

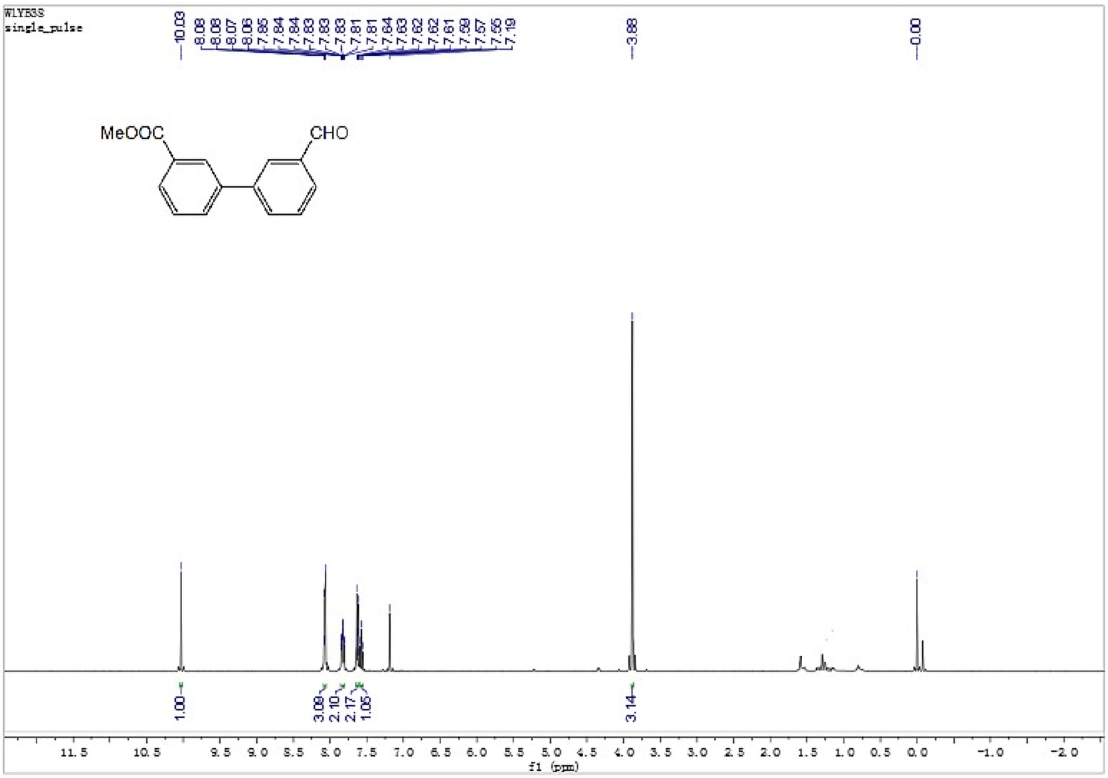

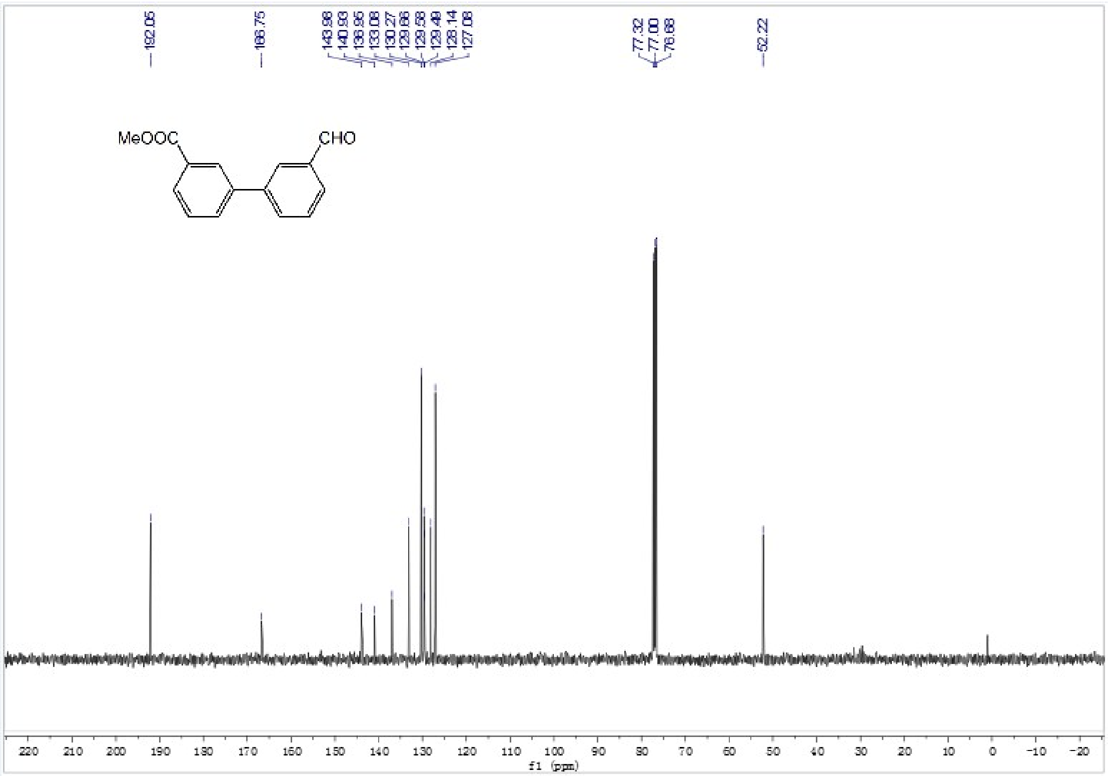

^{1}H and ^{13}C NMR spectra of compound 6d

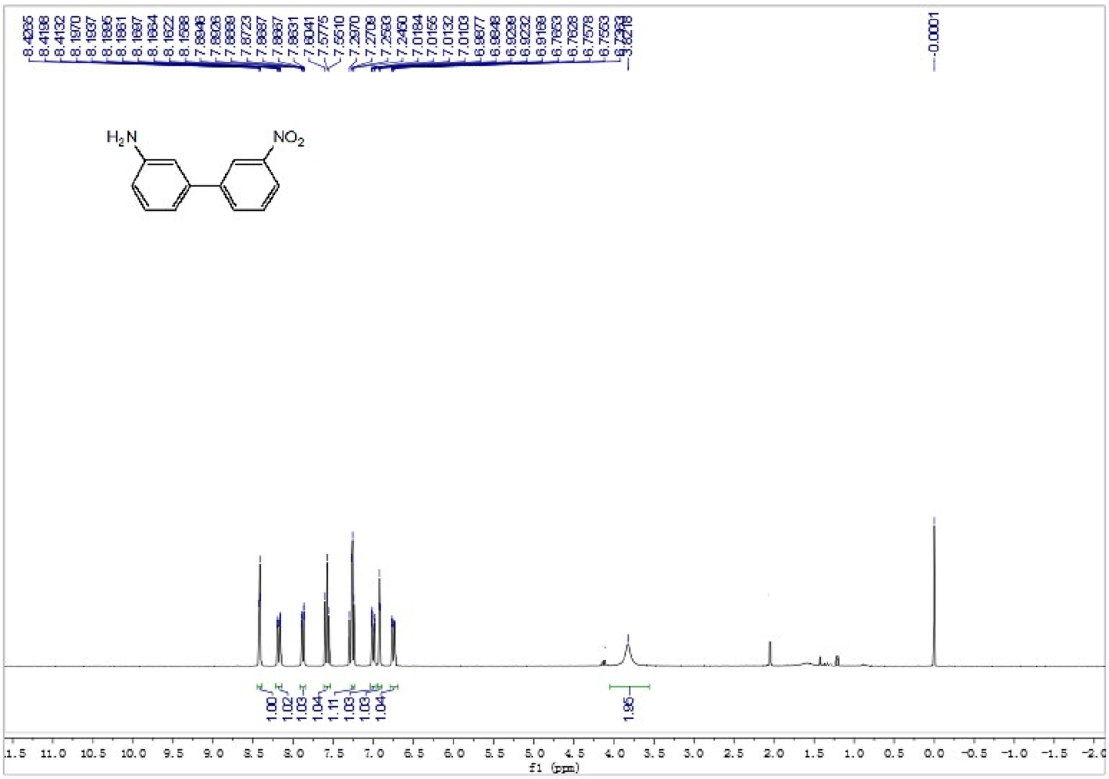

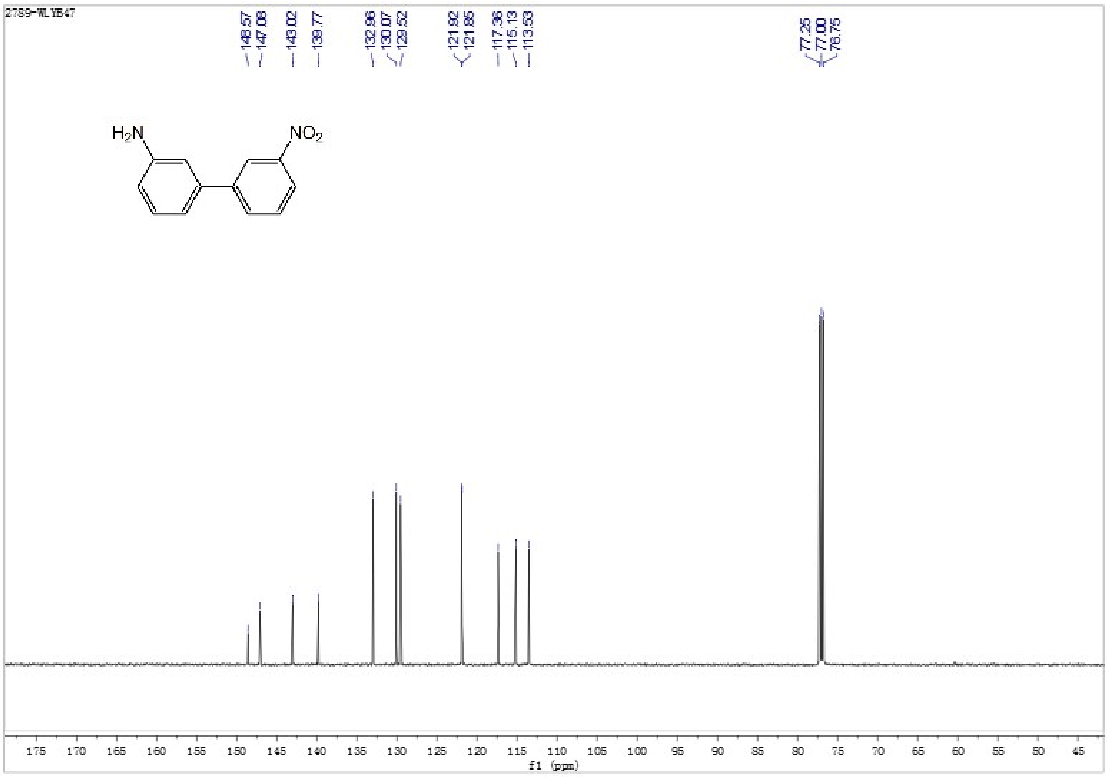

^{1}H and ^{13}C NMR spectra of compound 6e

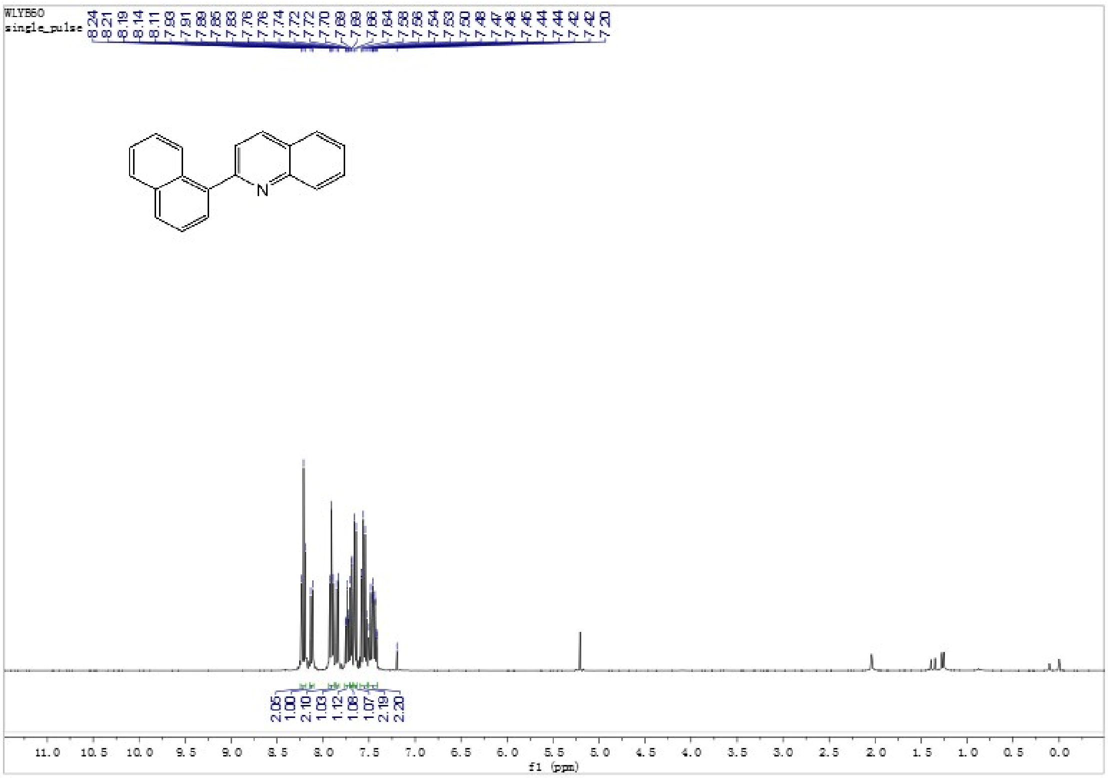

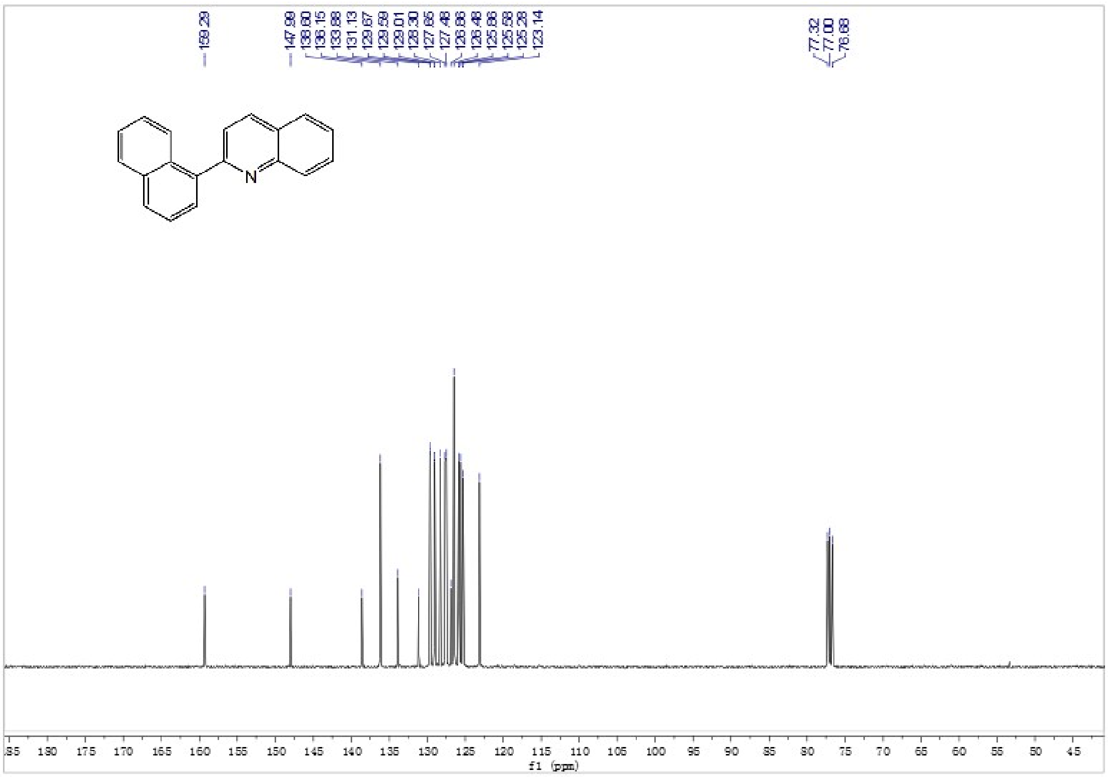

^{1}H and ^{13}C NMR spectra of compound 6f

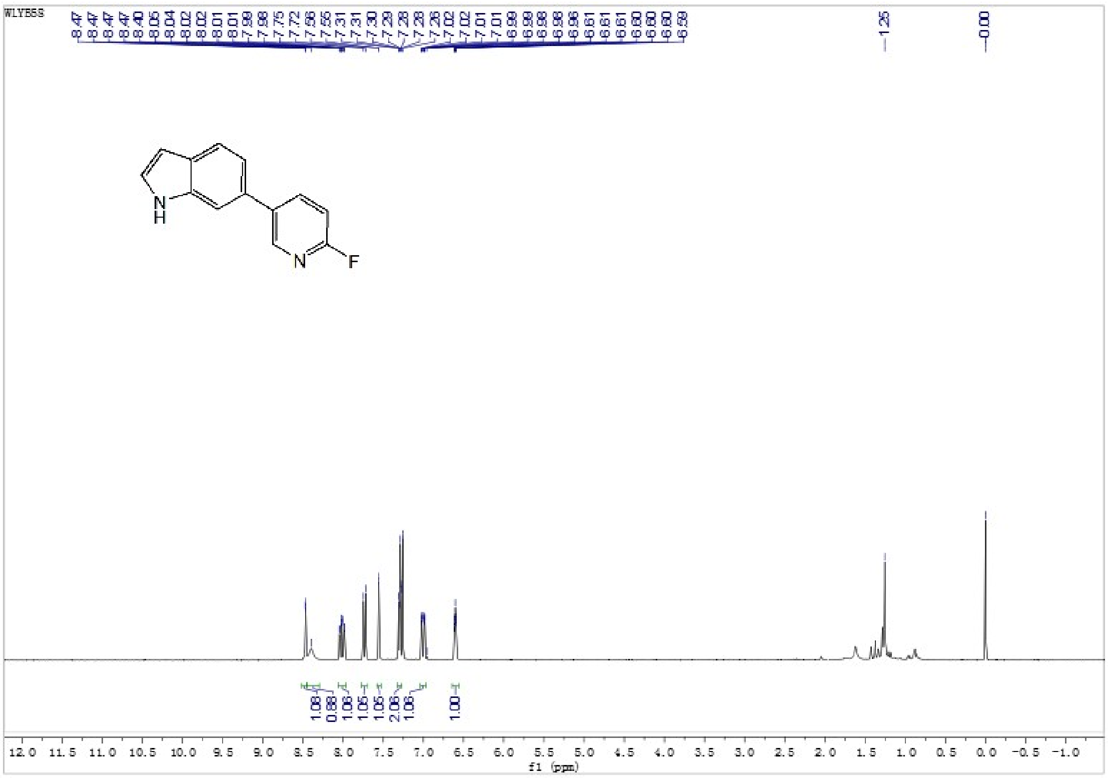

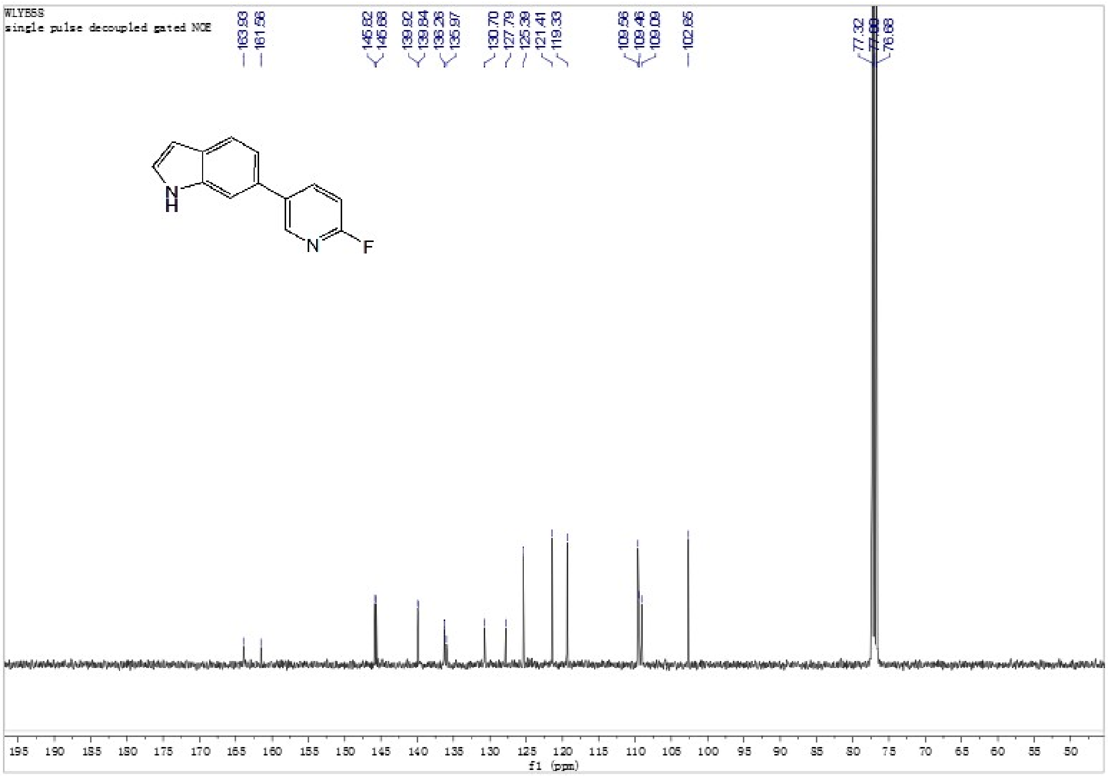

^{1}H and ^{13}C NMR spectra of compound 6g

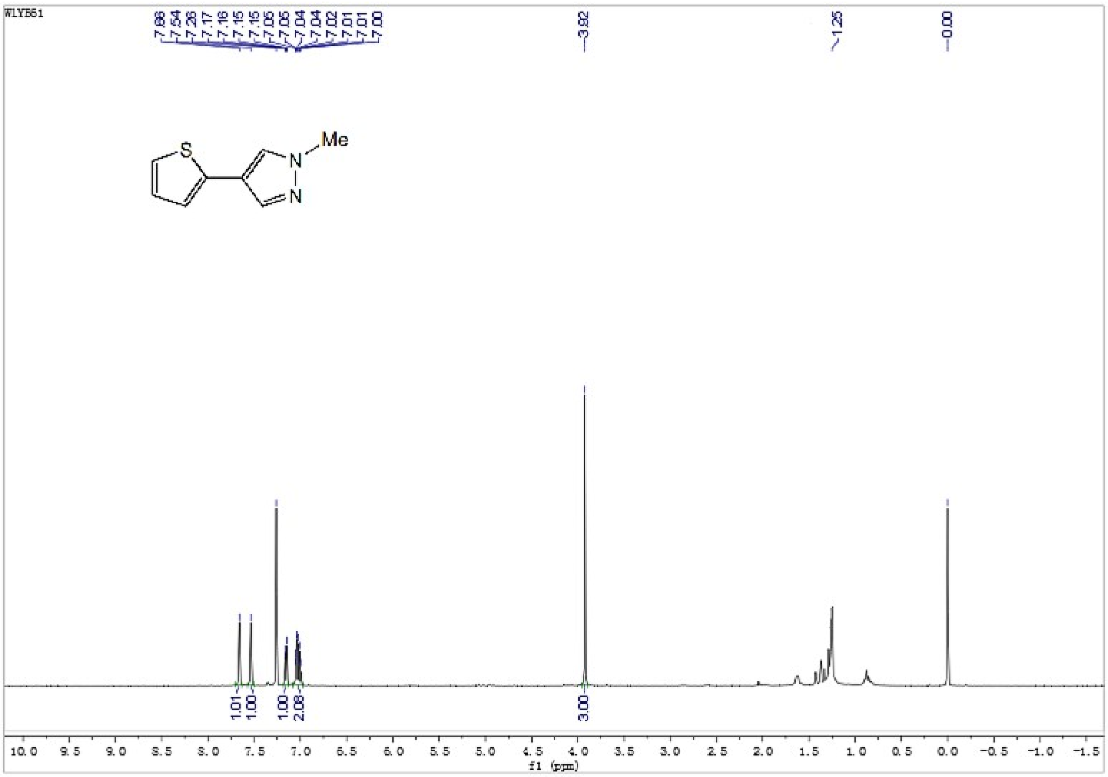

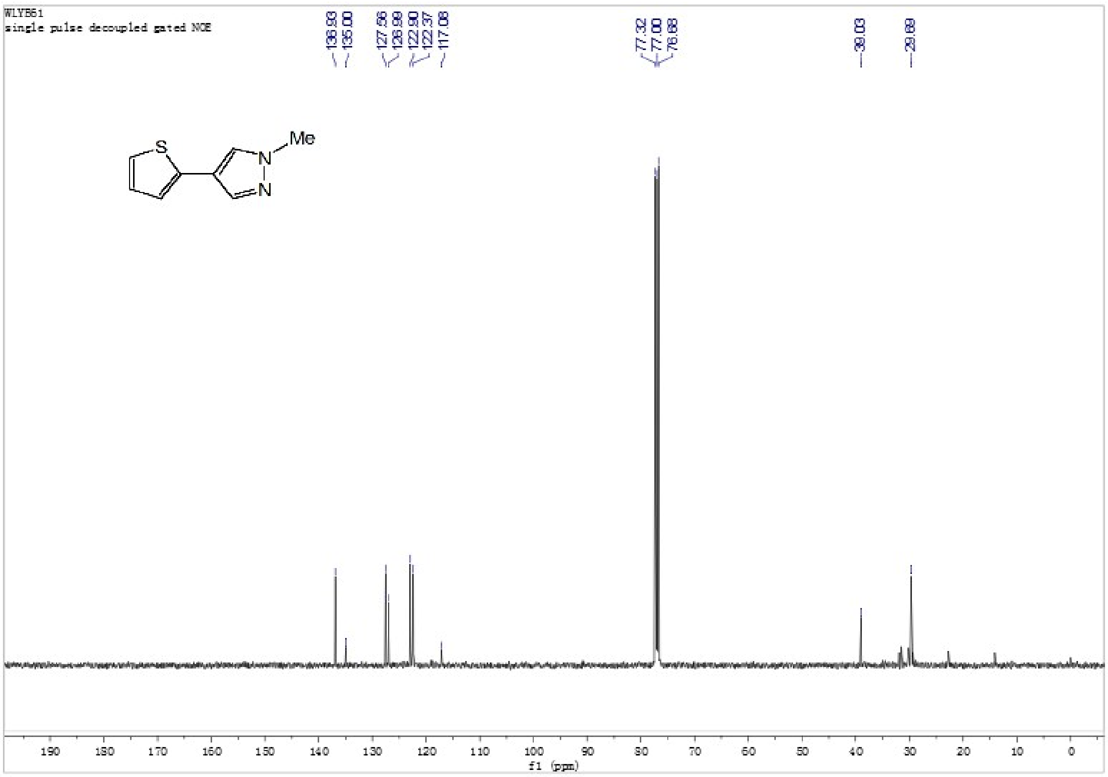
WLYB61
single pulse decoupled gated NOE
136.93
135.00
127.56
126.99
122.90
122.37
117.08
77.32
77.00
76.68
39.03
29.69
Me
S
N
N
190
180
170
160
150
140
130
120
110
100
90
80
70
60
50
40
30
20
10
0
f1 (ppm)
